# Evolutionary paths that link orthogonal pairs of binding proteins

**DOI:** 10.21203/rs.3.rs-2836905/v1

**Published:** 2023-04-20

**Authors:** Ziv Avizemer, Carlos Martí-Gómez, Shlomo Yakir Hoch, David M. McCandlish, Sarel J. Fleishman

**Affiliations:** Weizmann Institute of Science; Cold Spring Harbor Laboratory; Weizmann Institute of Science; Cold Spring Harbor Laboratory; Weizmann Institute of Science

**Keywords:** Epistasis, evolution, protein design, Rosetta, protein-protein interactions, orthogonality, fitness landscape

## Abstract

Some protein binding pairs exhibit extreme specificities that functionally insulate them from homologs. Such pairs evolve mostly by accumulating single-point mutations, and mutants are selected if their affinity exceeds the threshold required for function^1–4^. Thus, homologous and high-specificity binding pairs bring to light an evolutionary conundrum: how does a new specificity evolve while maintaining the required affinity in each intermediate^5,6^? Until now, a fully functional single-mutation path that connects two orthogonal pairs has only been described where the pairs were mutationally close enabling experimental enumeration of all intermediates^2^. We present an atomistic and graph-theoretical framework for discovering low molecular strain single-mutation paths that connect two extant pairs and apply it to two orthogonal bacterial colicin endonuclease-immunity pairs separated by 17 interface mutations^7^. We were not able to find a strain-free and functional path in the sequence space defined by the two extant pairs. By including mutations that bridge amino acids that cannot be exchanged through single-nucleotide mutations, we found a strain-free 19-mutation trajectory that is completely functional *in vivo*. Despite the long mutational trajectory, the specificity switch is remarkably abrupt, resulting from only one radical mutation on each partner. Each of the critical specificity-switch mutations increases fitness, demonstrating that functional divergence could be driven by positive Darwinian selection. These results reveal how even radical functional changes in an epistatic fitness landscape may evolve.

Cellular signaling and metabolism rely on high interaction specificity^8^. Achieving high specificity may appear particularly challenging among pairs of proteins that exhibit sequence and structural similarity. Yet, such homologous pairs are, in fact, often functionally isolated, thereby establishing multiple orthogonal interactions^7,9,10^. The challenge to understanding how orthogonality is achieved among homologous pairs is exacerbated when considering that the evolution of a functionally orthogonal pair requires changes that destabilize interactions between the derived pair and its ancestor. To avoid being purged by evolutionary selection, however, the changes that destabilize the interaction with the ancestral partners must accumulate in an order that maintains binding between the two members of the derived pair following every change^11^. How the proteins in a pair coevolve to exhibit high affinity while disabling their interactions with the ancestral proteins remains a key question in understanding diversification of gene families and protein interaction networks^12–14^.

Some commonly used approaches to address questions on the evolution of binding pairs use ancestral-sequence reconstruction, laboratory evolution, and deep mutational scanning^2,3,12,15–21^. These strategies provided important insights on some of the constraints that shape evolutionary trajectories that lead to new or improved activities^12,22,23^. To date, however, reconstruction of an evolutionary path among orthogonal pairs has been challenging and accomplished only when the number of mutations between the pairs was small enough to allow complete experimental enumeration^2^. Thus, fundamental questions in molecular evolution of orthogonal pairs have remained understudied^11,24,25^, among them: Do mutations on one side of the interface enable mutations on the other? Do evolutionary intermediates present dual-specificities or are the intermediates specific as observed in the extant pairs? Do specificity switches occur gradually through the accumulation of many mutations or abruptly through a handful? And how, despite the need to accumulate destabilizing mutations, are inactive intermediates avoided?

We focused on bacterial colicin endonuclease/immunity pairs^7^. The colicins are SOS-induced nonspecific DNA endonuclease toxins (ColE) that coexpress with cognate high-affinity immunity proteins (Im) that protect the producing cell from degradation of its genomic DNA. Following secretion of this complex from the producing cell, the ColE receptor-binding domain attaches to the outer-membrane target receptor of a neighboring cell. Next, the cytotoxic endonuclease domain (E) is unfolded, dissociating from the protective Im protein, and is then translocated to the host cytoplasm. In the host cytoplasm, the E refolds and degrades DNA, killing the host cell unless the host harbors a cognate and protective Im^7,26^. Cognate E/Im pairs exhibit ultrahigh binding affinity (K_D_ ≤ 10^− 14^ M) and Im proteins that bind E at K_D_ > 10^− 10^ M are not protective^27^. During evolution, the colicins have diversified to four homologous pairs that exhibit ultrahigh specificities of 5–10 orders of magnitude with respect to one another^6,27,28^. Thus, the four Im proteins protect the producing cell against the toxicity of their cognate E proteins but provide no protection against the homologous noncognate E proteins presenting a clear example of functional orthogonality under strong evolutionary selection. Due to their remarkably high affinities and specificities, the colicin E/Im pairs have served as model systems to study evolutionary dynamics^3,29^ and biomolecular recognition in protein engineering and design^6,30–35^. Insights from these studies and the straightforward approach to test the evolutionary fitness of pairs *in vivo* within their native host attracted us to use this system to study the coevolution of orthogonal binding pairs. Because only four E/Im pairs are known^7,36^, however, ancestral sequences cannot be reliably reconstructed as in other systems^4,13,21,22^. Here, we develop an atomistic strategy that models the stability of each possible intermediate in the complete combinatorial binding landscape and searches for a single-mutation path between the extant pairs while minimizing molecular strain that may arise from protein instability or incompatible interactions between the partners.

## Orthogonality and structural incompatibilities

**Figure 1:**
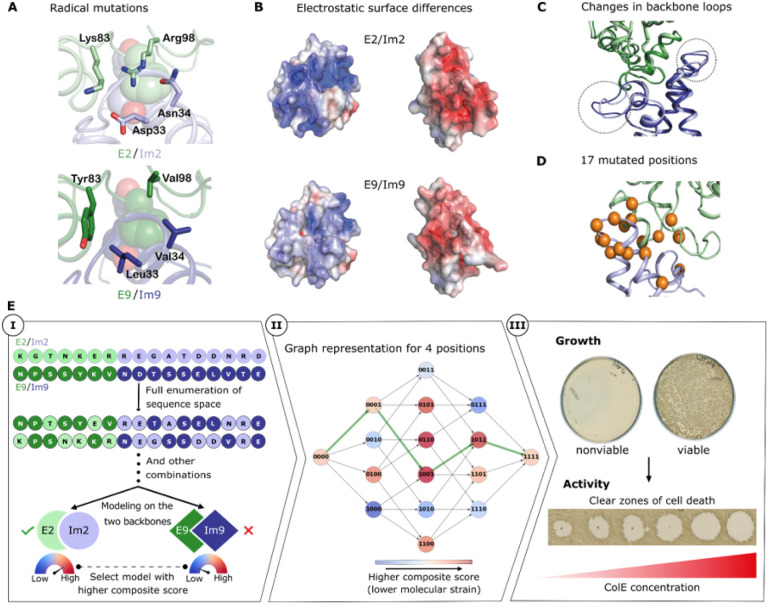
The structural basis for orthogonal binding specificities in colicin E2/Im2 and E9/Im9 and an energy-based strategy to discover evolutionarily plausible paths. (A) Four radical mutations between E2/Im2 and E9/Im9 (PDB entries: 3u43 and 1emv, respectively). The conserved hotspot residues are shown in spheres. (B) Open-book representation of the two complexes with solvent-accessible surfaces colored according to electrostatic representation. (C) Aligned structures of E2/Im2 and E9/Im9. Two loops that exhibit backbone conformation differences are marked in dashed circles. (D) 17 interfacial positions that differ between E2/Im2 and E9/Im9 are represented as orange spheres on the structure of E2/Im2. (E) (**Step I**) Starting from the two wild type pairs, all possible sequences are modeled on both structures. For each sequence, the modeled backbone with the lowest strain (best score) is selected. (**II**) An exemplary subgraph for four positions. Mutants (represented by nodes) are connected if they differ by one mutation. ‘0’ and ‘1’ represent the start and end amino acid identities. The minimum-strain path between the terminal nodes is marked in green. (**III**) Two-step experimental validation of the calculated minimum-strain path. First, we test the growth of bacteria carrying a plasmid encoding a mutant pair (**Fig. S1**). Second, we purify the ColE/Im complex and apply it in tenfold serial dilutions to naive JM83 *E. coli* cells. Clear zones of cell death indicate ColE toxicity.

We used the two most homologous E/Im pairs, known as pairs 2 and 9, as the subjects of our study (PDB entries, 3u43 and 1emv, respectively). In these pairs, the interaction hotspot region, comprising E:Phe86 and Im:Tyr54-Tyr55 (Fig. 1A), is responsible for a large portion of the binding energy and is conserved in sequence and conformation (amino acid numbering according to E2/Im2^34^). Conversely, other interfacial positions contribute less to affinity but determine binding specificity and are therefore the focus of our analysis^27,35^. Some of these positions exhibit dramatic changes in their physicochemical properties. For instance, in E9/Im9, interfacial positions surrounding the hotspot include Val98, Tyr83 (E9) and Leu33 and Val34 (Im9) compared respectively to Arg, Lys, Asp, and Asn in E2/Im2 (Fig. 1A). The overall effect of these four radical mutations is a striking change from a polar and charged interface in E2/Im2 that is mediated by a buried interfacial electrostatic interaction between E:Arg98 and Im:Asp33 to a largely hydrophobic one in E9/Im9 (Fig. 1A **& B**). Additionally, the Im protein backbone differs in two unstructured regions connecting helices 1 and 2 and helices 3 and 4 (Fig. 1C), both containing critical specificity-determining positions^27,34^. Thus, despite the high sequence and structure similarity between the two pairs, there are significant spatial and electrostatic differences in the binding interfaces that underlie the ultrahigh specificity barrier between them. These physicochemical differences also highlight the remarkable challenge that natural selection must have overcome in finding a mutational path that exchanged mutually incompatible networks of molecular interactions across two sides of a binding interface while minimizing molecular strain in every intermediate.

## Finding minimum-strain paths

We develop an atomistic modeling framework to discover coevolutionarily plausible (single-amino acid change) paths that connect two extant binding pairs through functional intermediates. The interfaces of E2/Im2 and E9/Im9 differ by 17 interfacial positions (7 and 10 positions on E and Im, respectively) (Fig. 1D and **Table S1**). The sequence space that encompasses both wild type pairs comprises all combinations of mutations at these interfacial positions, for a total of 2^17^ (approximately 131,000) possible mutants. In an evolutionarily plausible path, each intermediate must exhibit low molecular strain to be functional and confer viability on the producing cell. Therefore, we score each mutant according to its system and binding energies using the Rosetta all-atom energy function^37^. These energies are then transformed using a logistic function into values between 0 and 1 that approximate the likelihood that the proteins are stable and exhibit high affinity, and these values are combined into a composite score through a “fuzzy”-logic AND gate^32^ that estimates whether both energies are sufficiently favorable. To partly address the possibility that the backbone deforms due to mutations, we model each mutant in the context of the two wild type molecular structures (which differ mainly in two interfacial Im loops; Fig. 1C) and choose the best-scoring model to represent each mutant (Fig. 1E, step I). Finally, to represent all mutational paths that traverse the sequence landscape, we construct a directed graph in which the nodes represent all mutants and edges connect nodes that are separated by a single mutation. The edges are weighted according to the negative logarithm of the composite score of the predecessor node (Fig. 1E, step II). Thus, any path that connects the terminal nodes representing the extant pairs is a single-mutation trajectory by construction, and the sum of edge weights represents molecular strain along the path (lower weights correspond to lower strain). We then use a shortest-path algorithm^38^ to find minimum-strain paths (see Methods).

In addition to the 17 interfacial mutations, the two colicin pairs differ by 33 mutations outside the interface. To establish a common genetic background for assessing the evolutionary intermediates, we tested a variant of E9/Im9 that encodes all of the E2/Im2 mutations outside the binding interface (termed E9/Im9_chimera_) (Table S2). We confirmed that E9/Im9_chimera_ was as viable as E9/Im9 (Fig. S3) and that its endonuclease toxicity remained equivalent to that of the two wild type pairs (Fig. S2). Additionally, the chimeric Im protein maintained and even exhibited somewhat improved neutralization of E9 and E9_chimera_ (**Fig. S3**). From this point, we used wild type E2/Im2 and E9/Im9_chimera_ as the genetic backgrounds for testing intermediates, allowing us to focus only on the interfacial mutations.

As a first test of the computational framework, we generated three graphs comprising only the sequence space defined by the two extant colicin pairs (binary sequence space; see Methods). We scored the mutants using the Rosetta talaris energy function^39^ and computed minimum-strain paths. All the paths exhibited unfavorable energies in several intermediates relative to the extant endpoints (**Fig. S4**) suggesting that some strain might be inevitable in the binary sequence space. Experimental viability screening in *E. coli*, which probes the ability of the bacteria to produce a high-affinity complex (Fig. 1E, step III), demonstrated that in each path, at least seven intermediates were nonviable (Fig. S4). We concluded that these paths do not represent evolutionarily plausible trajectories.

## Codon-bridging mutations reduce molecular strain

**Figure 2:**
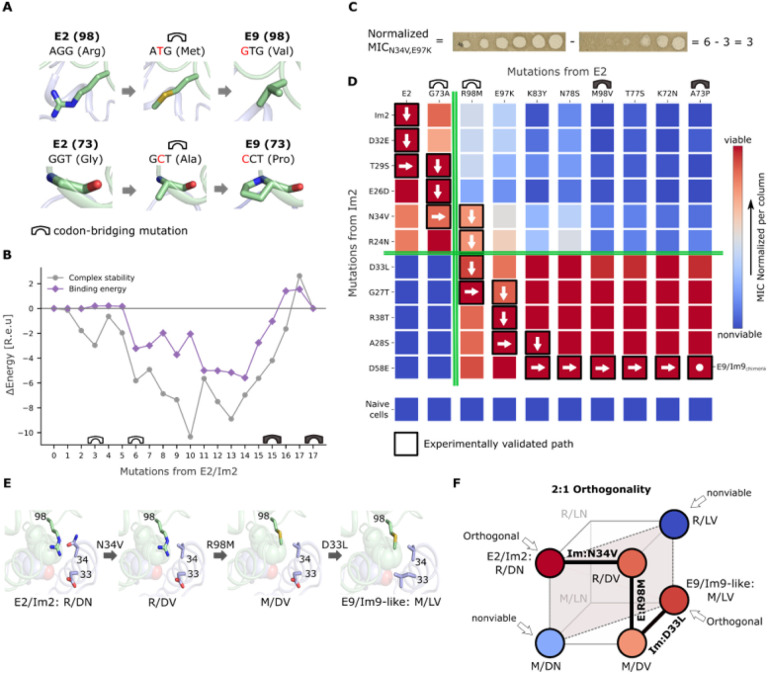
Experimental validation of an evolutionarily plausible path linking two orthogonal binding pairs. (A) Examples of codon-bridging mutations. DNA nucleotide mutations are indicated in red. (B) Binding and stability energy differences of the intermediates in the minimum-strain path relative to the two wild type pairs. An empty bridge icon represents the introduction of a bridging mutation, and a full bridge icon represents its resolution. (C) An example of the inferred normalized minimal inhibitory concentration (MIC). (D) Functional specificity map. Each endonuclease on the viable path was applied to bacteria harboring each immunity protein. Mutations from E2 accumulate from left-to-right and mutations from Im2 accumulate from top-to-bottom. A vertical green line marks the specificity switch on E and a horizontal one marks the specificity switch on Im. The mutations are completely reversible, and arrows are only visual guides. The MIC scores within the map are normalized to the minimal and maximal killing (MIC on naive cells) of each E (in each column), and higher viability corresponds to higher normalized MIC. The scores within the map are an average of two technical repeats (raw averages and standard deviations in **Table S5**). (E) Molecular models of the key mutations producing the orthogonality along the experimentally validated evolutionary path consistent with the model in panel F. The hotspot is represented in spheres. (F) General model of orthogonality in a bimolecular fitness landscape involving two sites in one molecule (Im protein) and one site in the other molecule (E toxin) defining what we call a 2:1 orthogonality model (see Supplementary Information and **Fig. S9**). The corners of the cube represent all the possible pairs of sequences, and colors are consistent with panel D. Thick lines represent the empirical path and dotted lines join the pairs whose fitness determines orthogonality of the pairs for interacting molecules.

We therefore expanded the allowed sequence space beyond the identities observed in the two extant pairs. Since simultaneous multinucleotide mutations are less likely to occur in evolution compared to single-nucleotide mutations^40^, we assessed whether some interface mutations require several nucleotide exchanges. We found that seven out of the 17 interfacial mutations required at least two nucleotide exchanges between the identities in the two pairs (**Table S1**) suggesting intermediate mutations that could have bridged the two extant identities. We analyzed homologs of colicin pairs 2 and 9 to find indications for bridging identities but found almost no interface mutations in the natural diversity of homologs (**Table S3 and S4**). We therefore chose the codon bridges based solely on physicochemical properties. As the sequence space that includes all codon bridges is too large for atomistic modeling (>10^8^ intermediates), we selected at each position one bridging mutation that was physicochemically intermediate between the two extant identities (**Table S1**); for example, in position E:98, we added Met to the two extant identities, Arg and Val, and in E:73, we added Ala to the extant identities, Gly and Pro (Fig. 2A). We did not force the paths to go through the bridging mutations, however; instead, we retained direct connections representing all amino acid substitutions and at these seven positions, added edges through the single-nucleotide bridging mutations. To compute stability and binding energy, we used the newer ref15 energy function, which embodies a physically more accurate description of polar interactions^41^ than talaris. We also computed a reference path using this energy function but without including the codon bridges in the graph (**Fig. S5**).

The energy profiles of the optimal path with bridging mutations were substantially lower than those of any of the other four paths without codon bridges (the reference path and the three paths that were built using talaris) (Fig. 2B, S4, and S5). Throughout most of this trajectory, the computed energies were lower than those of the wild type pairs, rising slightly above them in the few final mutants that adopt the backbone conformation of E9/Im9 (Fig. 2B). Remarkably, we found that all 18 intermediates were viable, and that all endonuclease intermediates retained toxicity towards naive JM83 cells (**Fig. S6**). We concluded that the minimum-strain path with the bridging mutations is an evolutionarily plausible trajectory linking the two wild type orthogonal colicin pairs through fully functional single-point mutations.

## An abrupt specificity switch

To investigate whether the specificity switch between the two colicin pairs occurs gradually through many small-effect mutations or abruptly through a few strong-effect mutations, we built a functional quantitative specificity map between E and Im variants along the viable path. We applied the eight intermediate ColE mutants, the wild type ColE2 and ColE9_chimera_ in tenfold serial dilutions to JM83 cells transformed with the nine Im intermediates, wild type Im2, and Im9_chimera_. We then visually estimated how protective each Im protein was with respect to each ColE according to the minimal inhibitory concentration (MIC; the tenfold dilution of ColE in which no clear zones were observed; Fig. 2C) and integrated the results into a functional specificity map (Fig. 2D). We also verified that all purified ColE/Im complexes exhibited similar concentrations (approx. 3 mg/ml) (**Fig. S7**) and that Im intermediates were similarly expressed (approx. 2 mg/ml) and stable up to 50 °C (**Fig. S8**). The specificity map shows that, in addition to the computed minimum-strain path, there are > 3,500 single-mutation paths that cross high-viability regions (colored red, see Methods for details on the calculation of the number of paths through these regions). All these paths differ in the order in which E and Im are mutated before and after reaching a particular pair, Im:Arg24Asn/E:Arg98Met (Fig. 2D), which constitutes a bottleneck. Strikingly, the map reveals that orthogonality is preserved along most of the steps in the evolutionary path, and that as few as three substitutions are sufficient to induce a complete switch in specificity. Two of these substitutions take place at consecutive positions in the immunity protein (Asn34Val and Asp33Leu) and one in the E protein (Arg98Met). Im:Asn34Val abrogates a hydrogen bond to E:Arg98 and acts as an enabling mutation, allowing the mutated Im to bind the E:Arg98Met mutant (Fig. 2E). E:Arg98Met completely switches the specificity pattern of the endonuclease, interacting strongly with Im9-like variants but substantially less with the ancestral Im2-like variants (Fig. 2D). Subsequently, Im:Asp33Leu, a known specificity determinant^27,34^, eliminates the Im protein recognition of E2-like endonucleases in favor of E9-like variants and completes the functional transition (Fig. 2D).

These results offer several insights that may generalize to other coevolving systems. First, the dramatic specificity switch occurs in only a few evolutionary steps — similar to observations in several molecular systems, though the path reconstructed here is much longer than any that have been previously characterized^4,21,25^. Second, in Supplemental Information, we study general molecular mechanisms that produce orthogonal binders, concluding that they can be classified by the minimal number of mutations that are required for the emergence of orthogonality (**Fig. S9**). The mechanism of orthogonality in the experimentally validated path is what we call “2:1 orthogonality” (Fig. 2F). In this model, orthogonality necessarily evolves through a strict order of mutations starting with a mutation on one partner, then the other, and finally a mutation in the first partner that interacts with the first mutation, as observed in the empirical path (Im:Asn34Val→E:Arg98Met→Im:Asp33Leu) (Fig. 2E and 2F). Consistent with this proposed mechanism, the two interacting sites in the same molecule (Im:33 and Im:34) form an *intra*molecular epistatic interaction and are indeed spatially close (Fig. 2E). The specificity switch, however, is more complex when viewed relative to the four E mutants that contain the Met98 codon-bridging mutation. Following the E:Arg98Met mutation and until the E:Met98Val mutation (E9 identity), the endonucleases present a reduced specificity barrier for the Im proteins, though they maintain nearly equal toxicity when applied to naive JM83 cells (**Fig. S6**). Thus, although the Im intermediates close to Im2 are less protective than those close to Im9 against endonucleases that carry Met98, the former nonetheless present a significant level of protection (Fig. 2D). The Met98-containing endonucleases are, therefore, multispecific relative to the very strict functional orthogonality observed in all the other endonucleases. E:Arg98Met is thus not only a specificity watershed but may also be an enabling mutation, lowering the specificity barrier and allowing the accumulation of additional E and Im mutations. Once the Val mutation is fixed, the specificity barrier is, once again, extremely strict. The case presented in this evolutionary trajectory is one in which a minimal number of large-effect mutations (two) establishes a specificity watershed, yet many surrounding mutations are needed to stabilize and enable the pair of mutations^42^.

## Stepwise resolution of epistatic interactions

Next, we examine the minimum-strain path with respect to a computed fitness landscape of all possible E/Im pairs. To generate this fitness landscape, we used the empirical data from the specificity map (including cognate and noncognate pairs from Fig. 2D) to calibrate a predictor of normalized MIC scores (Fig. 2C) based on the Rosetta binding energy of each pair (Pearson *r* = 0.68 in leave-one-out cross-validation, **Fig. S10**, see Methods). We then predicted normalized MIC scores for all 2.2 million possible E/Im pairs (including all codon bridges) and applied a technique for visualizing fitness landscapes^43^ to produce a low dimensional representation in which distances between pairs approximate the time it may take for a population to evolve from one pair to another under purifying selection for high normalized MIC score (see Methods).

**Figure 3:**
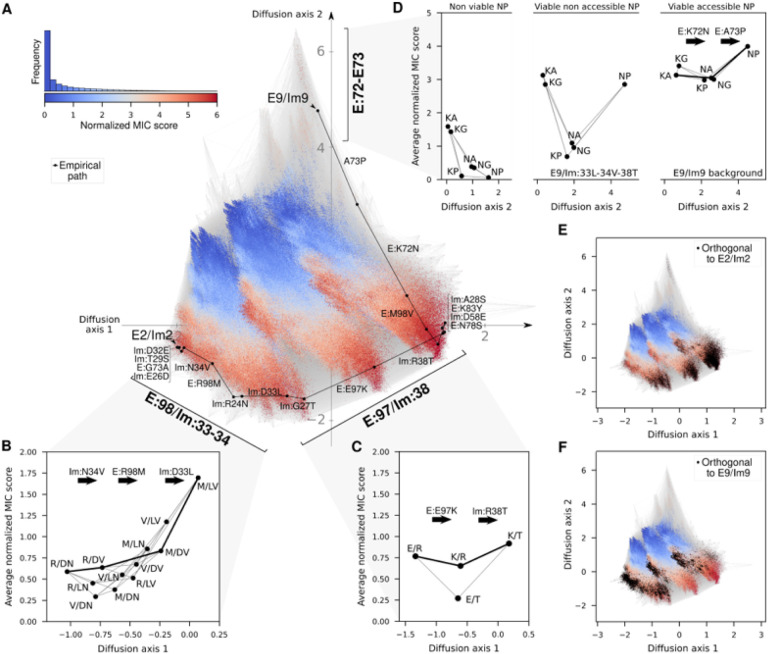
Computed fitness landscape highlights the potential role of multiple epistatic interactions in effecting the specificity switch. **(**A) Visualization of the predicted fitness landscape. Distance between points represents the time to evolve between E/Im pairs under selection for high normalized MIC scores (see Methods). Nodes represent each possible protein pair and edges connect pairs that differ by a single amino acid substitution. Node color indicates normalized MIC score. Sequences with higher scores are plotted above to emphasize the relationships among functional sequences. Empirical path is highlighted in black. Inset shows the distribution of predicted normalized MIC scores across all possible pairs together with the color scale. Interactions between key mutations are marked in the visualization and simplified in panels B-D depicting the networks of average MIC scores defined by the identities at the key sets of interacting positions. (B,C) Diagrams representing the average MIC score and average position at Diffusion axis 1 for each possible subsequence at positions E:98/Im:33–34 (B) and E:97/Im:38 (C). The path taken by the empirical trajectory across each set of sites is highlighted by a thick line. (D) Diagrams representing the average MIC score and average position at Diffusion axis 2 for each possible subsequence at positions E:72–73 on the left, and fixing the specified positions in the center and right plots. (E,F) Visualization of the fitness landscape with overlaid black dots indicating pairs that are predicted to be orthogonal to pair E2/Im2 (E) and E9/Im9 (F) (orthogonal to E2/Im2 defined as pairs with predicted normalized MIC scores above 3.5 but whose combination with both Im2 and E2 had predicted normalized MIC score below 3 and *vise versa* for E9/Im9).

The visualization reveals that the predicted fitness landscape is shaped by three main sets of molecular interactions that separate sequences along the “diffusion axes” (see Methods) that define our low dimensional representation (Fig. 3A). The first interaction spreads sequences diagonally across Diffusion axes 1 and 2 and is mainly explained by incompatibilities between mutations in the specificity-switching positions E:98, Im:33 and Im:34 revealed by the experimental specificity map (Fig. 2D). Averaging out mutations in all other positions (Fig. 3B) shows that several possible fit combinations of mutations link the E2/Im2 sequence E:Arg98/Im:Asp33,Asn34 to the combination E:Met98/Im:Leu33,Val34 that has E9/Im9-like specificity in the empirical specificity map (Fig. 2D). By contrast, other combinations are predicted to have low average function, *e.g*. E:Met98/Im:Asp33,Asn34, a combination which was experimentally observed to be nonfunctional (Fig. 2D). Such low-function combinations separate the high-functioning ones in the landscape. Note that that E:Met98/Im:Leu33,Val34 is predicted to be the fittest possible subsequence at these positions (Fig. 3B), explaining why the minimum-strain path constructs this combination early and maintains it for most of its length.

The second interaction also spreads sequences diagonally across Diffusion axes 1 and 2, separating the fitness landscape into three main groups of sequences due to an incompatibility between mutations at positions E:97 and Im:38. In E2/Im2, these positions (E:Glu97/Im:Arg38) form another saltbridge across the interface (**Fig. S11A**), whereas in pair 9 (E:Lys97/Im:Thr38) there is a polar contact between the sidechains (**Fig. S11A**). This separation is driven by the very low average fitness of variants with one of the intermediate mutational states, *i.e*., E:Glu97/Im:Thr38, where E:Glu97 is not paired to a countercharge and therefore strained (Fig. 3C & S11A). Finally, the third interaction spreads sequences vertically along Diffusion axis 2 due to an *intra*molecular high-order epistatic interaction involving positions E:72 and E:73. The E9 identities (Asn72, Pro73) are highly deleterious in most sequence backgrounds (Fig. 3D, left). Structure models show that this pair of identities is compatible only with the backbone and rigid-body orientation observed in E9/Im9 and in a sequence context characterized by E9 and Im:Leu33-Val34-Thr38 (Fig. 3D, center and **Fig. S11B**). In this latter context, however, all possible intermediate states are nonviable, resulting in a fitness valley that can only be avoided in the E9/Im9 context and conformation (Fig. 3D, right and **S11B**). The E:Asn72-Pro73 pair of mutations can only be implemented once almost all positions have been mutated to the E9/Im9 identities, enabling the backbone change to E9/Im9. While our analysis was performed assuming a quantitative fitness function (normalized MIC score), the characterized genetic interactions remain robust even when using a threshold-like fitness function along the whole range of reasonable cutoffs on the normalized MIC score or potential inaccuracies in identifying the truly functional sequences (**Fig. S13**). This robustness is likely due to the fact the major aspects of the landscape structure depend primarily on a small set of grossly incompatible combinations of mutations (such as E:Glu97/Im:Thr38), and that correctly identifying these incompatibilities does not depend strongly on the details of the molecular force fields or the precise choice of fitness function.

The global view of the predicted fitness landscape offers several insights that were elusive in the empirical specificity map of Fig. 2D. First, we find that the empirically validated path changes specificity abruptly after a few mutations (**Fig. S12**) and minimizes strain by having at most one marginally stable region of the complex at a time rather than superimposing incompatibilities (Fig. 3A, see also Fig. 2D). Second, by overlaying pairs that are orthogonal with respect to E2/Im2 or E9/Im9, we find that each forms a contiguous sequence subspace that would serve as an entry point to orthogonality (Fig. 3E,F), regardless of the thresholds used to define orthogonality (**Fig. S14–15**). The entrance into orthogonal sequence subspaces suggests that once the specificity switching mutations are implemented, many different paths can lead to the orthogonal endpoint. Third, understanding how orthogonal E/Im pairs are arranged relative to each other in sequence space can help us characterize co-existing mechanisms that result in orthogonality beyond the one shown in the empirical path (see Supplementary Information, **Fig. S9**). Overall, these results shed light on the global structure of the fitness landscape and the constraints that shape the evolution of orthogonality in pairs of interacting molecules, including predicted epistatic interactions that were not apparent from the experimental analysis of the empirical path (*i.e*., E:97/Im:38, E:72–73).

## Adaptive mutations may drive functional divergence

The atomistic and graph-based calculations we employed are based solely on the physical constraints that a generic protein pair must meet to retain binding affinity. If these were the only determinants of fitness, we would expect natural populations to mutate neutrally among functional pairs. The fact that the pair forms a toxin-antitoxin system, however, can influence how populations evolve and functionally diverge. In this case, fitness depends not only on the ability of the immunity protein to neutralize its cognate endonuclease, but also on whether the immunity protein can neutralize other endonucleases existing in the population and whether the cognate endonuclease can escape recognition by other immunity proteins, killing competing strains.

**Figure 4:**
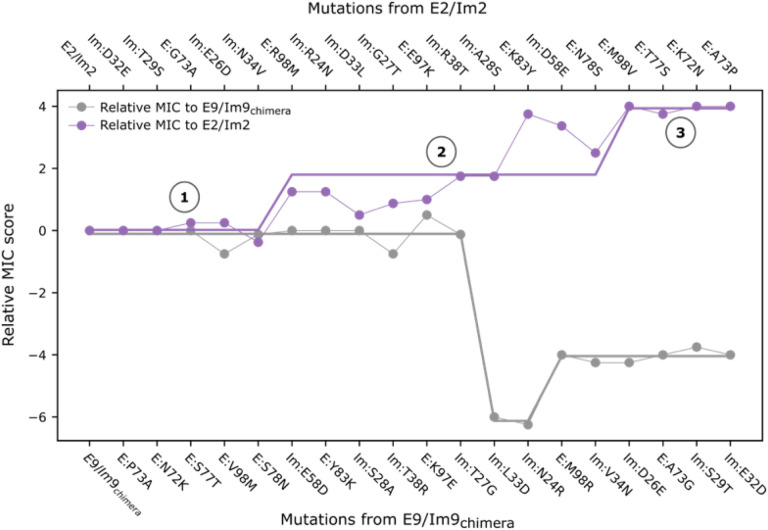
A “ratchet-like” model for the evolution of ultrahigh specificity. The experimentally determined viability of each cognate pair on the path relative (Fig. 2D) to the extant E2/Im2 (purple line, top *x*-axis) or E9/Im9 (gray, bottom *x*-axis). Viability increases in two steps in the direction of accumulating mutations from E2/Im2 and decreases in the reverse direction. Straight lines are guides to the eye. Higher viability corresponds to higher normalized MIC score. Relative MIC score (relative viability score) is the difference in MIC scores between a mutant and the parental wild type: RM_i_= (M_E(*i*)/Im(*i*)_ - M_E(*i*)/Im(*wt*)_) - (M_E(*wt*)/Im(*wt*)_ - M_E(*wt*)/Im(*i*)_), where RM is the relative MIC score; M is the MIC score (see Methods); *i* is an index to the mutant; *wt* is the parental protein, E2/Im2 or E9/Im9_chimera_.

Given these considerations, we re-examined the functional specificity map (Fig. 2D) to investigate the direction, if any, in which the experimental path is adaptive or maladaptive. We tracked how the relative MIC score (Methods) changes along the path with respect to each extant pair. In this simplified analysis, an intermediate exhibits high fitness relative to the parental population if its Im protein resists the parental endonuclease and its endonuclease escapes resistance by the parental Im protein (Equation in Fig. 4). While any sequence in the path could act as a hypothetical ancestor, we started by assuming that evolution begins at the E2/Im2 pair or mutants that are phenotypically similar, and that it proceeds in a population dominated by such pairs (Fig. 4, purple line, Step 1). After a series of neutral mutations that do not affect binding specificity, the enabling mutation Im:Asn34Val expands the repertoire of endonucleases that the Im can neutralize, including E:Met98. At the same time, Im:Asn34Val maintains binding affinity towards the ancestral E2. The following mutation, E:Arg98Met (Step 2), introduces an E variant that is, for the first time, cytotoxic towards the parental bacteria harboring an Im2-like protein while its cognate Im protein protects against E2-like endonucleases (Fig. 2D). Thus, the E:Arg98Met mutation is not only tolerated but may have an adaptive advantage that would allow it to penetrate, persist and spread within the parental population. The E:Arg98Met mutant, however, does not form an optimal interaction with the Im protein prior to incorporating the Im:Asp33Leu mutation, and the Met98-containing endonucleases are only partially cytotoxic towards Im2-containing hosts (Fig. 2D). Thus, Im:Asp33Leu improves the viability of this pair relative to the E:Arg98Met, and in Step 3, the codon-bridging mutation is resolved to the E9 identity through E:Met98Val. At this point, the endonuclease gains high cytotoxicity against the Im2-like harboring bacteria and completes the functional transition. In contrast, in a population dominated by an E9/Im9-like pair, the reverse trajectory passes through at least one maladaptive step (Fig. 4, gray line): Im:Leu33Asp reduces viability relative to the Im9-like proteins and is strongly susceptible to killing by parental E9-like endonucleases (Fig. 2D). In this simplified analysis, the adaptive mutations may provide a “ratchet”-like mechanism in which each adaptive step reduces the likelihood of the reverse mutation and increases the likelihood of accumulating further function-switching mutations.

There are limitations to our analyses, however. Although viability was measured *in vivo* and in the native host, experimental measures of functionality may not be completely faithful to viability in a wild population in nature. Despite these limitations, our results provide an example of how the specific nature of a protein-protein interaction, *i.e*., a toxin-antitoxin system, may influence evolutionary paths, transforming what are *a priori* neutral mutations (with respect to binding) into adaptive or deleterious ones depending on the dominant genotypes in the population.

## Discussion

We showed how a charged protein-protein interface could transform, one-mutation-at-a-time, into a largely hydrophobic one without compromising stability, affinity and catalytic activity below their functional threshold in any intermediate step. To achieve this, we found that a codon-bridging mutation temporarily reduces the specificity barriers, permitting mutations that would otherwise reduce stability or binding affinity. Unlike previously reconstructed specificity switches where the intermediates were promiscuous “generalist” binders^2^, however, here the specificity switch occurs suddenly, with the key bridging mutation E:Arg98Met eliminating the interaction with Im2 and creating a new interaction with Im9 in a single step. While the structure of the genetic code is known to constrain the possible paths to evolve a new specificity^12^, our results provide an example in which the extra biochemical diversity provided by bridging mutations is actually essential to obtain a viable path between the orthogonal pairs.

A key question in the evolution of orthogonal binding pairs is how ultrahigh specificity evolves by a single-mutation trajectory without crossing a fitness valley. Our results provide a case-study in which each of the specificity-switching mutations are not only tolerated but may endow their host with a selective advantage relative to the parental population. This polarizes the function-altering evolutionary process, increasing the likelihood of selecting a long series of mutations, whereas the reverse mutations are counterselected. In other words, the functional asymmetry in the toxin-antitoxin system suggests preferred directions for the evolutionary process depending on specific environmental conditions.

This work introduces energy-based modeling as an approach to study both the overall shape and the fine details of a highly epistatic fitness landscape. The approach is general and can be implemented, in principle, to suggest evolutionarily plausible trajectories that link wild type proteins or inferred ancestral states, complementing and extending existing experimental and computational methods^2,3,12,15–19^. It may provide detailed mechanistic insights on the evolution of microbial resistance mutations and the evolution of new activities in enzymes and binders.

## Methods

### Atomistic modeling

We enumerated all possible combinations of mutations and modeled them in Rosetta on the backbones of E2-Im2 and E9-Im9 (PDB codes: 3u43 and 1emv, respectively). Each mutant was modeled using all-atom Rosetta calculations, including combinatorial sidechain packing, and backbone and side chain minimization subject to harmonic restraints on the Cα coordinates^44^. Each mutant was modeled on the structures of both E2-Im2 and E9-Im9. All intermediates were ranked according to a composite score based on “fuzzy”-logic design (*W*)^32^ (Eq. 1) accounting for the difference of all-atom energy (*ΔΔG*_*s*_) and difference of binding energy (*ΔΔG*_*b*_) compared to the two wild type sequences.

(1) $$W=-\text{ln}\left(\frac{1}{1+e^{0.7\times \left(\Delta \Delta G_{s}-5\right)}}\right)-\text{ln}\left(\frac{1}{1+e^{0.7\times \left(\Delta \Delta G_{b}-5\right)}}\right)$$

For each sequence, the higher (more favorable) composite score was selected from the two calculations.

### Finding a least-frustration path using a graph-theoretical shortest-path algorithm

The sequence space comprised approximately 130,000 sequences in the initial four formulations (using a binary sequence space) and 2.4 × 10^6^ for the sequence space with codon-bridging mutations. We computed a truncated graph by taking the top 5% nodes with the highest composite scores. Nodes were connected by an edge if they differed by one amino acid substitution and contained all the previous mutations. The weight of each edge was assigned as *W* (Eq. 1) of the preceding node. Then, the Bellman-Ford shortest-path algorithm^38^ was applied to find the path with the lowest sum of scores (least strained). In the graph that contained amino acids that served as DNA bridging mutations, edges were added to connect mutants that could not be crossed with single-nucleotide mutations.

In the preliminary work that resulted in the four low-strain paths that were not viable (**Fig. S4** and **S5**), the graphs were constructed using somewhat different ways but with the same scoring rules as described above. Briefly, in (1) the interface was divided into three spatially separated modules. Then the shortest path was found inside each module and between the modules. In (2) intermediates that exhibited more than 7 R.e.u. difference in system energy were eliminated. (3) intermediates with binding energy difference to wild type or binding strain difference to wild type above 2.5 and 1.5 R.e.u. respectively were eliminated and a graph was formed with the remaining nodes. In this trial, we applied another Rosetta filter, BindingStrain, which calculates the rotameric strain of monomers upon binding^45^. The intermediate scores in those three paths were calculated using Rosetta energy function, talaris14. And in (4) the graph was formed as the graph that contains the bridging mutations, but after eliminating the bridging nodes. In this fourth graph and the one with bridging mutations we used the new Rosetta energy function, ref^15^, to model and rank the intermediates. This energy function improves the treatment of electrostatics and solvation relative to the talaris14 energy function we previously used for the first three paths^37^.

### Number of viable paths in the experimental specificity map

We compute the number of viable directed, shortest-path (no reversions) evolutionary trajectories based on the data from the empirical specificity map (Fig. 2D). We note first that the space of viable intermediates in the specificity map is roughly separated by a bottleneck into two large grid-like blocks (2 × 6 and 8 × 5). Eq. 2 computes the number of paths between two corners of an *n* by *k* grid:

(2) $$\frac{(n+k-2)!}{(n-1)!(k-1)!}$$

The total number of paths through both blocks is the product of the number of paths in each block. In addition, another set of paths exit the first block into another intermediate at Im:Asn34Val/E:Arg98Met after only four substitutions in the Im protein, increasing the number of paths in the first block up to 11. We thus get a total of

$$\left(\frac{\left(6+2-2!\right)}{\left(6-1\right)!\left(2-1\right)!}+\frac{\left(5+2-2!\right)}{\left(5-1\right)!\left(2-1\right)!}\right)\times \frac{\left(5+8-2\right)!}{\left(\left(5-1\right)!\left(8-1\right)!\right)}=11\times 330=3,630$$

possible paths.

### Cloning

pET21d plasmid harboring ColE2 wild type gene (full gene including R, T, and C domains) followed by Im2 wild type gene (separated by a 2-bp frameshift) with a C-terminal His_6_-tag^34^ was used as the basis for cloning. Synthetic genes for the two wild types and the intermediates of the computed least-strain path were codon-optimized for efficient *E. coli* expression and custom synthesized as linear fragments by TWIST Bioscience. The genes were swapped with the wild type gene by restriction-free (RF) cloning^46,47^. The plasmids were transformed into T7 Express *lysY/I*^*q*^ *E.coli* cells (NEB), and DNA was extracted for Sanger sequencing to validate accuracy. Validated plasmids of the intermediates were also tested for viability by transformation to BL21 DE3 *E. coli* cells. An intermediate pair was considered viable if a lawn of bacteria was observed in the plate. Three colonies were picked for repeat sequencing to validate that no additional mutations were introduced. Sequence-validated colonies of BL21 *E. coli* were stored in glycerol stock at −80°C for expression and purification.

### Protein expression and purification

BL21 (DE3) cultures were grown in Luria Broth (LB) with 100 mM ampicillin medium at 37°C to OD_600_ = 0.6–0.8 and induced with 1 mM IPTG at 16°C for 20 hrs. Cells were harvested and stored at − 20°C. Pellet was resuspended in 10 ml lysis buffer in 200 ml culture containing 50 mM Tris (pH 8.6), 50 mM NaCl, 10 mM imidazole, 2 mM MgCl_2_, benzonase (1:500), and protease inhibitor (1:1000) sonicated in 3 cycles of 20 sec On and 40sec Off (1 min On-time) and centrifuged as previously described^34^. The supernatant was loaded onto a column packed with 1.2 ml Ni-NTA beads for 200ml culture, equilibrated with lysis buffer, and incubated at 4°C for 1hr for binding, then washed with lysis buffer containing 20 mM imidazole, and eluted with lysis buffer containing 500 mM imidazole. Protein complexes were dialyzed overnight in 50 mM Tris (ph 8.6), 200 mM NaCl, and 2 mM MgCl_2_. Protein complexes were stored at 4°C for a week and for longer times at −80°C.

Im proteins were purified using the same protocol as above with minor changes. The pH for the buffers was adjusted to 7.3, they were grown in 50 ml culture and all quantities were adjusted accordingly. Melting curves for the purified Im proteins were measured using Tyco NT.6 (Nanotemper Technologies Inc) at a rate of 30°C/min.

All protein concentrations were measured using absorbance at 280nm. The concentrations for the ColE/Im complexes range between 2–3.5 mg/ml and Im protein concentrations range between 1.9–3 mg/ml.

### ColE/Im *in vivo* assay (spot-test)

The biological activities of ColE and Im proteins were determined using a modified form of the spot-test described in refs.^48,49^. Briefly, ColE-sensitive *E. coli* JM83 (Addgene) cells were transformed with a control vector (empty pET21d) or one of the Im proteins and incubated in LB containing 100 mM Ampicillin at 37°C overnight. Then, 1 ml of culture was plated on agar plates to create a lawn of bacteria. Purified ColE/Im complexes (concentration between 2–4 mg/ml) were 6 times diluted in 10-fold serial dilutions and 8 μl aliquots were dropped on the plates containing bacteria. Plates were incubated overnight at 37°C. Clear zones represent cell death and the absence of clear zones indicates biological protection conferred by the Im. The minimal inhibitory concentration (MIC; the concentration dilution of ColE in which no clear zones were observed) was set as the last visually apparent drop (0, 1, 2, 3, 4, 5, or 6). The normalized MIC score is then computed as the difference between the maximal killing (the number of drops against an empty plasmid) and the MIC. All viability assays were conducted using two technical repeats and averages and standard deviations are shown in Fig. 2D and **Table S5**.

### Calibrating normalized MIC scores against Rosetta binding energies

In this section, we aimed to define a model that allows us to transform the computationally predicted binding energies for each Im/E pair sequence *i* into the more biologically relevant scale of the normalized MIC score that takes into account the order of magnitude increase in the tolerated concentration of E toxin. Since the Im can only increase but not decrease the tolerance, the normalized MIC score cannot get lower than 0. To take into account this, we model the expected normalized MIC score (*ȳ*_*i*_) as a function of the computationally predicted wild-type normalized binding energy *ΔΔG*_*binding*,*i*_:

$${\bar{y}}_{i}=\text{log}\left(1+e^{\alpha +\beta \Delta \Delta G_{\text{binding},i}}\right)$$

However, we cannot characterize directly *ȳ*_*i*_ but rather fit to data that is characterized by some error. Moreover, as we have data only for six concentrations, our observed normalized MIC score will not be higher than the number of tested concentrations, but the real underlying score could indeed be larger. Thus, we define a piecewise likelihood function for dependence on the observed *y*_*i*_ and the standard deviation of the measurement errors *σ*:

$$p\left(y_{i}|\bar{y_{i}},\sigma \right)=p\left(y_{i}\geq 6|\bar{y},\sigma \right)\text{ if }y_{i}=6$$

$$p\left(y_{i}|\bar{y_{i}},\sigma \right)=p\left(y_{i}\leq 0|\bar{y},\sigma \right)\text{ if }y_{i}<0$$

$$p\left(y_{i}|{\bar{y}}_{i},\sigma \right)=p\left(y_{i}|\bar{y},\sigma \right)\text{ if }0<y_{i}<6$$

The data from the specificity map computed in Fig. 2D shows consistent normalized MIC scores across the two biological replicates (**Fig. S10A**). We fit the model by maximizing the likelihood, that is, by finding the combination of *α*, *β*, *σ* that maximize the probability of observing the data under our calibration model, and find good predictive performance in both training and leave-one-out held out data (**Fig. S10B,C**). Moreover, the vast majority of the data lies within the range of binding energies spanned by the predicted for the experimentally validated data, suggesting that we can effectively interpolate the normalized MIC scores for nearly the full Im/E sequence space (**Fig. S10D**). Finally, in order to have predicted normalized MIC scores in the same range as the empirical path, scores predicted to be higher than 6 were truncated to 6.

### Fitness landscape visualization

Visualization method as previously described^43^. Briefly, we construct a model of molecular evolution where a population evolves via single amino acid substitutions and the rate at which each possible substitution becomes fixed in the population reflects its selective advantage or disadvantage relative to the currently fixed sequence. More specifically, in our model the rate of evolution from sequence *i* to any mutationally adjacent sequence *j* is given by

$$Q_{ij}=\frac{S_{ij}}{1-e^{S_{ij}}}$$

where *S*_*ij*_ is the scaled selection coefficient (population size times the selection coefficient of *j* relative to *i*), time is measured relative to the amino acid mutation rate (each possible amino acid mutation occurs at rate 1), and the total leaving rate from each sequence *i* is given by

$$Q_{ii}=-\sum_{j\neq i}Q_{ij}$$

In the current context, *S*_*ij*_ = *c*(*f*_*j*_ − *f*_*i*_), where *f*_*i*_ is the predicted normalized MIC score at sequence *i*. For this analysis we choose *c* so that the equilibrium expected viability score is 3, corresponding to a roughly fivefold increase in the expected normalized MIC score relative to neutrality, which would produce an expected normalized MIC score of 0.65. Considering genotypes with normalized MIC scores larger than 3, this strength of selection corresponds to an over tenfold increase in the time spent in those genotypes with respect to neutral evolution (from 4.61 to 52.84% of the time).

Given the rate matrix *Q* for our evolutionary model, we then construct the visualization by using the subdominant right eigenvectors associated with the smallest magnitude non-zero eigenvalues of this rate matrix as coordinates for the low dimensional representation of the landscape, where each such coordinate defines one of the “diffusion axes” used in the visualization. This produces a visualization that reflects the long-term barriers to diffusion in sequence space, and, in particular, clusters of sequences in the visualization correspond to sets of initial states from which the evolutionary model approaches its stationary distribution in the same manner, and multi-peaked fitness landscapes appear as broadly separated clusters with one peak in each cluster. Moreover, by scaling the axes appropriately, as is done here, these axes can be given units of $\sqrt{\text{ time }}$ and it can be shown that the resulting distances reflect evolutionary times under this model. In particular, using these coordinates the squared Euclidean distance between arbitrary sequences *i* and *j* optimally approximates (in a specific sense) the sum of the expected time to evolve from *i* to *j* and the expected time to evolve from *j* to *i*. See ref.^43^ for details.

## References

[1] Poelwijk F. J., Kiviet D. J., Weinreich D. M. & Tans S. J. Empirical fitness landscapes reveal accessible evolutionary paths. Nature 445, 383–386 (2007). 17251971 10.1038/nature05451

[2] Aakre C. D. Evolving new protein-protein interaction specificity through promiscuous intermediates. Cell 163, 594–606 (2015). 26478181 10.1016/j.cell.2015.09.055 PMC4623991

[3] Levin K. B. Following evolutionary paths to protein-protein interactions with high affinity and selectivity. Nat. Struct. Mol. Biol. 16, 1049–1055 (2009). 19749752 10.1038/nsmb.1670

[4] Siddiq M. A., Hochberg G. K. & Thornton J. W. Evolution of protein specificity: insights from ancestral protein reconstruction. Curr. Opin. Struct. Biol. 47, 113–122 (2017). 28841430 10.1016/j.sbi.2017.07.003 PMC6141201

[5] McClune C. J. & Laub M. T. Constraints on the expansion of paralogous protein families. Curr. Biol. 30, R460–R464 (2020). 32428482 10.1016/j.cub.2020.02.075

[6] Meenan N. A. G. The structural and energetic basis for high selectivity in a high-affinity protein-protein interaction. Proc. Natl. Acad. Sci. U. S. A. 107, 10080–10085 (2010). 20479265 10.1073/pnas.0910756107 PMC2890441

[7] Papadakos G., Wojdyla J. A. & Kleanthous C. Nuclease colicins and their immunity proteins. Q. Rev. Biophys. 45, 57–103 (2012). 22085441 10.1017/S0033583511000114

[8] Kuriyan J., Konforti B. & Wemmer D. The molecules of life: Physical and chemical principles. (Garland Science, 2012).

[9] Mohammadi M., Olsen S. K. & Ibrahimi O. A. Structural basis for fibroblast growth factor receptor activation. Cytokine Growth Factor Rev. 16, 107–137 (2005). 15863029 10.1016/j.cytogfr.2005.01.008

[10] Itoh N. & Ornitz D. M. Evolution of the Fgf and Fgfr gene families. Trends Genet. 20, 563–569 (2004). 15475116 10.1016/j.tig.2004.08.007

[11] Starr T. N. & Thornton J. W. Epistasis in protein evolution. Protein Sci. 25, 1204–1218 (2016). 26833806 10.1002/pro.2897 PMC4918427

[12] Podgornaia A. I. & Laub M. T. Protein evolution. Pervasive degeneracy and epistasis in a protein-protein interface. Science 347, 673–677 (2015). 25657251 10.1126/science.1257360

[13] Bridgham J. T., Carroll S. M. & Thornton J. W. Evolution of hormone-receptor complexity by molecular exploitation. Science 312, 97–101 (2006). 37000978 10.1681/01.asn.0000926836.46869.e5

[14] Welin M. & Nordlund P. Understanding specificity in metabolic pathways—Structural biology of human nucleotide metabolism. Biochem. Biophys. Res. Commun. 396, 157–163 (2010). 20494131 10.1016/j.bbrc.2010.04.054

[15] Starr T. N., Picton L. K. & Thornton J. W. Alternative evolutionary histories in the sequence space of an ancient protein. Nature 549, 409–413 (2017). 28902834 10.1038/nature23902 PMC6214350

[16] McClune C. J., Alvarez-Buylla A., Voigt C. A. & Laub M. T. Engineering orthogonal signalling pathways reveals the sparse occupancy of sequence space. Nature 574, 702–706 (2019). 31645757 10.1038/s41586-019-1639-8 PMC6858568

[17] Poelwijk F. J., Socolich M. & Ranganathan R. Learning the pattern of epistasis linking genotype and phenotype in a protein. Nature Communications vol. 10 Preprint at 10.1038/s41467-019-12130-8 (2019). PMC6746860 31527666

[18] Sarkisyan K. S. Local fitness landscape of the green fluorescent protein. Nature 533, 397–401 (2016). 27193686 10.1038/nature17995 PMC4968632

[19] Weinreich D. M., Delaney N. F., DePristo M. A. & Hartl D. L. Darwinian evolution can follow only very few mutational paths to fitter proteins. Science 312, 111–114 (2006). 16601193 10.1126/science.1123539

[20] Ding D. Co-evolution of interacting proteins through non-contacting and non-specific mutations. Nat Ecol Evol 6, 590–603 (2022). 35361892 10.1038/s41559-022-01688-0 PMC9090974

[21] Nocedal I. & Laub M. T. Ancestral reconstruction of duplicated signaling proteins reveals the evolution of signaling specificity. Elife 11, (2022). 10.7554/eLife.77346 PMC9208753 35686729

[22] Hochberg G. K. A. & Thornton J. W. Reconstructing Ancient Proteins to Understand the Causes of Structure and Function. Annu. Rev. Biophys. 46, 247–269 (2017). 28301769 10.1146/annurev-biophys-070816-033631 PMC6141191

[23] Gong L. I., Suchard M. A. & Bloom J. D. Stability-mediated epistasis constrains the evolution of an influenza protein. Elife 2, e00631 (2013). 23682315 10.7554/eLife.00631 PMC3654441

[24] Harms M. J. & Thornton J. W. Evolutionary biochemistry: revealing the historical and physical causes of protein properties. Nat. Rev. Genet. 14, 559–571 (2013). 23864121 10.1038/nrg3540 PMC4418793

[25] Dean A. M. & Thornton J. W. Mechanistic approaches to the study of evolution: the functional synthesis. Nat. Rev. Genet. 8, 675–688 (2007). 17703238 10.1038/nrg2160 PMC2488205

[26] Cramer W. A., Sharma O. & Zakharov S. D. On mechanisms of colicin import: the outer membrane quandary. Biochem. J 475, 3903–3915 (2018). 30541793 10.1042/BCJ20180477

[27] Li W. Highly Discriminating Protein–Protein Interaction Specificities in the Context of a Conserved Binding Energy Hotspot. J. Mol. Biol. 337, 743–759 (2004). 15019791 10.1016/j.jmb.2004.02.005

[28] Keeble A. H., Kirkpatrick N., Shimizu S. & Kleanthous C. Calorimetric dissection of colicin DNase--immunity protein complex specificity. Biochemistry 45, 3243–3254 (2006). 16519519 10.1021/bi052373o

[29] Kirkup B. C. & Riley M. A. Antibiotic-mediated antagonism leads to a bacterial game of rock-paper-scissors in vivo. Nature 428, 412–414 (2004). 15042087 10.1038/nature02429

[30] Kortemme T. Computational redesign of protein-protein interaction specificity. Nat. Struct. Mol. Biol. 11, 371–379 (2004). 15034550 10.1038/nsmb749

[31] Netzer R. Ultrahigh specificity in a network of computationally designed protein-interaction pairs. Nat. Commun. 9, 5286 (2018). 30538236 10.1038/s41467-018-07722-9 PMC6290019

[32] Warszawski S., Netzer R., Tawfik D. S. & Fleishman S. J. A ‘fuzzy’-logic language for encoding multiple physical traits in biomolecules. J. Mol. Biol. 426, 4125–4138 (2014). 25311857 10.1016/j.jmb.2014.10.002 PMC4270444

[33] Keeble A. H. Experimental and computational analyses of the energetic basis for dual recognition of immunity proteins by colicin endonucleases. J. Mol. Biol. 379, 745–759 (2008). 18471830 10.1016/j.jmb.2008.03.055

[34] Wojdyla J. A., Fleishman S. J., Baker D. & Kleanthous C. Structure of the Ultra-High-Affinity Colicin E2 DNase-Im2 Complex. J. Mol. Biol. (2012) doi:10.1016/j.jmb.2012.01.019. 22306467

[35] Kosloff M., Travis A. M., Bosch D. E., Siderovski D. P. & Arshavsky V. Y. Integrating energy calculations with functional assays to decipher the specificity of G protein-RGS protein interactions. Nat. Struct. Mol. Biol. 18, 846–853 (2011). 21685921 10.1038/nsmb.2068 PMC3130846

[36] Sharp C., Bray J., Housden N. G., Maiden M. C. J. & Kleanthous C. Diversity and distribution of nuclease bacteriocins in bacterial genomes revealed using Hidden Markov Models. PLoS Comput. Biol. 13, e1005652 (2017). 28715501 10.1371/journal.pcbi.1005652 PMC5536347

[37] Alford R. F. The Rosetta All-Atom Energy Function for Macromolecular Modeling and Design. J. Chem. Theory Comput. 13, 3031–3048 (2017). 28430426 10.1021/acs.jctc.7b00125 PMC5717763

[38] Bang-Jensen J. & Gutin G. Z. Digraphs: Theory, Algorithms and Applications. (Springer, 2008).

[39] O’Meara M. J. A Combined Covalent-Electrostatic Model of Hydrogen Bonding Improves Structure Prediction with Rosetta. J. Chem. Theory Comput. 11, 609–622 (2015). 25866491 10.1021/ct500864r PMC4390092

[40] Kondrashov D. A. & Kondrashov F. A. Topological features of rugged fitness landscapes in sequence space. Trends Genet. 31, 24–33 (2015). 25438718 10.1016/j.tig.2014.09.009

[41] Park H. Simultaneous Optimization of Biomolecular Energy Functions on Features from Small Molecules and Macromolecules. J. Chem. Theory Comput. 12, 6201–6212 (2016). 27766851 10.1021/acs.jctc.6b00819 PMC5515585

[42] Ortlund E. A., Bridgham J. T., Redinbo M. R. & Thornton J. W. Crystal structure of an ancient protein: evolution by conformational epistasis. Science 317, 1544–1548 (2007). 17702911 10.1126/science.1142819 PMC2519897

[43] McCandlish D. M. Visualizing fitness landscapes. Evolution 65, 1544–1558 (2011). 21644947 10.1111/j.1558-5646.2011.01236.x PMC3668694

[44] Khersonsky O. Automated Design of Efficient and Functionally Diverse Enzyme Repertoires. Mol. Cell 72, 178–186.e5 (2018). 30270109 10.1016/j.molcel.2018.08.033 PMC6193528

[45] Fleishman S. J., Khare S. D., Koga N. & Baker D. Restricted sidechain plasticity in the structures of native proteins and complexes. Protein Sci. 20, 753–757 (2011). 21432939 10.1002/pro.604 PMC3081553

[46] Unger T., Jacobovitch Y., Dantes A., Bernheim R. & Peleg Y. Applications of the Restriction Free (RF) cloning procedure for molecular manipulations and protein expression. J. Struct. Biol. 172, 34–44 (2010). 20600952 10.1016/j.jsb.2010.06.016

[47] Erijman A., Dantes A., Bernheim R., Shifman J. M. & Peleg Y. Transfer-PCR (TPCR): a highway for DNA cloning and protein engineering. J. Struct. Biol. 175, 171–177 (2011). 21515384 10.1016/j.jsb.2011.04.005

[48] Wallis R. In vivo and in vitro characterization of overproduced colicin E9 immunity protein. Eur. J. Biochem. 207, 687–695 (1992). 1633820 10.1111/j.1432-1033.1992.tb17096.x

[49] Wallis R. Protein-Protein Interactions in Colicin E9 DNase-Immunity Protein Complexes. 2. Cognate and Noncognate Interactions That Span the Millilmolar to Femptomolar Affinity Range. Biochemistry vol. 34 13751–13759 Preprint at 10.1021/bi00042a005 (1995). 7577967

[50] Li W. & Godzik A. Cd-hit: A fast program for clustering and comparing large sets of protein or nucleotide sequences. Bioinformatics 22, 1658–1659 (2006). 16731699 10.1093/bioinformatics/btl158

[51] Edgar R. C. MUSCLE: a multiple sequence alignment method with reduced time and space complexity. BMC Bioinformatics 5, 113 (2004). 15318951 10.1186/1471-2105-5-113 PMC517706

[52] Lite T.-L. V. Uncovering the basis of protein-protein interaction specificity with a combinatorially complete library. Elife 9, (2020). 10.7554/eLife.60924 PMC7669267 33107822

[53] Crona K. Rank orders and signed interactions in evolutionary biology. Elife 9, (2020). 10.7554/eLife.51004 PMC7000213 31934856

[54] Conrad M. The geometry of evolution. Biosystems. 24, 61–81 (1990). 2224072 10.1016/0303-2647(90)90030-5

