# Evolutionary paths that link orthogonal pairs of binding proteins

**DOI:** 10.21203/rs.3.rs-2836905/v2

**Published:** 2023-12-13

**Authors:** Ziv Avizemer, Carlos Martí-Gómez, Shlomo Yakir Hoch, David M. McCandlish, Sarel J. Fleishman

**Affiliations:** 1 Department of Biomolecular Sciences, Weizmann Institute of Science, 7610001, Rehovot, Israel; 2 Simons Center for Quantitative Biology, Cold Spring Harbor Laboratory, Cold Spring Harbor, NY 11724

**Keywords:** Epistasis, evolution, protein design, Rosetta, protein-protein interactions, orthogonality, tness landscape

## Abstract

Some protein binding pairs exhibit extreme specificities that functionally insulate them from homologs. Such pairs evolve mostly by accumulating single-point mutations, and mutants are selected if their affinity exceeds the threshold required for function^1–4^. Thus, homologous and high-specificity binding pairs bring to light an evolutionary conundrum: how does a new specificity evolve while maintaining the required affinity in each intermediate^5,6^? Until now, a fully functional single-mutation path that connects two orthogonal pairs has only been described where the pairs were mutationally close thus enabling experimental enumeration of all intermediates^2^. We present an atomistic and graph-theoretical framework for discovering low molecular strain single-mutation paths that connect two extant pairs, enabling enumeration beyond experimental capability. We apply it to two orthogonal bacterial colicin endonuclease-immunity pairs separated by 17 interface mutations^7^. We were not able to find a strain-free and functional path in the sequence space defined by the two extant pairs. But including mutations that bridge amino acids that cannot be exchanged through single-nucleotide mutations led us to a strain-free 19-mutation trajectory that is completely viable *in vivo*. Our experiments show that the specificity switch is remarkably abrupt, resulting from only one radical mutation on each partner. Furthermore, each of the critical specificity-switch mutations increases fitness, demonstrating that functional divergence could be driven by positive Darwinian selection. These results reveal how even radical functional changes in an epistatic fitness landscape may evolve.

Cellular signaling and metabolism rely on high interaction specificity^8^. Achieving high specificity may appear particularly challenging among pairs of proteins that exhibit sequence and structural similarity. Yet, such homologous pairs are, in fact, often functionally isolated, thereby establishing multiple orthogonal interactions^7,9,10^. The challenge to understanding how orthogonality is achieved among homologous pairs is exacerbated when considering that the evolution of a functionally orthogonal pair requires changes that destabilize interactions between the derived pair and its ancestor. To avoid being purged by evolutionary selection, however, the changes that destabilize the interaction with the ancestral partners must accumulate in an order that maintains binding between the two members of the derived pair following every change^11^. How the proteins in a pair coevolve to exhibit high affinity in each intermediate while disabling their interactions with the ancestral proteins remains a key question in understanding diversification of gene families and protein interaction networks^12–14^.

Some commonly used approaches to address questions on the evolution of binding pairs use ancestral-sequence reconstruction, laboratory evolution, and deep mutational scanning^2,3,12,15–21^. These strategies provided important insights on some of the constraints that shape evolutionary trajectories that lead to new or improved activities^12,22,23^. To date, however, reconstruction of a single-point mutation path among orthogonal pairs has been challenging and accomplished only when the number of mutations between the pairs was small enough to allow complete experimental enumeration of all intermediate mutants^2^. Thus, understanding how multiple incompatible interactions are exchanged between binding pairs is elusive, and fundamental questions in molecular evolution of orthogonal pairs have remained understudied^11,24,25^; among them: Do mutations on one side of the interface enable mutations on the other? Do evolutionary intermediates present dual-specificities or are the intermediates specific as observed in the extant pairs? Do specificity switches occur gradually through the accumulation of many mutations or abruptly through a handful? And how, despite the need to accumulate destabilizing mutations, are inactive intermediates avoided?

We focused on bacterial colicin endonuclease/immunity pairs^7^. The colicins are SOS-induced nonspecific DNA endonuclease toxins (ColE) that coexpress with cognate high-affinity immunity proteins (Im) that protect the producing cell from degradation of its genomic DNA. Following secretion of this complex from the producing cell, the ColE receptor-binding domain attaches to the outer-membrane target receptor of a neighboring cell. Next, the cytotoxic endonuclease domain (E) is unfolded, dissociating from the protective Im protein, and is then translocated to the host cytoplasm. In the host cytoplasm, the E refolds and degrades DNA, killing the cell unless the host harbors a cognate and protective Im^7,26^. Cognate E/Im pairs exhibit ultrahigh binding affinity (K_D_ ≤ 10^−14^ M) and Im proteins that bind E at K_D_ > 10^−10^ M are not protective^27^. During evolution, the colicins have diversified to four homologous pairs that exhibit ultrahigh specificities of 5–10 orders of magnitude with respect to one another^6,27,28^. Thus, the four Im proteins protect the producing cell against the toxicity of their cognate E proteins but provide no protection against the homologous noncognate E proteins. This system thereby presents a clear example of functional orthogonality under strong evolutionary selection. Due to their remarkably high affinities and specificities, the colicin E/Im pairs have served as model systems to study evolutionary dynamics^3,29^ and biomolecular recognition in protein engineering and design^6,30–35^. Insights from these studies and the straightforward approach to test the evolutionary fitness of pairs *in vivo* within their native host attracted us to use this system to study the coevolution of orthogonal binding pairs. Because only four E/Im pairs are known^7,36^, however, ancestral sequences cannot be reliably reconstructed as in other systems^4,13,21,22^. Here, we develop an atomistic strategy that models the stability of each possible intermediate in the complete combinatorial binding landscape and searches for a single-mutation path between the extant pairs while minimizing molecular strain that may arise from protein instability or incompatible interactions between the partners. This framework allows testing different mechanistic hypotheses on the sequence and physical constraints that shape the fitness landscape.

## Results

### Orthogonality and structural incompatibilities

**Figure 1.**
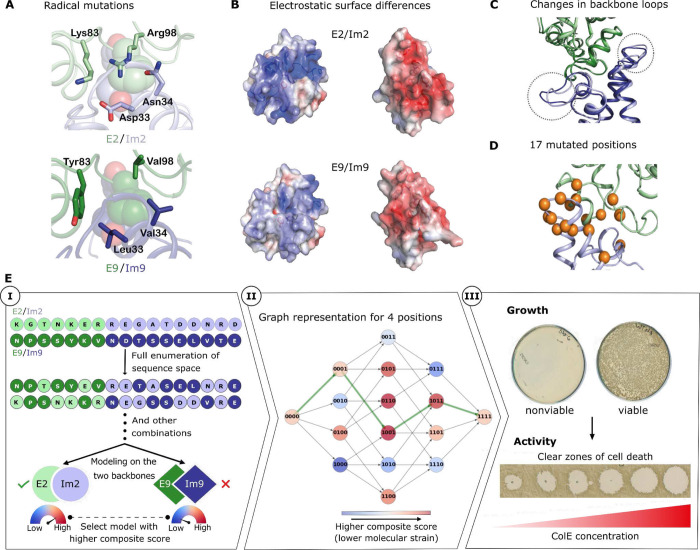
The structural basis for orthogonal binding specificities in colicin E2/Im2 and E9/Im9 and an energy-based strategy to discover evolutionarily plausible paths. (A) Four radical mutations between E2/Im2 and E9/Im9 (PDB entries: 3u43 and 1emv, respectively). The conserved hotspot residues, E:Phe86 and Im:Tyr54-Tyr55, are shown in spheres in all molecular representations. (B) Open-book representation of the two complexes with solvent-accessible surfaces colored according to electrostatic representation. (C) Aligned structures of E2/Im2 and E9/Im9. Two loops that exhibit backbone conformation differences are marked in dashed circles. (D) 17 interfacial positions that differ between E2/Im2 and E9/Im9 are represented as orange spheres on the structure of E2/Im2. (E) (**Step I**) Starting from the two wild type pairs, all possible sequences are modeled on both structures. For each sequence, the lowest-strain (best score) backbone is selected. (**II**) An exemplary subgraph for four positions. Mutants (represented by nodes) are connected if they differ by one mutation. ‘0’ and ‘1’ represent the start and end amino acid identities. The minimum-strain path between the terminal nodes is marked in green. (**III**) Two-step experimental validation of the calculated minimum-strain path. First, we test the growth of bacteria carrying a plasmid encoding a mutant pair (Fig. S1). Second, we purify the ColE/Im complex and apply it in tenfold serial dilutions to naive JM83 *E. coli* cells. Clear zones of cell death indicate ColE toxicity.

We used the two most closely related E/Im pairs, known as pairs 2 and 9, as the subjects of our study. In these pairs, the interaction hotspot region, comprising E:Phe86 and Im:Tyr54-Tyr55 (Fig. 1A, spheres), is responsible for a large portion of the binding energy and is conserved in sequence and conformation (amino acid numbering according to E2/Im2^34^). Conversely, other interfacial positions contribute less to affinity but determine binding specificity^27,35^ and are therefore the focus of our analysis. Some of these positions exhibit dramatic changes in their physicochemical properties. For instance, in E9/Im9, interfacial positions surrounding the hotspot include Val98, Tyr83 (E9) and Leu33 and Val34 (Im9) compared respectively to Arg, Lys, Asp, and Asn in E2/Im2 (Fig. 1A). The overall effect of these four radical mutations is a striking change from a polar and charged interface in E2/Im2 that is mediated by a buried interfacial electrostatic interaction between E:Arg98 and Im:Asp33 to a largely hydrophobic interaction in E9/Im9 (Fig. 1A & B). Additionally, the Im protein backbone differs in two unstructured regions connecting helices 1 and 2 and helices 3 and 4 (Fig. 1C), both containing critical specificity-determining positions^27,34^. Thus, despite the high sequence and structure similarity between the two pairs, their binding interfaces feature mutually incompatible networks of molecular interactions (hydrophobic versus polar/charged) that underlie the ultrahigh specificity barrier between them.

### *In vivo* validation of a single-mutation path

We develop an atomistic modeling framework to discover coevolutionarily plausible (single-amino acid change) paths that connect two extant binding pairs through functional intermediates. The interfaces of E2/Im2 and E9/Im9 differ by 17 interfacial positions (7 and 10 positions on E and Im, respectively) (Fig. 1D and Table S1). The sequence space that encompasses both wild type pairs comprises all combinations of mutations at these interfacial positions, for a total of 2^17^ (approximately 131,000) possible mutants. In addition to the 17 interfacial mutations, the two colicin pairs differ by 33 mutations outside the interface. To establish a common genetic background for assessing the evolutionary intermediates, we tested a variant of E9/Im9 that encodes all of the E2/Im2 mutations outside the binding interface (termed E9/Im9_chimera_) (Table S2). We confirmed that E9/Im9_chimera_ was as viable as E9/Im9 (Fig. S4) and that its endonuclease toxicity remained equivalent to that of the two wild type pairs (Fig. S5). Additionally, Im9_chimera_ maintained and even exhibited somewhat improved neutralization of E9 and E9_chimera_ relative to Im9 (Fig. S4). We thus used wild type E2/Im2 and E9/Im9_chimera_ as the genetic backgrounds for testing intermediates, allowing us to focus only on the interfacial mutations. For design calculations, however, we maintained the wild type identities in all positions outside the interface to minimize the degree of extrapolation from the experimentally determined structures.

**Figure 2.**
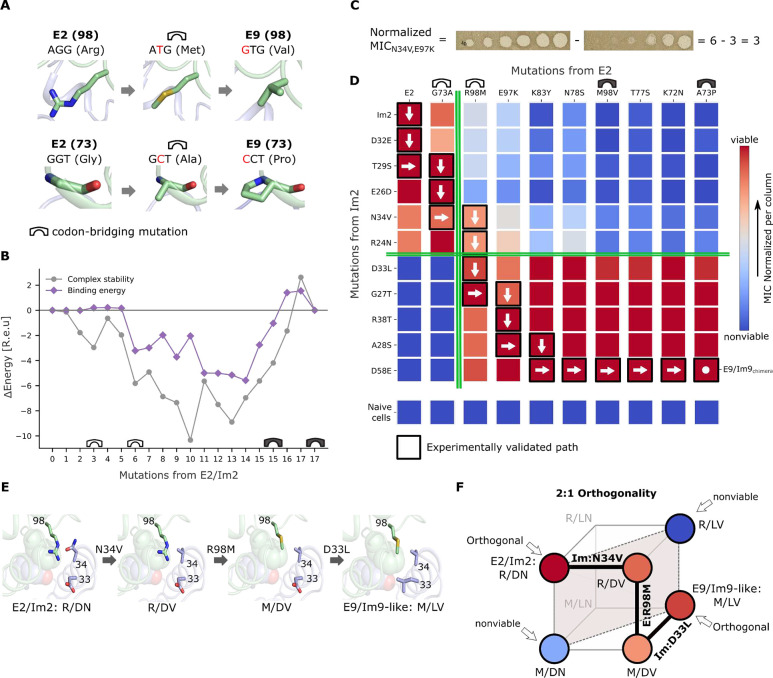
Experimental validation of an evolutionarily plausible path linking two orthogonal binding pairs. (A) Examples of codon-bridging mutations. DNA nucleotide mutations are indicated in red. (B) Binding and stability energy differences of the intermediates in the minimum-strain path relative to the two wild type pairs. An empty bridge icon represents the introduction of a bridging mutation, and a full bridge icon represents its resolution. (C) An example of the inferred normalized minimal inhibitory concentration (MIC). (D) Functional specificity map. Each endonuclease on the viable path was applied to bacteria harboring each immunity protein. Mutations from E2 accumulate from left-to-right and mutations from Im2 accumulate from top-to-bottom. A vertical green line marks the specificity switch on E and a horizontal one marks the specificity switch on Im. The mutations are completely reversible, and arrows are only visual guides. The MIC scores within the map are normalized to the minimal and maximal killing (MIC on naive cells) of each E (in each column), and higher viability corresponds to higher normalized MIC. The scores within the map are an average of two technical repeats (raw averages and standard deviations in Table S5). (E) Molecular models of the key mutations producing the orthogonality along the experimentally validated evolutionary path consistent with the model in panel F. The hotspot is represented in spheres. (F) General model of orthogonality in a bimolecular fitness landscape involving two sites in one molecule (Im protein) and one site in the other molecule (E toxin) defining what we call a 2:1 orthogonality model (see Supplementary Information and Fig. S9). The corners of the cube represent all the possible pairs of sequences, and colors are consistent with panel D. Thick lines represent the empirical path and dotted lines join the pairs whose fitness determines orthogonality of the pairs for interacting molecules.

Because simultaneous multinucleotide mutations are less likely to occur in evolution compared to single-nucleotide mutations^37^, we checked whether some of the 17 interface mutations required several nucleotide exchanges. Indeed, at seven interfacial positions the amino acids cannot be exchanged by single-nucleotide mutations, suggesting that unseen amino acid intermediates may have been necessary to bridge the two extant identities (codon bridges). We analyzed homologs of colicin pairs 2 and 9 to find indications for possible bridging identities that may have been used by evolution but found almost no interface mutations in the natural diversity (Table S3 and S4). Because the sequence space that includes all possible codon bridges is too large for atomistic modeling (>10^8^ intermediates), we selected at each position one bridging mutation that was physicochemically intermediate between the two extant identities (Table S1); for example, in position E:98, we enlarged the sequence space by adding Met to the two extant identities, Arg and Val, and in E:73, we added Ala to the extant identities, Gly and Pro (Fig. 2A).

In an evolutionarily plausible path, each intermediate must exhibit low molecular strain to be functional and confer viability on the producing cell. Therefore, we score each mutant according to its system and binding energies using the Rosetta all-atom energy function^38^. The energies are then transformed using a logistic function into values between 0 and 1 that approximate the likelihood that the proteins are stable and exhibit high affinity, and these values are combined into a composite score through a “fuzzy”-logic AND gate^32^ that estimates whether both energies are sufficiently favorable. To partly address the possibility that mutations deform the backbone, we model each mutant in the context of the two wild type structures (which differ mainly in two interfacial Im loops and in a modest rigid-body reorientation^34^; Fig. 1C) and choose the best-scoring model to represent each mutant (Fig. 1E, step I). Finally, to represent all mutational paths that traverse the sequence landscape, we construct a directed graph in which the nodes represent all mutants, and edges connect nodes that are separated by a single amino acid mutation. We also add edges through the codon bridges to connect the seven amino acids that cannot be exchanged by single-nucleotide mutations. The paths are not forced to go through the codon-bridging identities, however. Instead, edges go both through the bridges and circumventing them, thus ensuring that bridges are selected only if they contribute to lowering molecular strain. The graph edges are weighted according to the negative logarithm of the composite score of the predecessor node (Fig. 1E, step II). Thus, any path that connects the terminal nodes representing the extant pairs is a single-mutation trajectory by construction, and the sum of edge weights represents molecular strain along the path (lower weights correspond to lower strain). We then use a shortest-path algorithm^39^ to find minimum-strain paths (see Methods). We also computed a path without including bridges to serve as a reference (Fig. S2) and three additional paths without bridges that used different parameter choices to construct the graph (Fig. S3). We found that the energy profile of the optimal path with codon-bridging mutations was substantially lower (more favorable) than those of any of the other four paths that excluded codon bridges (Fig. 2B, S2, and S3). Moreover, throughout most of this trajectory, the computed energies were lower than those of the wild type pairs, rising slightly above them in the few final mutants that adopt the backbone conformation of E9/Im9 (Fig. 2B).

We next tested all paths by experimental viability screening in *E. coli* to probe the ability of bacteria to produce the high-affinity pairs and to verify that the E proteins maintained their toxicity towards naive cells (that do not harbor any immunity) (Fig. 1E, III). Remarkably, experiments revealed that all 18 intermediates from the optimal path with bridging mutations were viable (Fig. S1), and that all endonuclease intermediates retained toxicity towards naive JM83 cells (Fig. S6). By contrast, the four alternative paths were not feasible, and each exhibited at least seven intermediates that were non-viable (Fig. S2 and S3). We concluded that the minimum-strain path with the bridging mutations is an evolutionarily plausible trajectory linking the two wild type orthogonal colicin pairs through fully functional single-point amino acid mutations. Because our computational predictions are subject to uncertainty and error, we cannot rule out that viable paths exist within the sequence space that excludes bridges. Nonetheless, the fact that the atomistic calculations failed to identify a low-strain path in this sequence space and the predicted best candidate paths all contained inviable intermediates suggests that the bridges are indeed necessary to reduce the molecular strain of introducing mutually incompatible specificity-switching mutations.

### An abrupt specificity switch

To investigate whether the specificity switch between the two colicin pairs occurs gradually through many small-effect mutations or abruptly through a few strong-effect mutations, we built a functional quantitative specificity map between E and Im variants along the viable path. We applied the eight intermediate ColE mutants, the wild type ColE2 and ColE9_chimera_ in tenfold serial dilutions to JM83 cells transformed with the nine Im intermediates, wild type Im2, and Im9_chimera_. We then visually estimated how protective each Im protein was with respect to each ColE according to the minimal inhibitory concentration (MIC; the tenfold dilution of ColE in which no clear zones were observed; Fig. 2C) and integrated the results into a functional specificity map (Fig. 2D). We also verified that all purified ColE/Im complexes exhibited similar concentrations (approx. 3 mg/ml) (Fig. S7) and that Im intermediates were similarly expressed (approx. 2 mg/ml) and stable up to 50 °C (Fig. S8).

Strikingly, the map reveals that orthogonality is preserved along most of the steps in the evolutionary path, and that as few as three substitutions are sufficient to induce a complete switch in specificity. Two of these substitutions take place at consecutive positions in the immunity protein (Asn34Val and Asp33Leu) and one in the E protein (Arg98Met). Im:Asn34Val abrogates a hydrogen bond to E:Arg98 and acts as an enabling mutation, allowing the mutated Im to bind the E:Arg98Met mutant (Fig. 2E). E:Arg98Met completely switches the specificity pattern of the endonuclease, interacting strongly with Im9-like variants but substantially less with the Im2-like variants (Fig. 2D). Subsequently, Im:Asp33Leu, a known specificity determinant^27,34^, eliminates the Im protein recognition of E2-like endonucleases in favor of E9-like variants and completes the functional transition (Fig. 2D).

These results offer several insights that may generalize to other coevolving systems. First, the dramatic specificity switch occurs in only a few evolutionary steps — similar to observations in several molecular systems, though the path reconstructed here is much longer than any that have been previously characterized^4,21,25^. Second, the results also demonstrate how two incompatible molecular interaction networks (charged/polar *vs.* hydrophobic) could be exchanged one mutation at a time. Third, in Supplemental Information, we study general molecular mechanisms that produce orthogonal binders, concluding that they can be classified by the minimal number of mutations that are required for the emergence of orthogonality (Fig. S9). The mechanism of orthogonality in the experimentally validated path is what we call “2:1 orthogonality” (Fig. 2F). In this model, orthogonality necessarily evolves through a strict order of mutations starting with a mutation on one partner, then the other, and finally a mutation in the first partner that interacts with the first mutation, as observed in the empirical path (Im:Asn34Val→E:Arg98Met→Im:Asp33Leu) (Fig. 2E and 2F). Consistent with this proposed mechanism, the two interacting sites in the same molecule (Im:33 and Im:34) form an epistatic interaction with the same site (E:98) and are indeed spatially close (Fig. 2E).

The specificity switch, however, is more complex when viewed relative to the four E mutants that contain the Met98 codon-bridging mutation. Following the E:Arg98Met mutation and until the E:Met98Val mutation (E9 identity), the endonucleases present a reduced specificity barrier for the Im proteins, though they maintain nearly equal toxicity when applied to naive JM83 cells (Fig. S6). Thus, although the Im intermediates close to Im2 are less protective than those close to Im9 against endonucleases that carry Met98, the former nonetheless present a significant level of protection (Fig. 2D). The Met98-containing endonucleases are, therefore, multispecific relative to the very strict functional orthogonality observed in all the other endonucleases. E:Arg98Met is thus not only a specificity watershed but may also be an enabling mutation, lowering the specificity barrier and allowing the accumulation of additional E and Im mutations. Once the Val mutation is fixed, the specificity barrier is, once again, extremely strict.

The case presented in this evolutionary trajectory is one in which a minimal number of large-effect mutations (two) establishes a specificity watershed; yet, many surrounding mutations are needed to stabilize and enable the pair of mutations^40^.

### Stepwise resolution of epistatic interactions

The empirical specificity map shows that many mutations could have appeared in a different order. For example the map suggests that Im:Arg24Asn preceding rather than following E:Arg98Met might have produced an even higher-fitness path than the computed one. In fact, we can determine that >3,500 alternative and fully functional paths traverse the viable regions in the empirical map (red regions in Fig. 2D) by counting the number of viable paths within the set of empirically measured genotypes (see Methods). The existence of such alternatives raises questions about the possible ordering of mutations that cannot be answered on the basis of the empirical specificity map alone. We therefore used the empirical data from the specificity map (including cognate and noncognate pairs from Fig. 2D) to calibrate a predictor of normalized MIC scores (Fig. 2C) based on the Rosetta binding energy of each pair (Pearson r=0.92, *p*-value<10^−16^ in leave-one-out cross-validation; Fig. S10; see Methods). We then predicted normalized MIC scores for all 2.2 million possible E/Im pairs (including all codon bridges) and applied a technique for visualizing fitness landscapes^41^ to produce a low-dimensional representation in which distances between pairs approximate the time it may take for a population to evolve from one pair to another under selection for high normalized MIC score (see Methods).

**Figure 3.**
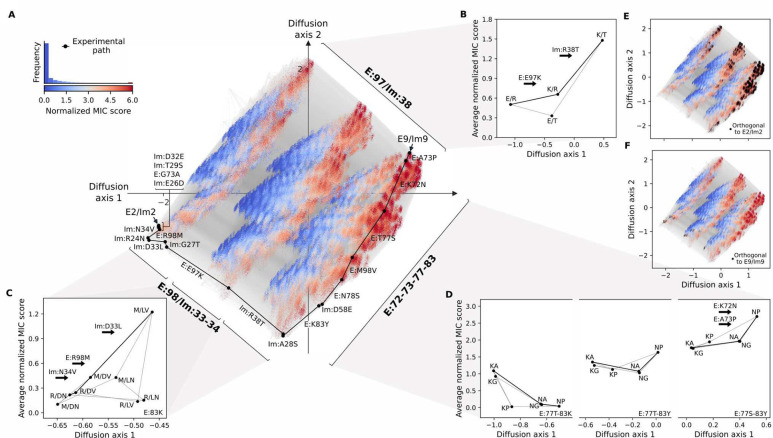
Computed global fitness landscape highlights the potential role of multiple epistatic interactions in effecting the specificity switch. (A) Visualization of the predicted fitness landscape. Distance between points represents the time to evolve between E/Im pairs under selection for high normalized MIC scores (see Methods). Nodes represent protein pairs and edges connect pairs that differ by a single amino acid substitution. Node color indicates normalized MIC score. Sequences with higher scores are plotted close to the viewer above to emphasize the relationships among functional sequences. Empirical path is highlighted in black. Inset shows the distribution of predicted normalized MIC scores across all possible pairs together with the color scale. Interactions between key mutations are marked in the visualization and simplified in panels B-D depicting the networks of average MIC scores defined by the identities at the key sets of interacting positions. (B,C) Diagrams representing the average MIC score and average position at Diffusion axis 1 for each possible subsequence at positions E:97/Im:38 (B) and E:98/Im:33–34 in the E:Lys83 context (C). The empirical trajectory across each set of sites is highlighted by a thick line. (D) Diagrams representing the average MIC score and average position at Diffusion axis 1 for each possible subsequence at positions E:72–73 in the E:Lys83-Thr77 context on the left, E:Tyr83-Thr77 on the center, and in the E:Tyr83-Ser77 context on the right. (E,F) Visualization of the fitness landscape with overlaid black dots indicating pairs that are predicted to be orthogonal to pair E2/Im2 (E) and E9/Im9 (F). Pairs were defined to be orthogonal to either E2/Im2 or E9/Im9 if their predicted normalized MIC score was at least as high as that of the alternative extant pair minus 0.5, and if their combinations with E2 and Im2 or E9 and Im9, respectively, were at most 0.5 greater than the minimum of E2/Im9 and E9/Im2.

The visualization reveals that the predicted fitness landscape is shaped by three main sets of molecular interactions that separate sequences along the first two “Diffusion axes” (see methods) that define the low-dimensional representation (Fig. 3A). The first interaction separates the fitness landscape into three main groups of sequences due to an incompatibility between mutations at positions E:97 and Im:38. In E2/Im2, these positions (E2:Glu97/Im2:Arg38) form a saltbridge across the interface (Fig. S11A), whereas in pair 9 they form a polar contact (E9:Lys97/Im9:Thr38) (Fig. S11A). Averaging out mutations in all other interface positions shows that this separation is driven by the low average fitness of variants with one of the intermediate mutational states, *i.e.*, E:Glu97/Im:Thr38, where E:Glu97 is not paired to a countercharge and is therefore strained (Fig. 3B & S11A). Such ordering of mutations increases the expected time it takes to evolve from one sequence to another, splitting the sequence space in the visualization.

Additionally, the high fitness sequences form an L-shaped pattern along the rim of each cluster in the visualization. The portion of the rim descending from E2/Im2 in the upper left corresponds to sequences that differ at the specificity-switching positions, E:98 and Im:33–34, and is mainly explained by incompatibilities between mutations at these positions that were already revealed by the experimental specificity map (Fig. 3A). In particular, Fig. 3C shows that several possible fit combinations against the background of the E2 identity E:Lys83 link the E2/Im2 sequence E2:Arg98/Im2:Asp33-Asn34 to the combination E:Met98/Im:Leu33-Val34 that exhibits E9/Im9-like specificity (Fig. 2D). Other combinations are predicted to have low average fitness, *e.g.* the combination E:Met98/Im:Asp33-Asn34 which was experimentally shown to be nonfunctional (Fig. 2D). Such low-fitness combinations restrict the order in which mutations can be accumulated, explaining why sequences that differ in these specificity-switching positions are separated in our visualizations. Notably, E:Met98/Im:Leu33-Val34 is predicted to be the fittest possible subsequence at these positions and context (Fig. 3C), explaining why the minimum-strain empirical path constructs this combination early and maintains it for most of its length.

The third set of interactions defines high-fitness sequences that ascend from the lower left to the upper right in the visualization (Fig. 3A). These are involved in the change in backbone conformation between E2/Im2 and E9/Im9 (Fig. 1C) that is associated with substantial changes in binding mechanism^34^ (Fig. S11 B&C). In the fitness landscape, these interface and backbone changes modify the sign of the effects of many mutations. For example, E:Lys72Asn and the adjacent E:Ala73Pro are both strongly deleterious at the start of this high-fitness region (Fig. 3D, left), but they later become only slightly deleterious individually and neutral in combination (Fig. 3D, center). Towards the end, with the addition of E:Thr77Ser (which relieves a steric clash with the Im9 helix 1 backbone, Fig. S11D), the double mutant becomes strongly beneficial (Fig. 3D, right). More generally, structure modeling suggests that the “L” shape of the high-fitness region arises because E:Lys83 is only compatible with a pair 2-like backbone (Fig. S11C) whereas E:Asn72 is only compatible with a pair 9-like backbone (Fig. S11B). As a result, the combination E:Asn72,Lys83 is incompatible with both structures leading to the large low-fitness regions in the top half of each of the three main clusters (Fig. S12).

We verified the robustness of our conclusions to modeling uncertainties. For example, while our analysis was performed assuming a quantitative fitness function (normalized MIC score), mutations that increase MIC score might not increase organismal fitness if fitness depends on survival/death in only a specific concentration of toxin. Nevertheless, we find that the structure of the fitness landscape and the main genetic interactions remain robust even when using a threshold-like fitness function along the whole range of reasonable cutoffs on the normalized MIC score (Fig. S13 and S14). Our analysis also remains robust to noise added to the Rosetta energies before the calibration procedure (Fig. S15). This robustness is likely due to the fact that the major aspects of the landscape structure depend on a small set of grossly incompatible combinations of mutations (such as E:Glu97/Im:Thr38). Correctly identifying these incompatibilities does not depend strongly on the details of the molecular force field or the precise choice of fitness function.

The global view of the predicted fitness landscape suggests several insights in addition to those we inferred from the empirical specificity map (Fig. 2D). First, we find that the empirically validated path resolves each epistatic set of interacting sites one at a time, likely minimizing strain by not superimposing incompatibilities (Fig. 3A, see also Fig. 2D). Second, by visualizing pairs that are orthogonal with respect to either E2/Im2 or E9/Im9, we find that each set is concentrated in a region of sequence space containing one of the extant protein pairs, (Fig. 3E,F) so that the evolution of functional orthogonality can be understood in terms of the population entering or exiting specific regions of sequence space. This analysis is robust to the thresholds used to define orthogonality (Fig. S16 & S17). Third, the global analysis suggests other mutations that may impact specificity, such as Im:Arg38Thr, that were not seen to impact specificity in the empirical path (Fig. S18 and S19).

### Adaptive mutations may drive functional divergence

The atomistic and graph-based calculations we employed are based solely on the physical constraints that a generic protein pair must meet to retain binding affinity. If these were the only determinants of fitness, we would expect natural populations to mutate neutrally among functional pairs. The fact that the pair forms a toxin-antitoxin system, however, can influence how populations evolve and functionally diverge. In this case, fitness depends not only on the ability of the immunity protein to neutralize its cognate endonuclease but also on whether the immunity protein can neutralize other endonucleases existing in the population and whether the cognate endonuclease can escape recognition by other immunity proteins, killing competing strains.

**Fig. 4.**
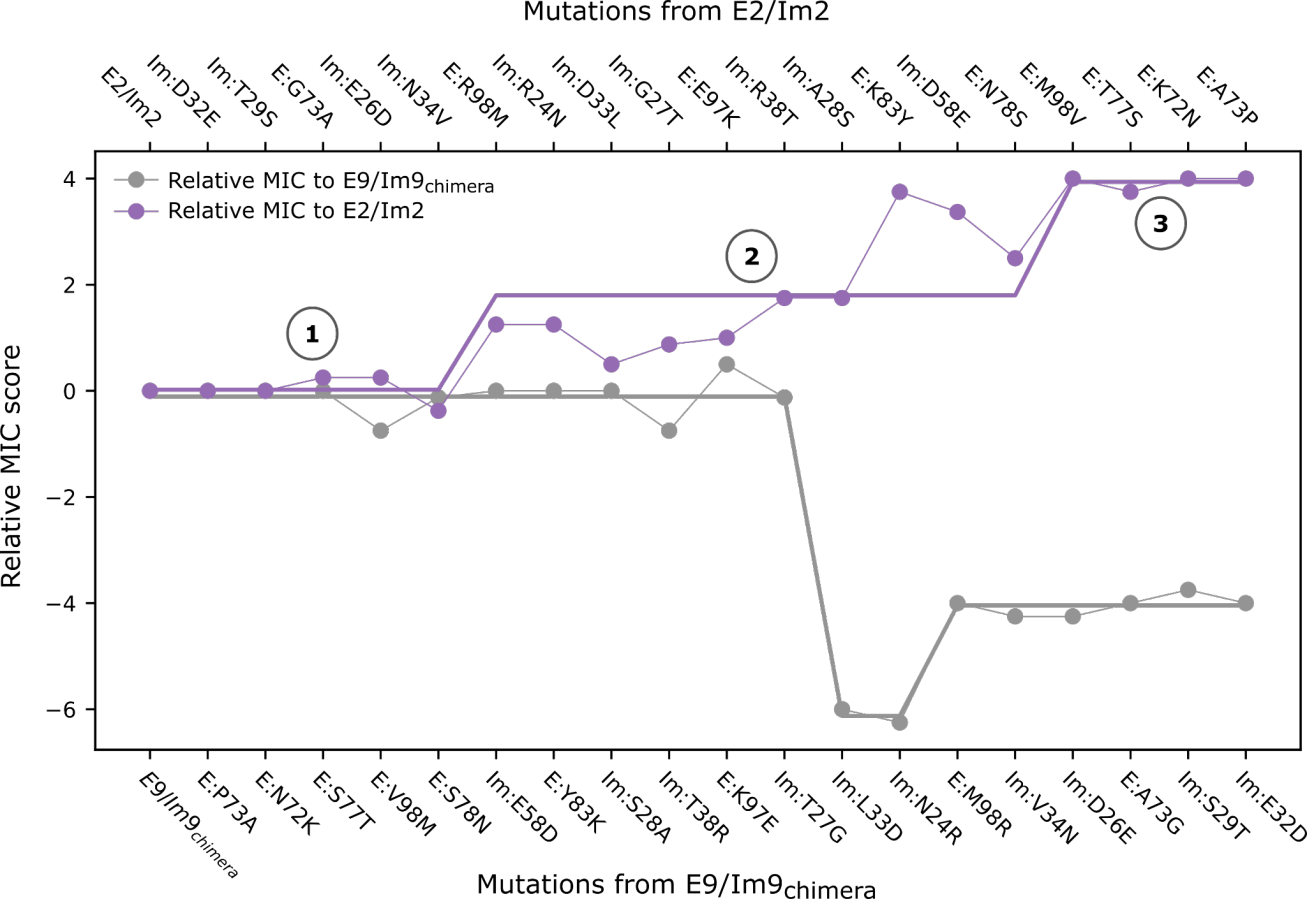
A “ratchet-like” model for the evolution of ultrahigh specificity. The experimentally determined viability of each cognate pair on the path relative (Fig. 2D) to the extant E2/Im2 (purple line, top *x*-axis) or E9/Im9 (gray, bottom *x*-axis). Viability increases in two steps in the direction of accumulating mutations from E2/Im2 and decreases in the reverse direction. Straight lines are guides to the eye. Higher viability corresponds to higher normalized MIC score. Relative MIC score (relative viability score) is the difference in MIC scores between a mutant and the parental wild type: ${RM}_{i}=\left(M_{E(i)/Im(i)}-M_{E(i)/Im(wt)}\right)-\left(M_{E(wt)/Im(wt)}-M_{E(wt)/Im(i)}\right)$, where RM is the relative MIC score; M is the MIC score (see Methods); *i* is an index to the mutant; wt is the parental protein, E2/Im2 or E9/Im9_chimera_.

Given these considerations, we re-examined the functional specificity map (Fig. 2D) to investigate the direction, if any, in which the experimental path is adaptive or maladaptive. We tracked how the relative MIC score (Methods) changes along the path with respect to each extant pair. In this simplified analysis, an intermediate exhibits high fitness relative to the parental population if its Im protein resists the parental endonuclease and its endonuclease escapes resistance by the parental Im protein (Equation in Figure 4 caption). While any sequence in the path could act as a hypothetical ancestor, we started by assuming that evolution begins at the E2/Im2 pair or mutants that are phenotypically similar, and that it proceeds in a population dominated by such pairs (Fig. 4, purple line, Step 1). After a series of neutral mutations that do not affect binding specificity, the enabling mutation Im:Asn34Val expands the repertoire of endonucleases that the Im can neutralize, including E:Met98. At the same time, Im:Asn34Val maintains binding affinity towards the ancestral E2. The following mutation, E:Arg98Met (Step 2), introduces an E variant that is, for the first time, cytotoxic towards the parental bacteria harboring an Im2-like protein while its cognate Im protein protects against E2-like endonucleases (Fig. 2D). Thus, the E:Arg98Met mutation is not only tolerated but may have an adaptive advantage that would allow it to penetrate, persist and spread within the parental population. The E:Arg98Met mutant, however, does not form an optimal interaction with the Im protein prior to incorporating the Im:Asp33Leu mutation, and the Met98-containing endonucleases are only partially cytotoxic towards Im2-containing hosts (Fig. 2D). Thus, Im:Asp33Leu improves the viability of this pair relative to the E:Arg98Met, and in Step 3, the codon-bridging mutation is resolved to the E9 identity through E:Met98Val. At this point, the endonuclease gains high cytotoxicity against the Im2-like harboring bacteria and completes the functional transition. In contrast, in a starting population dominated by an E9/Im9-like pair, the reverse trajectory passes through at least one maladaptive step (Fig. 4, gray line): Im:Leu33Asp reduces viability relative to the Im9-like proteins and is strongly susceptible to killing by parental E9-like endonucleases (Fig. 2D). In this simplified analysis, the adaptive mutations may provide a “ratchet”-like mechanism in which each adaptive step reduces the likelihood of the reverse mutation and increases the likelihood of accumulating further function-switching mutations.

There are limitations to our analyses, however. Although viability was measured *in vivo* and in the native host, experimental measures of functionality may not be completely faithful to viability in a wild population in nature. Despite these limitations, our results provide an example of how the specific nature of a protein-protein interaction, *i.e.*, a toxin-antitoxin system, may influence evolutionary paths, transforming what are *a priori* neutral mutations (with respect to binding) into adaptive or deleterious ones depending on the dominant genotypes in the population.

## Discussion

We showed how a charged protein-protein interface could transform, one-mutation-at-a-time, into a largely hydrophobic one without compromising stability, affinity and catalytic activity below their functional threshold in any intermediate step. To achieve this, we found that a codon-bridging mutation temporarily reduces the specificity barriers, permitting mutations that would otherwise reduce stability or binding affinity. Unlike previously reconstructed specificity switches where the intermediates were promiscuous “generalist” binders^2^, however, here the specificity switch occurs suddenly, with the key bridging mutation E:Arg98Met eliminating the interaction with Im2 and creating a new interaction with Im9 in a single step. Furthermore, while the structure of the genetic code is known to constrain the possible paths to evolve a new specificity^12^, our results provide an example in which the extra biochemical diversity provided by bridging mutations is likely essential to obtain a viable path between the orthogonal pairs.

A key question in the evolution of orthogonal binding pairs is how ultrahigh specificity evolves by a single-mutation trajectory without crossing a fitness valley. Our results provide a case-study in which each of the specificity-switching mutations are not only tolerated but may endow their host with a selective advantage relative to the parental population due to functional asymmetry in the interacting pair, as in a toxin-antitoxin system. This polarizes the function-altering evolutionary process, increasing the likelihood of selecting a long series of mutations, whereas the reverse mutations are counterselected. In other words, the functional asymmetry in the toxin-antitoxin system suggests preferred directions for the evolutionary process depending on specific environmental conditions.

This work introduces energy-based modeling as an approach to study both the overall shape and the fine details of a highly epistatic fitness landscape. The approach is general and can be implemented, in principle, to suggest evolutionarily plausible trajectories that link wild type proteins or inferred ancestral states, complementing and extending existing experimental and computational methods^2,3,12,15–19^. It allows full enumeration of elaborate molecular interaction networks and may provide detailed mechanistic insights on the evolution of microbial resistance mutations and the evolution of new activities in enzymes and binders informing how to design new protein activity.

## Methods

### Atomistic modeling

We enumerated all possible combinations of mutations and modeled them in Rosetta on the backbones of E2-Im2 and E9-Im9 (PDB codes: 3u43 and 1emv, respectively). Each mutant was modeled using all-atom Rosetta calculations, including combinatorial sidechain packing, and backbone and side chain minimization subject to harmonic restraints on the Cα coordinates^42^. Each mutant was modeled on the structures of both E2-Im2 and E9-Im9. All intermediates were ranked according to a composite score based on “fuzzy”-logic design (W)^32^ (Eq. 1) accounting for the difference of all-atom energy $\left(\Delta \Delta G_{s}\right)$ and difference of binding energy $\left(\Delta \Delta G_{b}\right)$ compared to the two wild type sequences.

(1) $$W=-ln\left(\frac{1}{1\;+\;e^{0.7\times \left(\Delta \Delta G_{S}-5\right)}}\right)-ln\left(\frac{1}{1+e^{0.7\times \left(\Delta \Delta G_{b}-5\right)}}\right)$$

For each sequence, the higher (more favorable) composite score was selected from the two calculations.

### Finding a least-frustration path using a graph-theoretical shortest-path algorithm

The sequence space comprised approximately 130,000 sequences in the initial four formulations (using a binary sequence space) and 2.4 × 10^6^ for the sequence space with codon-bridging mutations. We computed a truncated graph by taking the top 5% nodes with the highest composite scores. Nodes were connected by an edge if they differed by one amino acid substitution and contained all the previous mutations. The weight of each edge was assigned as *W* (Eq. 1) of the preceding node. Then, the Bellman-Ford shortest-path algorithm^39^ was applied to find the path with the lowest sum of scores (least strained). In the graph that contained amino acids that served as DNA bridging mutations, edges were added to connect mutants that could not be crossed with single-nucleotide mutations.

In the preliminary work that resulted in the four low-strain paths that were not viable (Fig. S2 and S3), the graphs were constructed using somewhat different ways but with the same scoring rules as described above. Briefly, in (1) the interface was divided into three spatially separated modules. Then the shortest path was found inside each module and between the modules. In (2) intermediates that exhibited more than 7 R.e.u. difference in system energy were eliminated. (3) intermediates with binding energy difference to wild type or binding strain difference to wild type above 2.5 and 1.5 R.e.u. respectively were eliminated and a graph was formed with the remaining nodes. In this trial, we applied another Rosetta filter, BindingStrain, which calculates the rotameric strain of monomers upon binding^43^. The intermediate scores in those three paths were calculated using Rosetta energy function, talaris14. And in (4) the graph was formed as the graph that contains the bridging mutations, but after eliminating the bridging nodes. In this fourth graph and the one with bridging mutations we used the new Rosetta energy function, ref15, to model and rank the intermediates. This energy function improves the treatment of electrostatics and solvation relative to the talaris14 energy function we previously used for the first three paths^38^.

### Number of viable paths in the experimental specificity map

We compute the number of viable directed, shortest-path (no reversions) evolutionary trajectories based on the data from the empirical specificity map (Fig. 2D). We note first that the space of viable intermediates in the specificity map is roughly separated by a bottleneck into two large grid-like blocks (2 × 6 and 8 × 5). The number of paths between two corners of an n by k grid is given by:

$$\frac{(n+k-2)!}{(n-1)!(k-1)!}$$

The total number of paths through both blocks is the product of the number of paths in each block. However, the calculation on the first block is slightly more complicated, because the path can exit via the mutation E:Arg98Met before or after Im:Arg24Asn. As a result, the total number of paths through the first block is equal to the sum of the number of paths between the corners of a 2×6 grid and the number of paths between the corners of a 2×5 grid. We thus obtain the following for the total number of unique paths:

$$\left(\frac{(6+2-2)!}{(6-1)!(2-1)!}+\frac{(5+2-2)!}{(5-1)!(2-1)!}\right)\times \frac{(5+8-2)!}{(5-1)!(8-1)!}=11\times 330=3,630.$$

### Cloning

pET21d plasmid harboring ColE2 wild type gene (full gene including R, T, and C domains) followed by Im2 wild type gene (separated by a 2-bp frameshift) with a C-terminal His_6_-tag^34^ was used as the basis for cloning. Synthetic genes for the two wild types and the intermediates of the computed least-strain path were codon-optimized for efficient *E. coli* expression and custom synthesized as linear fragments by TWIST Bioscience. The genes were swapped with the wild type gene by restriction-free (RF) cloning^44,45^. The plasmids were transformed into T7 Express $lysY/I^{q}$ *E.coli* cells (NEB), and DNA was extracted for Sanger sequencing to validate accuracy. Validated plasmids of the intermediates were also tested for viability by transformation to BL21 DE3 *E. coli* cells. An intermediate pair was considered viable if a lawn of bacteria was observed in the plate. Three colonies were picked for repeat sequencing to validate that no additional mutations were introduced. Sequence-validated colonies of BL21 *E. coli* were stored in glycerol stock at −80°C for expression and purification.

### Protein expression and purification

BL21 (DE3) cultures were grown in Luria Broth (LB) with 100 mM ampicillin medium at 37 °C to OD_600_=0.6–0.8 and induced with 1 mM IPTG at 16 °C for 20 hrs. Cells were harvested and stored at −20 °C. Pellet was resuspended in 10 ml lysis buffer in 200 ml culture containing 50 mM Tris (pH 8.6), 50 mM NaCl, 10 mM imidazole, 2 mM MgCl_2_, benzonase (1:500), and protease inhibitor (1:1000) sonicated in 3 cycles of 20 sec On and 40sec Off (1 min On-time) and centrifuged as previously described^34^. The supernatant was loaded onto a column packed with 1.2 ml Ni-NTA beads for 200ml culture, equilibrated with lysis buffer, and incubated at 4°C for 1hr for binding, then washed with lysis buffer containing 20 mM imidazole, and eluted with lysis buffer containing 500 mM imidazole. Protein complexes were dialyzed overnight in 50 mM Tris (ph 8.6), 200 mM NaCl, and 2 mM MgCl_2_. Protein complexes were stored at 4°C for a week and for longer times at −80°C.

Im proteins were purified using the same protocol as above with minor changes. The pH for the buffers was adjusted to 7.3, they were grown in 50 ml culture and all quantities were adjusted accordingly. Melting curves for the purified Im proteins were measured using Tyco NT.6 (Nanotemper Technologies Inc) at a rate of 30 °C/min.

All protein concentrations were measured using absorbance at 280nm. The concentrations for the ColE/Im complexes range between 2–3.5 mg/ml and Im protein concentrations range between 1.9–3 mg/ml.

### ColE/Im *in vivo* assay (spot-test)

The biological activities of ColE and Im proteins were determined using a modified form of the spot-test described in refs. ^46,47^. Briefly, ColE-sensitive *E. coli* JM83 (Addgene) cells were transformed with a control vector (empty pET21d) or one of the Im proteins and incubated in LB containing 100 mM Ampicillin at 37°C overnight. Then, 1 ml of culture was plated on agar plates to create a lawn of bacteria. Purified ColE/Im complexes (concentration between 2–4 mg/ml) were 6 times diluted in 10-fold serial dilutions and 8 μl aliquots were dropped on the plates containing bacteria. Plates were incubated overnight at 37°C. Clear zones represent cell death and the absence of clear zones indicates biological protection conferred by the Im. The minimal inhibitory concentration (MIC; the concentration dilution of ColE in which no clear zones were observed) was set as the last visually apparent drop (0, 1, 2, 3, 4, 5, or 6). The normalized MIC score is then computed as the difference between the maximal killing (the number of drops against an empty plasmid) and the MIC. All viability assays were conducted using two technical repeats and averages and standard deviations are shown in Fig. 2D and Table S5.

### Calibrating normalized MIC scores against Rosetta binding energies

In this section, we aimed to define a model that allows us to transform the computationally predicted binding energies for each E/Im pair sequence 𝑖𝑖 into the more biologically relevant scale of the normalized MIC score that takes into account the order of magnitude increase in the tolerated concentration of E toxin. The binding energy of each E/Im pair was calculated using both the E2/Im2 and E9/Im9 backbones. The minimum of the two is chosen for each pair, taking into account a free parameter γ corresponding to the difference in binding energy between the wildtype E9/Im9 and E2/Im2 structures:

$$\Delta \Delta G_{binding,i}=min\left(\Delta \Delta G_{E2/Im2,i},\Delta \Delta G_{E9/Im9,i}+\gamma \right)$$

Because the Im can only increase but not decrease the tolerance, the normalized MIC score cannot be lower than 0. To take this into account, we model the expected normalized MIC score $\left({\overset{‾}{y}}_{i}\right)$ as a function of the computationally predicted wild-type normalized binding energy $\Delta \Delta G_{binding,i}$:

$${\overset{‾}{y}}_{i}=log\left(1+e^{\;\alpha +\;\beta \Delta \Delta G_{binding,i}}\right)$$

However, we cannot measure directly ${\overset{‾}{y}}_{i}$ but only obtain data with experimental errors. Moreover, as we have data only for six concentrations, our observed normalized MIC score will not be higher than the number of tested concentrations, but the real underlying score could indeed be larger. Thus, we define a piecewise likelihood function for dependence on the observed $y_{i}$, together with the standard deviation σ for unexplained variation:

$$p\left(y_{i}|{\overset{‾}{y}}_{ı},\sigma \right)=p\left(y_{i}\geq 6|\overset{‾}{y},\sigma \right)\;\text{if}\;y_{i}=6\;p\left(y_{i}|{\overset{‾}{y}}_{ı},\sigma \right)=p\left(y_{i}\leq 0|\overset{‾}{y},\sigma \right)\;\text{if}\;y_{i}<0\;p\left(y_{i}|{\overset{‾}{y}}_{ı},\sigma \right)=p\left(y_{i}|\overset{‾}{y},\sigma \right)\;\text{if}\;0<y_{i}<6$$

The data from the specificity map computed in Fig. 2D show consistent normalized MIC scores across the two biological replicates (Pearson r=0.99, Fig. S10A). We fit the model by maximizing the likelihood using the Nelder-Mead algorithm; that is, we find the combination of values for α,β,γ,σ that maximizes the likelihood, *i.e.*, the probability of observing the data under our calibration model. Using the maximum likelihood estimate ($\overset{ˆ}{\alpha }=2.31,\;\overset{ˆ}{\beta }=-0.91,\;\overset{ˆ}{\gamma }=-9.46,\;\overset{ˆ}{\sigma }=1.24$), we can make predictions given the computed binding energies for any E/Im pair. While our predictions can yield values beyond 6, in order to have predicted normalized MIC scores in the same range as the empirical path, scores were truncated to 6. Performance in both training and leave-one-out held out data is excellent (cross-validated Pearson r=0.92, Fig. S10B,C). Moreover, the vast majority of the data lie within the range of binding energies spanned by the experimentally validated data, suggesting that we can effectively interpolate the normalized MIC scores for nearly the full Im/E sequence space (Fig. S10D).

### Fitness landscape visualization

Visualization method as previously described^41^. Briefly, we construct a model of molecular evolution where a population evolves via single amino acid substitutions and the rate at which each possible substitution becomes fixed in the population reflects its selective advantage or disadvantage relative to the currently fixed sequence. More specifically, in our model the rate of evolution from sequence i to any mutationally adjacent sequence j is given by

$$Q_{ij}=\frac{S_{ij}}{1\;-\;e^{S_{ij}}}$$

where $S_{ij}$ is the scaled selection coefficient (population size times the selection coefficient of j relative to i), time is measured relative to the amino acid mutation rate (each possible amino acid mutation occurs at rate 1), and the total leaving rate from each sequence i is given by

$$Q_{ii}=-\;\sum_{j\neq i}Q_{ij}$$

In the current context, $S_{ij}=c\left(f_{j}-f_{i}\right)$, where $f_{i}$ is the predicted normalized MIC score at sequence i. For this analysis we choose c so that the equilibrium expected normalized MIC score is 2, corresponding to a roughly threefold increase relative to the expected normalized MIC score of 0.76 under neutrality, and close to the predicted normalized MIC score of the wild-type E2/Im2 of 2.40.

Given the rate matrix Q for our evolutionary model, we then construct the visualization by using the subdominant right eigenvectors associated with the smallest magnitude non-zero eigenvalues of this rate matrix as coordinates for the low dimensional representation of the landscape, where each such coordinate defines one of the “diffusion axes” used in the visualization. This produces a visualization that reflects the long-term barriers to diffusion in sequence space, and, in particular, clusters of sequences in the visualization correspond to sets of initial states from which the evolutionary model approaches its stationary distribution in the same manner, and multi-peaked fitness landscapes appear as broadly separated clusters with one peak in each cluster. Moreover, by scaling the axes appropriately, as is done here, these axes can be given units of $\sqrt{time}$ and it can be shown that the resulting distances reflect evolutionary times under this model. In particular, using these coordinates, the squared Euclidean distance between arbitrary sequences i and j optimally approximates (in a specific sense) the sum of the expected time to evolve from i to j and the expected time to evolve from j to i. See ref. ^41^ for details.

## Supplementary Material

Supplement 1

## References

[1] Poelwijk F. J., Kiviet D. J., Weinreich D. M. & Tans S. J. Empirical fitness landscapes reveal accessible evolutionary paths. Nature 445, 383–386 (2007). 17251971 10.1038/nature05451

[2] Aakre C. D. Evolving new protein-protein interaction specificity through promiscuous intermediates. Cell 163, 594–606 (2015). 26478181 10.1016/j.cell.2015.09.055 PMC4623991

[3] Levin K. B. Following evolutionary paths to protein-protein interactions with high affinity and selectivity. Nat. Struct. Mol. Biol. 16, 1049–1055 (2009). 19749752 10.1038/nsmb.1670

[4] Siddiq M. A., Hochberg G. K. & Thornton J. W. Evolution of protein specificity: insights from ancestral protein reconstruction. Curr. Opin. Struct. Biol. 47, 113–122 (2017). 28841430 10.1016/j.sbi.2017.07.003 PMC6141201

[5] McClune C. J. & Laub M. T. Constraints on the expansion of paralogous protein families. Curr. Biol. 30, R460–R464 (2020). 32428482 10.1016/j.cub.2020.02.075

[6] Meenan N. A. G. The structural and energetic basis for high selectivity in a high-affinity protein-protein interaction. Proc. Natl. Acad. Sci. U. S. A. 107, 10080–10085 (2010). 20479265 10.1073/pnas.0910756107 PMC2890441

[7] Papadakos G., Wojdyla J. A. & Kleanthous C. Nuclease colicins and their immunity proteins. Q. Rev. Biophys. 45, 57–103 (2012). 22085441 10.1017/S0033583511000114

[8] Kuriyan J., Konforti B. & Wemmer D. The molecules of life: Physical and chemical principles. (Garland Science, 2012).

[9] Mohammadi M., Olsen S. K. & Ibrahimi O. A. Structural basis for fibroblast growth factor receptor activation. Cytokine Growth Factor Rev. 16, 107–137 (2005). 15863029 10.1016/j.cytogfr.2005.01.008

[10] Itoh N. & Ornitz D. M. Evolution of the Fgf and Fgfr gene families. Trends Genet. 20, 563–569 (2004). 15475116 10.1016/j.tig.2004.08.007

[11] Starr T. N. & Thornton J. W. Epistasis in protein evolution. Protein Sci. 25, 1204–1218 (2016). 26833806 10.1002/pro.2897 PMC4918427

[12] Podgornaia A. I. & Laub M. T. Protein evolution. Pervasive degeneracy and epistasis in a protein-protein interface. Science 347, 673–677 (2015). 25657251 10.1126/science.1257360

[13] Bridgham J. T., Carroll S. M. & Thornton J. W. Evolution of hormone-receptor complexity by molecular exploitation. Science 312, 97–101 (2006). 37000978 10.1681/01.asn.0000926836.46869.e5

[14] Welin M. & Nordlund P. Understanding specificity in metabolic pathways—Structural biology of human nucleotide metabolism. Biochem. Biophys. Res. Commun. 396, 157–163 (2010). 20494131 10.1016/j.bbrc.2010.04.054

[15] Starr T. N., Picton L. K. & Thornton J. W. Alternative evolutionary histories in the sequence space of an ancient protein. Nature 549, 409–413 (2017). 28902834 10.1038/nature23902 PMC6214350

[16] McClune C. J., Alvarez-Buylla A., Voigt C. A. & Laub M. T. Engineering orthogonal signalling pathways reveals the sparse occupancy of sequence space. Nature 574, 702–706 (2019). 31645757 10.1038/s41586-019-1639-8 PMC6858568

[17] Poelwijk F. J., Socolich M. & Ranganathan R. Learning the pattern of epistasis linking genotype and phenotype in a protein. Nature Communications vol. 10 Preprint at 10.1038/s41467-019-12130-8 (2019). PMC6746860 31527666

[18] Sarkisyan K. S. Local fitness landscape of the green fluorescent protein. Nature 533, 397–401 (2016). 27193686 10.1038/nature17995 PMC4968632

[19] Weinreich D. M., Delaney N. F., DePristo M. A. & Hartl D. L. Darwinian evolution can follow only very few mutational paths to fitter proteins. Science 312, 111–114 (2006). 16601193 10.1126/science.1123539

[20] Ding D. Co-evolution of interacting proteins through non-contacting and non-specific mutations. Nat Ecol Evol 6, 590–603 (2022). 35361892 10.1038/s41559-022-01688-0 PMC9090974

[21] Nocedal I. & Laub M. T. Ancestral reconstruction of duplicated signaling proteins reveals the evolution of signaling specificity. Elife 11, (2022). 10.7554/eLife.77346 PMC9208753 35686729

[22] Hochberg G. K. A. & Thornton J. W. Reconstructing Ancient Proteins to Understand the Causes of Structure and Function. Annu. Rev. Biophys. 46, 247–269 (2017). 28301769 10.1146/annurev-biophys-070816-033631 PMC6141191

[23] Gong L. I., Suchard M. A. & Bloom J. D. Stability-mediated epistasis constrains the evolution of an influenza protein. Elife 2, e00631 (2013). 23682315 10.7554/eLife.00631 PMC3654441

[24] Harms M. J. & Thornton J. W. Evolutionary biochemistry: revealing the historical and physical causes of protein properties. Nat. Rev. Genet. 14, 559–571 (2013). 23864121 10.1038/nrg3540 PMC4418793

[25] Dean A. M. & Thornton J. W. Mechanistic approaches to the study of evolution: the functional synthesis. Nat. Rev. Genet. 8, 675–688 (2007). 17703238 10.1038/nrg2160 PMC2488205

[26] Cramer W. A., Sharma O. & Zakharov S. D. On mechanisms of colicin import: the outer membrane quandary. Biochem. J 475, 3903–3915 (2018). 30541793 10.1042/BCJ20180477

[27] Li W. Highly Discriminating Protein–Protein Interaction Specificities in the Context of a Conserved Binding Energy Hotspot. J. Mol. Biol. 337, 743–759 (2004). 15019791 10.1016/j.jmb.2004.02.005

[28] Keeble A. H., Kirkpatrick N., Shimizu S. & Kleanthous C. Calorimetric dissection of colicin DNase--immunity protein complex specificity. Biochemistry 45, 3243–3254 (2006). 16519519 10.1021/bi052373o

[29] Kirkup B. C. & Riley M. A. Antibiotic-mediated antagonism leads to a bacterial game of rock-paper-scissors in vivo. Nature 428, 412–414 (2004). 15042087 10.1038/nature02429

[30] Kortemme T. Computational redesign of protein-protein interaction specificity. Nat. Struct. Mol. Biol. 11, 371–379 (2004). 15034550 10.1038/nsmb749

[31] Netzer R. Ultrahigh specificity in a network of computationally designed protein-interaction pairs. Nat. Commun. 9, 5286 (2018). 30538236 10.1038/s41467-018-07722-9 PMC6290019

[32] Warszawski S., Netzer R., Tawfik D. S. & Fleishman S. J. A ‘fuzzy’-logic language for encoding multiple physical traits in biomolecules. J. Mol. Biol. 426, 4125–4138 (2014). 25311857 10.1016/j.jmb.2014.10.002 PMC4270444

[33] Keeble A. H. Experimental and computational analyses of the energetic basis for dual recognition of immunity proteins by colicin endonucleases. J. Mol. Biol. 379, 745–759 (2008). 18471830 10.1016/j.jmb.2008.03.055

[34] Wojdyla J. A., Fleishman S. J., Baker D. & Kleanthous C. Structure of the ultra-high-affinity colicin E2 DNase-Im2 complex. J. Mol. Biol. 417, 79–94 (2012). 22306467 10.1016/j.jmb.2012.01.019

[35] Kosloff M., Travis A. M., Bosch D. E., Siderovski D. P. & Arshavsky V. Y. Integrating energy calculations with functional assays to decipher the specificity of G protein-RGS protein interactions. Nat. Struct. Mol. Biol. 18, 846–853 (2011). 21685921 10.1038/nsmb.2068 PMC3130846

[36] Sharp C., Bray J., Housden N. G., Maiden M. C. J. & Kleanthous C. Diversity and distribution of nuclease bacteriocins in bacterial genomes revealed using Hidden Markov Models. PLoS Comput. Biol. 13, e1005652 (2017). 28715501 10.1371/journal.pcbi.1005652 PMC5536347

[37] Kondrashov D. A. & Kondrashov F. A. Topological features of rugged fitness landscapes in sequence space. Trends Genet. 31, 24–33 (2015). 25438718 10.1016/j.tig.2014.09.009

[38] Alford R. F. The Rosetta All-Atom Energy Function for Macromolecular Modeling and Design. J. Chem. Theory Comput. 13, 3031–3048 (2017). 28430426 10.1021/acs.jctc.7b00125 PMC5717763

[39] Bang-Jensen J. & Gutin G. Z. Digraphs: Theory, Algorithms and Applications. (Springer, 2008).

[40] Ortlund E. A., Bridgham J. T., Redinbo M. R. & Thornton J. W. Crystal structure of an ancient protein: evolution by conformational epistasis. Science 317, 1544–1548 (2007). 17702911 10.1126/science.1142819 PMC2519897

[41] McCandlish D. M. Visualizing fitness landscapes. Evolution 65, 1544–1558 (2011). 21644947 10.1111/j.1558-5646.2011.01236.x PMC3668694

[42] Khersonsky O. Automated Design of Efficient and Functionally Diverse Enzyme Repertoires. Mol. Cell 72, 178–186.e5 (2018). 30270109 10.1016/j.molcel.2018.08.033 PMC6193528

[43] Fleishman S. J., Khare S. D., Koga N. & Baker D. Restricted sidechain plasticity in the structures of native proteins and complexes. Protein Sci. 20, 753–757 (2011). 21432939 10.1002/pro.604 PMC3081553

[44] Unger T., Jacobovitch Y., Dantes A., Bernheim R. & Peleg Y. Applications of the Restriction Free (RF) cloning procedure for molecular manipulations and protein expression. J. Struct. Biol. 172, 34–44 (2010). 20600952 10.1016/j.jsb.2010.06.016

[45] Erijman A., Dantes A., Bernheim R., Shifman J. M. & Peleg Y. Transfer-PCR (TPCR): a highway for DNA cloning and protein engineering. J. Struct. Biol. 175, 171–177 (2011). 21515384 10.1016/j.jsb.2011.04.005

[46] Wallis R. In vivo and in vitro characterization of overproduced colicin E9 immunity protein. Eur. J. Biochem. 207, 687–695 (1992). 1633820 10.1111/j.1432-1033.1992.tb17096.x

[47] Wallis R. Protein-Protein Interactions in Colicin E9 DNase-Immunity Protein Complexes. 2. Cognate and Noncognate Interactions That Span the Millilmolar to Femptomolar Affinity Range. Biochemistry vol. 34 13751–13759 Preprint at 10.1021/bi00042a005 (1995). 7577967

[48] Li W. & Godzik A. Cd-hit: A fast program for clustering and comparing large sets of protein or nucleotide sequences. Bioinformatics 22, 1658–1659 (2006). 16731699 10.1093/bioinformatics/btl158

[49] Edgar R. C. MUSCLE: a multiple sequence alignment method with reduced time and space complexity. BMC Bioinformatics 5, 113 (2004). 15318951 10.1186/1471-2105-5-113 PMC517706

[50] O’Meara M. J. A Combined Covalent-Electrostatic Model of Hydrogen Bonding Improves Structure Prediction with Rosetta. J. Chem. Theory Comput. 11, 609–622 (2015). 25866491 10.1021/ct500864r PMC4390092

[51] Lite T.-L. V. Uncovering the basis of protein-protein interaction specificity with a combinatorially complete library. Elife 9, (2020). 10.7554/eLife.60924 PMC7669267 33107822

[52] Crona K. Rank orders and signed interactions in evolutionary biology. Elife 9, (2020). 10.7554/eLife.51004 PMC7000213 31934856

[53] Conrad M. The geometry of evolution. Biosystems. 24, 61–81 (1990). 2224072 10.1016/0303-2647(90)90030-5

