# Supplementary material for: Evolutionary paths that link orthogonal pairs of binding proteins
Source: Res Sq. 2023 Dec 13:rs.3.rs-2836905. Originally published 2023 Apr 20. Preprint. [Version 2] doi: 10.21203/rs.3.rs-2836905/v2 (PMC10153392; doi:10.21203/rs.3.rs-2836905/v2)
Supplement: Supplement 1 [file NIHPPRS2836905V2-supplement-1.pdf]

Supplementary information

Supplementary tables

Position	Chain	E2/Im2	E9-Im9	Bridging mutation
**24**	**A**	R	N	K
**26**	**A**	E	D	
**27**	**A**	G	T	S
**28**	**A**	A	S	
**29**	**A**	T	S	
**32**	**A**	D	E	
**33**	**A**	D	L	V
**34**	**A**	N	V	D
**38**	**A**	R	T	
**58**	**A**	D	E	
**72**	**B**	K	N	
**73**	**B**	G	P	A
**77**	**B**	T	S	
**78**	**B**	N	S	
**83**	**B**	K	Y	N
**97**	**B**	E	K	
**98**	**B**	R	V	M

Supplementary Table 1. The 17 interfacial positions that differ between E2/Im2 and E9/Im9 and the resulting expanded sequence space after adding single codon-bridging amino acid to the two parental identities. Position numbering and chains according to E2/Im2 PDB entry: 3u43.

E2/Im2 wt	MESKRNKPGKATGKGKPVGDKWLDDAGKDSGAPIPDRIADKLRDKEFKNFDDFRKKFWEEV<br>SKDPDLSKQF**KG**SNK**TN**IQKG**K**APFARKKDQVGGR**ER**FELHHDKPISQDGGVYDMNNIRVTT<br>PKRHIDIHRGK/MELKHSISDYTEAEFLEFVKKIC**R**A**EGAT**EE**DDN**KLV**R**EFERLTEHPDGSDLI<br>YYPR**D**DREDSPEGIVKEIKEWRAANGKSGFKQGLE
E9/Im9 wt	MESKRNKPGKATGKGKPVGDKWLDDAGKDSGAPIPDRIADKLRDKEFKSFDDFRKAVWEEV<br>SKDPELSKNL**NP**SNK**SS**VSKG**Y**SPFTPKNQQVGGR**KV**YELHHDKPISQGGEVYDMDNIRVTTP<br>KRHIDIHRGK/MELKHSISDYTEAEFLQLVTTIC**N**A**DTSS**EE**ELV**KLV**T**HFEEMTEHPSGSDLIY<br>YPK**E**GDDDSPSGIVNTVKQWRAANGKSGFKQGLE
E9/Im9 chimera	MESKRNKPGKATGKGKPVGDKWLDDAGKDSGAPIPDRIADKLRDKEFKNFDDFRKKFWEEV<br>SKDPDLSKQF**NP**SNK**SS**IQKG**YA**PFARKKDQVGGR**KV**YELHHDKPISQDGGVYDMNNIRVTT<br>PKRHIDIHRGK/MELKHSISDYTEAEFLEFVKKIC**N**A**DTSS**EE**ELV**KLV**TE**FERLTEHPDGSDLI<br>YYPR**E**DREDSPEGIVKEIKEWRAANGKSGFKQGLE

Supplementary Table 2. The sequences of the wild type colicins E2/Im2 and E9/Im9 and the sequence of E9/Im9$_{chimera}$. The 17 interfacial positions are marked in bold and orange font. The 33 changes outside of the interface are marked on the sequence of E9/Im9$_{chimera}$ in red font.

Accession #	72	73	77	78	83	97	98
E2 wt	K	G	T	N	K	E	R
E9 wt	N	P	S	S	Y	K	V
EHW1683184	S	*	A	G	*	*	*
EAO6252864	S	*	A	G	*	*	*
EBP0523054	S	*	*	G	*	*	*
WP_216691228	*	*	*	*	*	*	*
WP_042969090	S	*	*	G	*	*	*
EET0447945	S	*	*	G	*	*	*
WP_149013229	S	*	*	G	*	*	*
WP_001684089	S	*	*	G	*	*	*
WP_139571364	S	*	*	G	*	*	*
HAY0237856	*	D	*	*	*	*	*
MCD3753904	*	*	*	*	*	*	*
CAA25609	*	*	*	*	*	*	*
EGO0637768	*	*	*	*	*	*	*
WP_078194592	*	*	*	*	*	*	*
EJN0406533	*	*	*	*	*	*	*
WP_136767443	*	*	*	*	*	*	*
PDU80939	*	*	*	*	*	*	*
EFU5689591	*	*	*	*	*	*	*
WP_096845574	*	*	*	*	*	*	*
EFM7227381	*	*	*	*	*	*	*
HBA5420398	*	*	*	*	*	*	*
EFJ1015634	*	*	*	*	*	*	*
MBZ9087339	*	*	*	*	*	*	*
MBW0659285	*	*	*	*	*	*	*
HAW1183305	*	D	*	*	*	*	*
EDD1161141	*	D	*	*	*	*	*
WP_141070407	*	D	*	*	*	*	*

Supplementary Table 3. Natural diversity in ColE interfacial positions. E9 homologs were collected from the nr database using protein BLAST with an 80% sequence identity cutoff. The sequences were clustered using CD-HIT[48] with 99% cutoff. The resulting 28 unique sequences were aligned with MUSCLE[49]. E2-like identities are shown in green and E9-like identities in blue.

Accession #	24	26	27	28	29	32	33	34	38	58
Im2 wt	**R**	**E**	**G**	**A**	**T**	**D**	**D**	**N**	**R**	**D**
Im9 wt	**N**	**D**	**T**	**S**	**S**	**E**	**L**	**V**	**T**	**E**
WP_171149985	E	*	*	E	*	*	H	*	A	*
WP_045973231	D	*	*	E	*	*	H	*	D	*
WP_086956852	D	*	*	E	*	*	H	H	*	*
WP_047963342	D	*	*	E	*	*	H	*	*	A
WP_166540812	*	T	*	*	N	*	H	K	*	*
EAR6896980	*	*	*	*	*	*	H	K	*	*
ECN5337130	*	*	*	*	*	*	H	K	*	*
HAS8350052	*	*	*	*	*	*	H	K	*	*
WP_193830442	*	*	*	*	*	*	H	K	*	*
WP_143345855	L	*	*	*	*	*	*	*	D	*
WP_139766971	*	*	*	*	*	*	*	*	*	-
WP_025672426	*	*	*	*	*	*	*	*	*	*
EYX73168	*	*	*	*	*	*	*	*	*	*
BAI39401	*	*	*	*	*	*	*	*	*	*
WP_000861935	*	*	*	*	*	*	*	*	*	*
EHV4529714	*	*	*	*	*	*	*	*	*	*
HAW1068311	*	*	*	*	*	*	*	*	*	*
HAG5762455	*	*	*	*	*	*	*	*	*	*
WP_032326735	*	*	*	*	*	*	*	*	*	*
EGD6078999	*	*	*	*	*	*	*	*	*	*
HAU9627203	*	*	*	*	*	*	*	*	*	*
EGT2301001	*	*	*	*	*	*	*	*	*	*
EGE7374601	*	*	*	*	*	*	*	*	*	*
EFI9877364	*	*	*	*	*	*	*	*	*	*
WP_096167538	*	*	*	*	*	*	*	T	*	*
EET6468166	*	*	*	*	*	*	*	*	*	*
EFL5137489	*	*	*	*	*	*	*	*	*	*

EFJ0587121	*	*	*	*	*	*	*	*	*	*
HAO1928242	*	*	*	*	*	*	*	*	*	*
WP_000861936	*	*	*	*	*	*	*	*	*	*
WP_106642707	*	*	*	*	*	*	*	*	*	*
WP_109043749	*	*	*	*	*	*	E	*	*	*
2WPT_A	*	*	*	*	*	*	*	*	*	*
EDP6088977	*	*	*	*	*	*	*	*	*	*
WP_136770323	*	*	*	*	*	*	*	*	*	*
WP_077108249	*	*	*	*	*	*	*	*	*	*
WP_000420693	*	*	*	*	*	*	*	*	*	*
WP_176563135	*	*	*	*	*	*	*	*	*	*
WP_153443728	*	*	*	*	*	*	*	*	*	*
WP_256488494	*	*	*	*	*	*	*	*	*	*
WP_097455943	*	*	*	*	*	*	*	*	*	*
WP_228106873	*	*	*	*	*	*	*	*	*	*
WP_112861050	*	*	*	*	*	*	*	T	*	*
HAZ3685192	*	*	*	*	*	*	*	*	*	*
WP_000420692	*	*	*	*	*	*	*	*	*	*
WP_035600465	S	*	*	*	*	*	*	D	*	*
ECJ2224819	S	*	*	I	*	*	*	T	*	*
MBA2139178	S	*	*	*	*	*	*	*	*	*
EAW1321402	S	*	*	*	*	*	*	*	*	*
WP_262955291	S	*	*	*	*	*	*	*	*	*
WP_015097263	D	S	A	R	*	*	*	*	L	*
WP_001684088	*	*	*	*	*	*	Q	*	S	*
HCB8098444	*	*	*	*	*	*	Q	*	S	*
WP_042969089	*	*	*	*	*	*	Q	*	S	*
WP_077168007	*	*	*	*	*	*	Q	*	N	*
WP_261232817	*	*	*	*	*	*	*	D	D	*
HBH7052729	*	*	*	*	*	*	*	I	*	K

EFB6209868	*	*	*	*	*	*	*	*	*	*
1BXI_A	*	*	*	*	*	*	*	*	*	*
2GZI_A	*	*	*	*	*	*	*	A	*	*
MBZ9013147	*	*	*	*	*	*	*	*	A	*
AAA23076	*	N	*	*	*	*	*	*	*	*
2GZF_A	*	*	*	*	*	*	*	*	*	*
EET1502589	*	*	*	*	*	*	*	*	*	*
1FR2_A	*	*	*	*	*	*	*	*	*	*
2GYK_A	*	*	*	*	*	*	*	*	*	*
2GZE_A	*	*	*	*	*	*	*	*	*	*
WP_012644887	*	*	*	*	*	*	*	*	*	*
2GZG_A	*	*	*	*	*	*	*	*	*	*
3GKL_C	D	*	A	T	*	*	*	D	*	*
3GJN_A	D	*	A	T	*	*	*	D	*	*
EAA8037545	*	*	*	*	*	*	Q	*	*	*
EDS5484017	*	*	*	*	*	*	Q	I	*	*
EDW8121861	*	*	*	*	*	*	Q	I	*	*
EAB7210983	*	*	*	*	*	*	Q	I	*	*
EAO6252865	*	*	*	*	*	*	Q	I	*	*
ECG8633828	*	*	*	*	*	*	Q	I	*	*
WP_008912668	*	*	*	E	N	*	Q	I	S	*
WP_036952821	*	*	*	*	N	*	Q	I	*	*
WP_004914275	*	*	*	*	N	*	Q	I	*	*
EJD6670373	*	*	*	*	N	*	Q	I	*	*
WP_210838631	*	*	*	*	N	*	Q	I	*	*
WP_123382475	*	*	*	*	*	*	Q	I	*	*
MBO2879508	*	*	*	*	*	*	Q	I	*	*
HCE8607293	*	*	*	*	*	*	Q	I	*	*
WP_198633470	*	*	*	*	*	*	Q	I	*	*

WP_094960452	*	*	*	*	*	*	Q	I	*	*

Supplementary Table 4. Natural diversity in Im interfacial positions. Sequence selection and analysis as in Supplemental Table 3, except that here, we used a sequence identity cutoff of 65% to collect Im9 homologs.

Im/E	**E2**	**G73A**	**R98M**	**E97K**	**K83Y**	**N78S**	**M98V**	**T77S**	**K72N**	**A73P (E9$_{chimera}$)**	**E9**
**Im2**	4±0	4.5±0	2.87±0.18	3.75±0.35	5.75±0.35	4.5±0.71	6±0	5.75±0.35	6±0	6±0	6±0
**D32E**	4±0	4.75±0.35	3±0	3.75±0.35	5.37±0.18	4.25±0.35	6±0	5.75±0.35	6±0	5.75±0.35	5.5±0
**T29S**	4±0	4.25±0.35	3±0	4.37±0.18	5±0	4.25±0.35	5.75±0.35	5.75±0.35	5.87±0.18	6±0	5.37±0.18
**E26D**	4±0	4.25±0.35	4±0.35	5±0	5.75±0.35	4.75±0.35	5.87±0.18	5.75±0.35	6±0	6±0	5.37±0.53
**N34V**	4.4±0.18	4.5±0	1.25±1.06	3±0	4.25±0.35	3.25±0.35	5.25±0.35	5.25±0.35	5.5±0.35	5.5±0	5.5±0.35
**R24N**	4.4±0.18	4.25±0.35	1.25±0.35	2.5±0	4±0	2.75±0.35	5.375±0.53	5.25±0.35	5.5±0.71	5.25±0.35	5.25±0.35
**D33L**	6±0	6±0	0.37±0.53	1±1.41	0±0	0±0	0.25±0.35	0.25±0.35	0±0	0.25±0.35	3±0
**G27T**	6±0	6±0	0±0	0.75±0.35	0±0	0±0	0±0	0±0	0±0	0±0	1.75±0.35
**R38T**	6±0	6±0	0.75±0.35	0±0	0±0	0±0	0±0	0±0	0±0	0±0	1.5±0
**A28S**	6±0	6±0	0.75±0.35	0±0	0±0	0±0	0±0	0±0	0±0	0±0	1.5±0
**D58E (Im9$_{chimera}$)**	6±0	6±0	0.5±0	0±0	0±0	0±0	0±0	0±0	0±0	0±0	0.75±0.35
**Im9**	6±0	6±0	4±0	3±0	0±0	0±0	2.25±0.35	2±1.41	1.75±1.78	2±1.41	3±0
**Naive cells**	6±0	6±0	5.25±0.35	6±0	5.875±0.18	5.25±0.35	6±0	6±0	6±0	6±0	6±0

Supplementary Table 5. Average MIC scores and standard deviations of two technical repeats of all ColE mutants on the evolutionary trajectory, applied to all Im proteins (vertical axis). The MIC scores are the number of killing zones (clear zones of cell death) that were observed for each pair (higher numbers translate to lower viability). Each drop corresponds to tenfold dilution of the purified ColE/Im pair. For example, four drops means that the last clear zone was observed at 10^{-3} dilution of the ColE/Im pair. Normalized MIC scores were calculated by subtracting the maximal killing (MIC on naive cells). These averages were used for the calculations of the specificity map in **Fig. 2D**. The mutations from E2/Im2 identity to E9/Im9$_{chimera}$ (E:A73P/Im:D58E) accumulate from the top-left corner to the bottom-right corner as in Figure 2D. A row and a column of wild type Im9 and E9 were added.

Supplementary figures

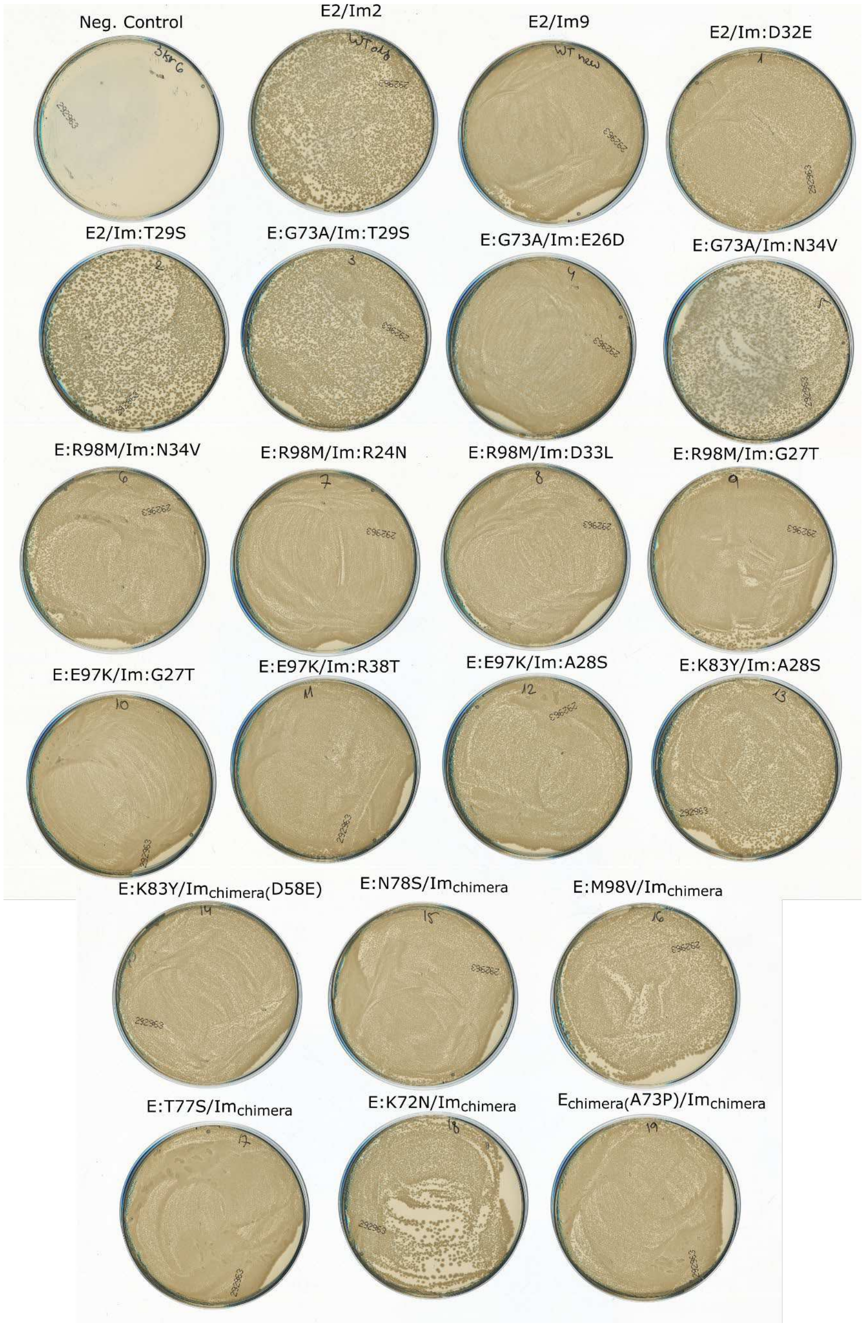

Supplementary Figure 1. Growth experiment for the intermediate of the minimum-strain path. E2/Im2, E9/Im9, negative control (E/Im design that does not grow in BL21 cells), the 18 intermediates and E9/$Im9_{chimera}$ (as marked above each plate) were cloned into BL21 *E.coli* cells and plated on selective agar plates (**Fig. 1E** step III). All clones exhibited similar growth except of the negative control that no growth was inspected.

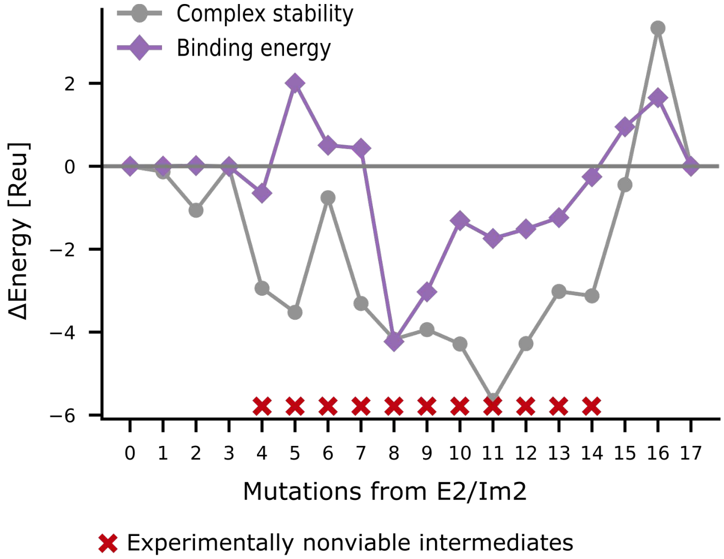

Supplementary Figure 2. The least-strain path was obtained from a graph computed with Rosetta energy function 15 (ref15) [38] and all bridging mutations were eliminated. The difference in complex stability and binding energy to the wild types is shown (*y*-axis) for each intermediate from the least-strain path. On the *x*-axis are marked the experimental results for growth in BL21, in which the (x) sign is for intermediates that did not show any growth.

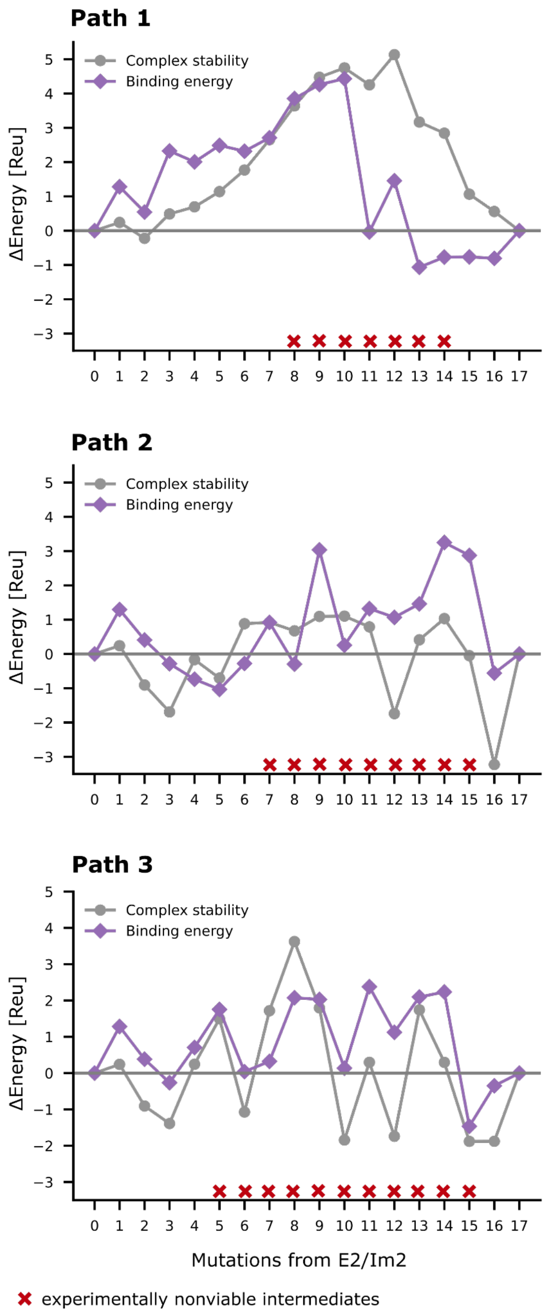

Supplementary Figure 3. Energy difference to wild types (E2/Im2 and E9/Im9) of complex stability and binding energy (*y*-axis) is shown for three low-strain paths from the first three trials, that were obtained from graphs computed with an older version of the Rosetta energy function, talaris14[50]. The experimental results for growth on selective agar plates after transformation into BL21 are shown by the red x sign for intermediates that did not show any growth and are hence considered to be nonviable. See Methods for a description of the differences between the three attempts.

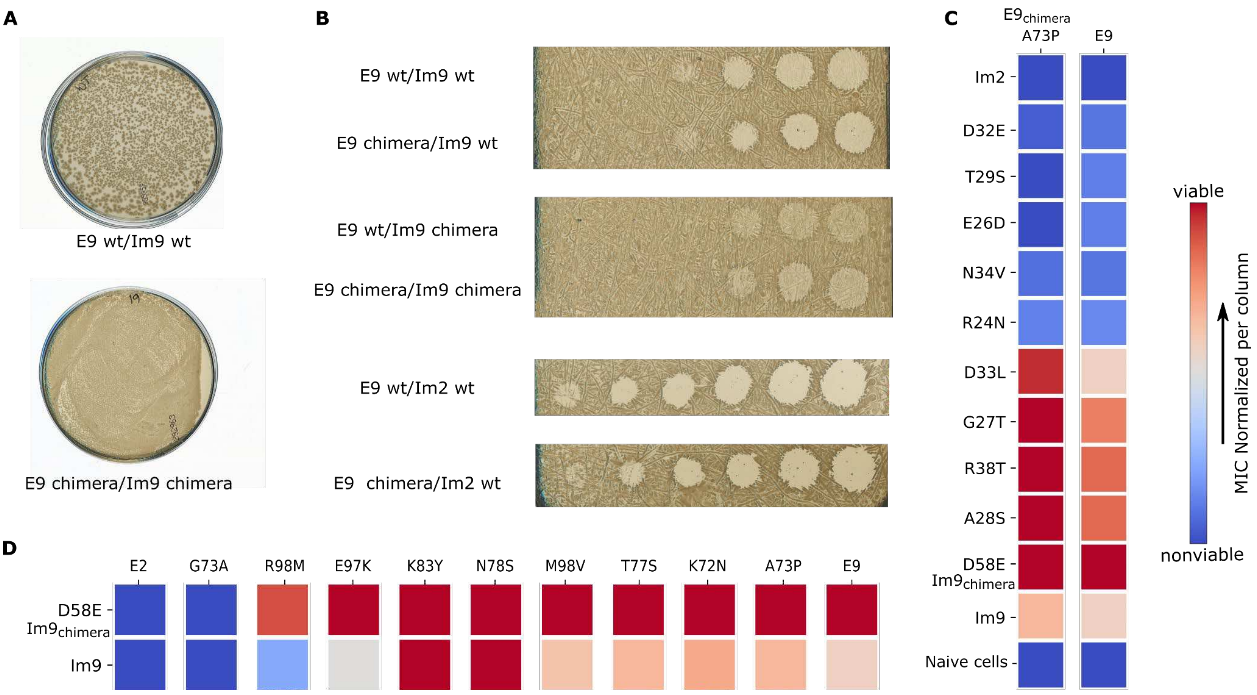

Supplementary Figure 4. Comparison between E9/Im9$_{chimera}$ and the wild-type E9/Im9 pair (A) The two pairs grow in BL21 cells as bacterial lawns. (B) *In vivo* activity testing shows that the two E9 variants induce similar killing levels and three Im variants (Im2, Im9 and Im9$_{chimera}$) provide similar levels of protection. Note that Im9$_{chimera}$ (with interface residues as in Im9 and the rest as in Im2) provides better protection against both E proteins than Im9. (C) E9 and E9$_{chimera}$ induce similar levels of killing towards all Im proteins. (D) Im9 and Im9$_{chimera}$ provide a similar level of protection. Note that Im9$_{chimera}$ provides a higher level of protection than Im9 (similar to the observation in panel B). We also note that Im9$_{chimera}$ exhibits higher stability than Im9 and Im2 as shown in Fig. S7.

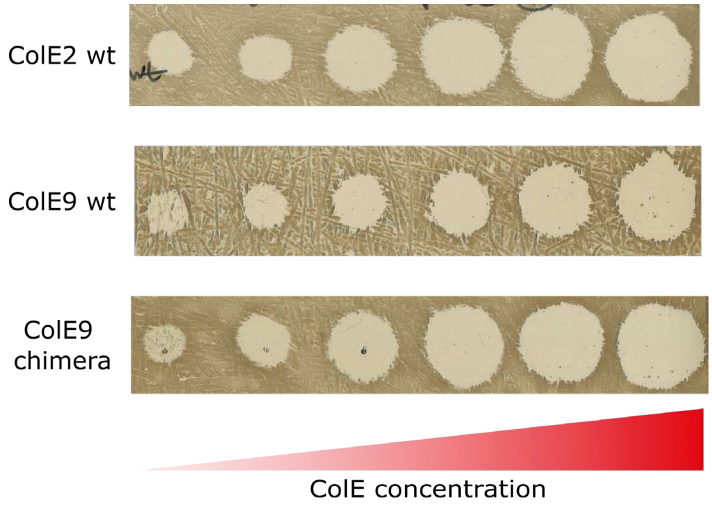

Supplementary Figure 5. In vivo activity of ColE9/Im9$_{chimera}$ compared to the two wild type complexes ColE2/Im2 and ColE9/Im9. The ColE/Im complexes were tested in an in vivo activity assay as explained in the methods. Here, they were applied to naive JM83 cells that do not harbor any Im protein. The ColE/Im complexes were diluted in six ten-fold serial dilutions. Meaning the concentration of the ColE decreases as marked by the gradient at the bottom. The ColEs exert killing in all concentrations that were tested.

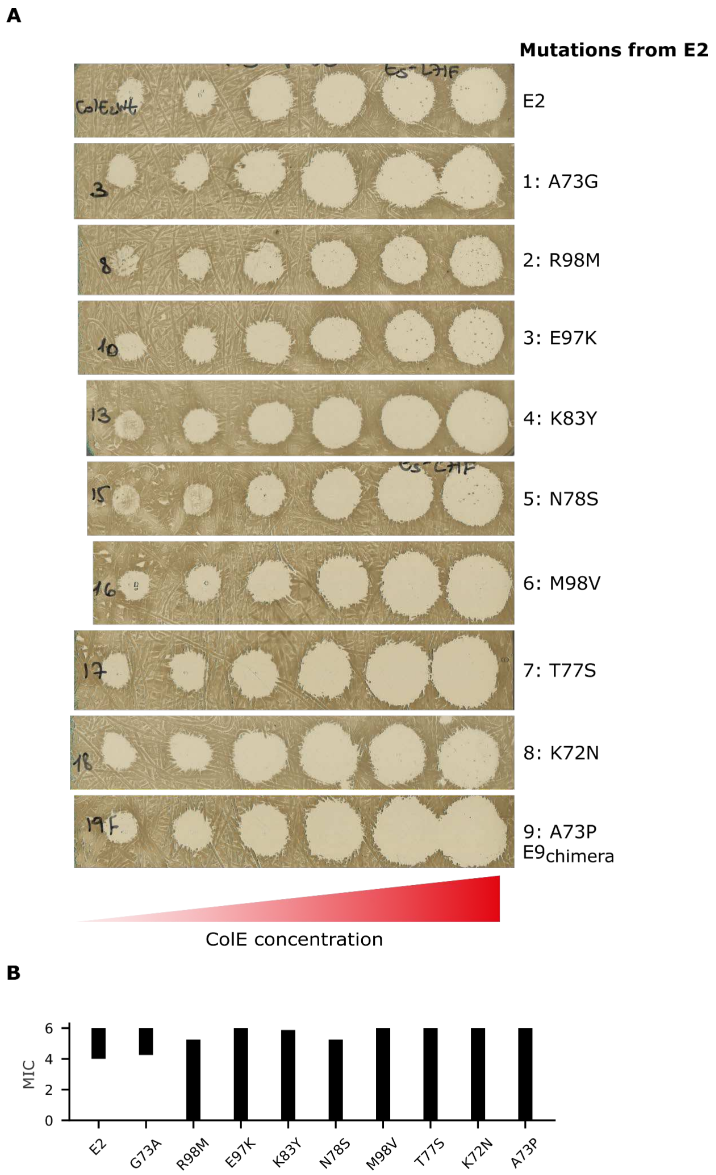

Supplementary Figure 6. (A) The activity of viable path ColEs. The 10 ColEs of the viable path were purified together with their cognate Im. The purified complexes were dropped in six tenfold serial dilutions against JM83 naive cells that do not harbor any Im protein (empty plasmid). All ColEs intermediates retained their toxicity. (B) The killing range of each of the 10 E proteins on the viable path. Maximal killing is the MIC when the E protein is applied to naive cells and the minimal killing is the lowest MIC which was observed when applying the E protein on the Im proteins from the viable path. Wild type E2 and the Gly73Ala mutant exhibit extreme killing efficiencies even against cognate Im2.

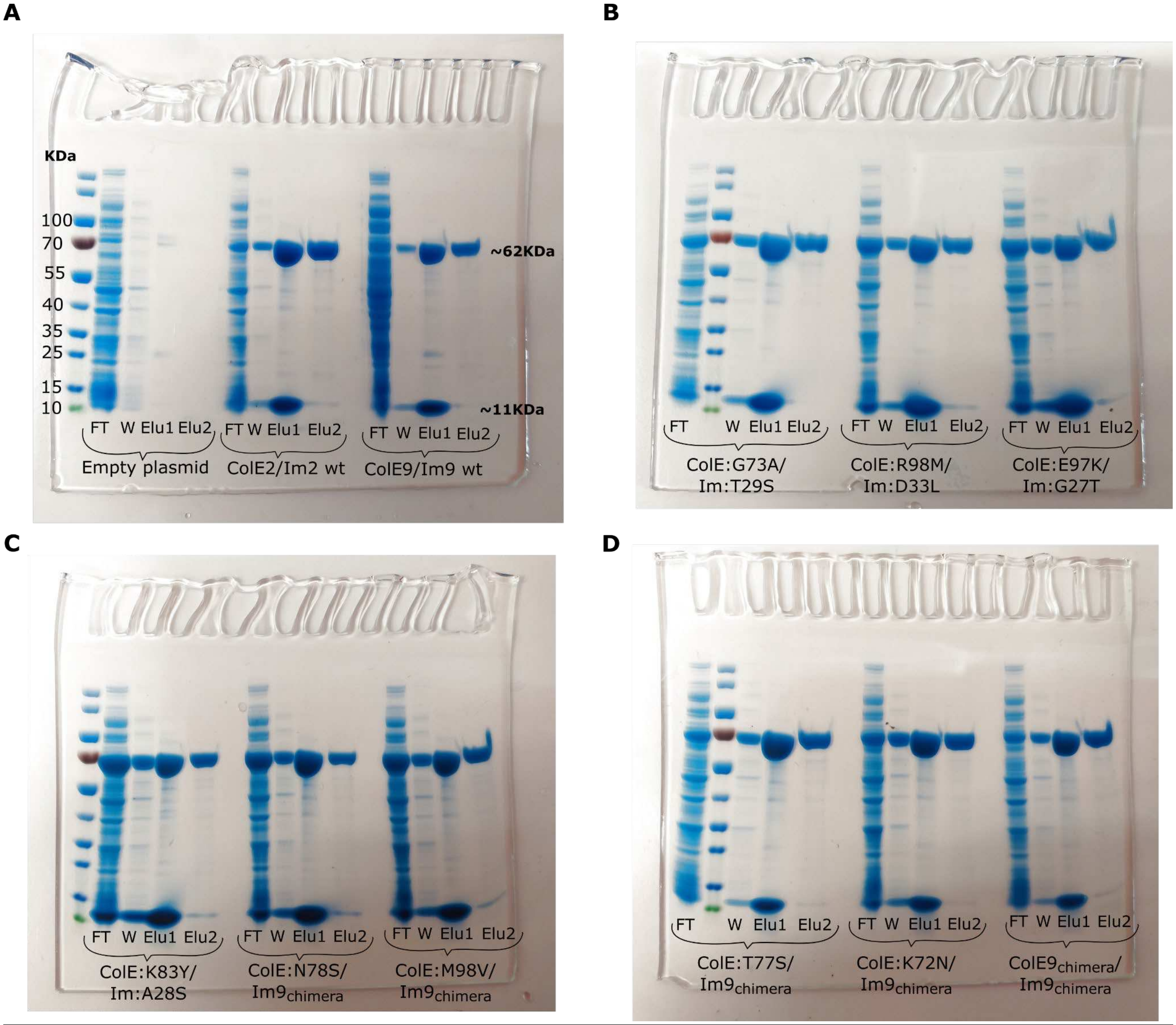

Supplementary Figure 7. Quantifying ColE/Im complex expression levels. In each gel there are three samples. FT - flow through from the Ni-NTA column; W - wash step (see Methods); Elu1 - eluted complex of ColE/Im; Elu2 - elution of the ColE alone. An unfolding step was done to separate the ColE from the Im and the flow through was collected. For the *in vivo* assay only the complexes were used to check the full biological activity. (A) Empty plasmid as a control, wild type ColE2/Im2 and wild type E9/Im9. (B-D) intermediate ColE/Im complexes. The expected sizes of the ColE and Im proteins are marked in (A) on the right hand side and the ladder sizes on the left.

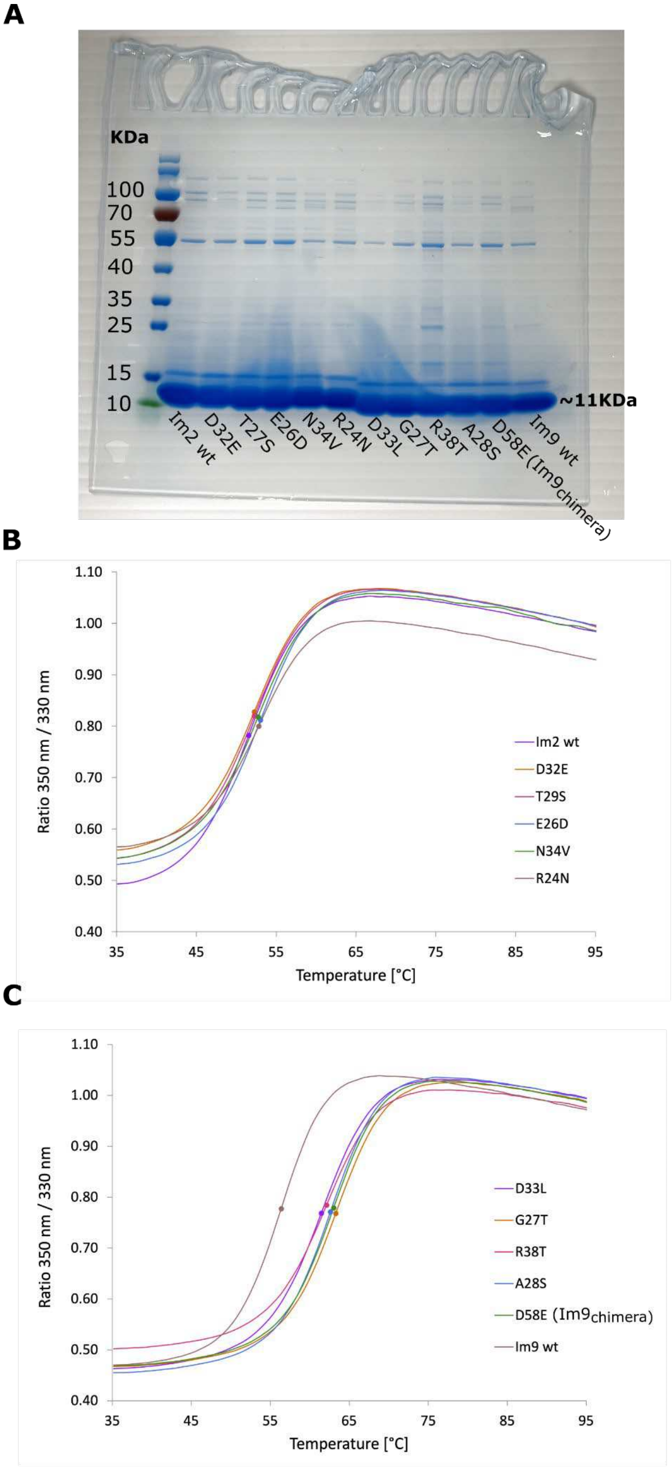

Supplementary Figure 8. Expression levels of Im proteins. (A) Wild type Im2 and Im9, intermediate immunity proteins, and $Im9_{chimera}$ expressed in BL21 cells and purified. All Im proteins exhibit similar expression levels. (B-C) Thermal melting for all Im proteins. All Im proteins exhibit an inflection above 50 °C and an apparent two-state behavior. The sizes of the Im protein is marked in (A) on the right hand side and the ladder sizes on the left.

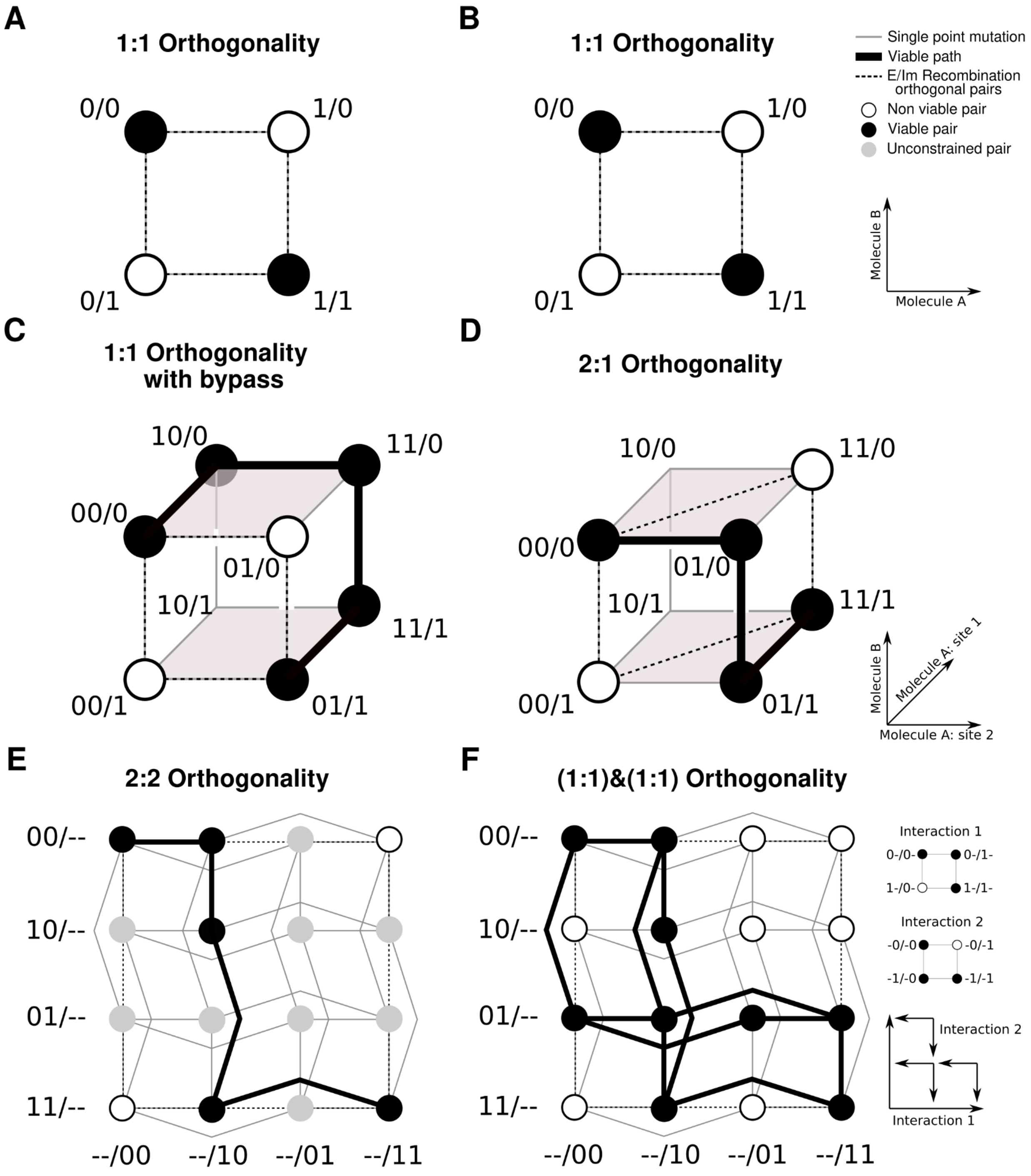

Supplementary Figure 9. Models of orthogonality in fitness landscapes for several distinct *a:b* types, where *a* and *b* are the number of mutations differentiating each of the orthogonal sequences in each molecule. Dotted lines join the four pairs that are involved in the orthogonality, directly reachable through recombination, and characterizing the required “rectangular perturbation” (see

Supplementary Information). Thick black lines represent possible viable paths between the orthogonal pairs in each model. (A) General condition for orthogonality and the genotypes involved for any fitness landscape. (B) Model for 1:1 orthogonality, in which the fitness landscape is fully determined by the orthogonality. (C) 1:1 orthogonality model with a viable path provided by an extra site in molecule 1 that serves as an extradimensional bypass. (D) 2:1 orthogonality model with one of the possible viable paths between the orthogonal pairs. (E) 2:2 orthogonality model showing one possible path between the orthogonal pairs. (F) 2:2 orthogonality model resulting from the combination of two simple 1:1 non orthogonal models with a single incompatibility and the resulting patterns of connectivity of viable pairs. The inset shows how interaction 1 involves the first position from each molecule while interaction 2 involves the second position from each molecule, and illustrates how these two underlying interactions combine to form the set of viable pairs shown in the larger figure

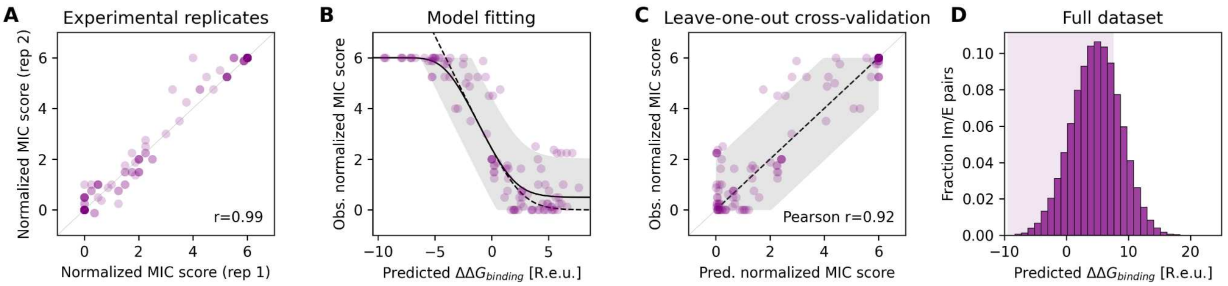

Supplementary Figure 10. Calibration of computational predictions using the specificity map measurements (Fig. 2D). (A) The correspondence between two biological replicates across all Im/E pairs along the experimental path. (B) Experimentally measured normalized MIC scores versus computationally predicted binding energies relative to E2/Im2. The dashed line represents the predictions for the theoretical normalized MIC scores in absence of experimental error. The solid line represents the expected measurement for the normalized MIC score taking into account our noise model and the saturation at a normalized MIC score of 6, corresponding to survival at the greatest tested E concentration. The shaded area represents the interval in which 95% of the measurements are expected to lie under the maximum likelihood estimates (91% of the data lies within those intervals). (C) Scatter plot of the observed normalized MIC scores against the calibrated computational predictions in leave-one-out settings. The shaded area represents the interval in which 95% of the measurements are expected to lie under the maximum likelihood estimates (91% of the data lies within those intervals). (D) Histogram showing the distribution of the adjusted computational predictions of the binding energy relative to E2/Im2 in the full dataset containing all E/Im pairs in the sequence space. The majority of the data lie within the range of energies used for the calibration procedure as shown by the shaded area.

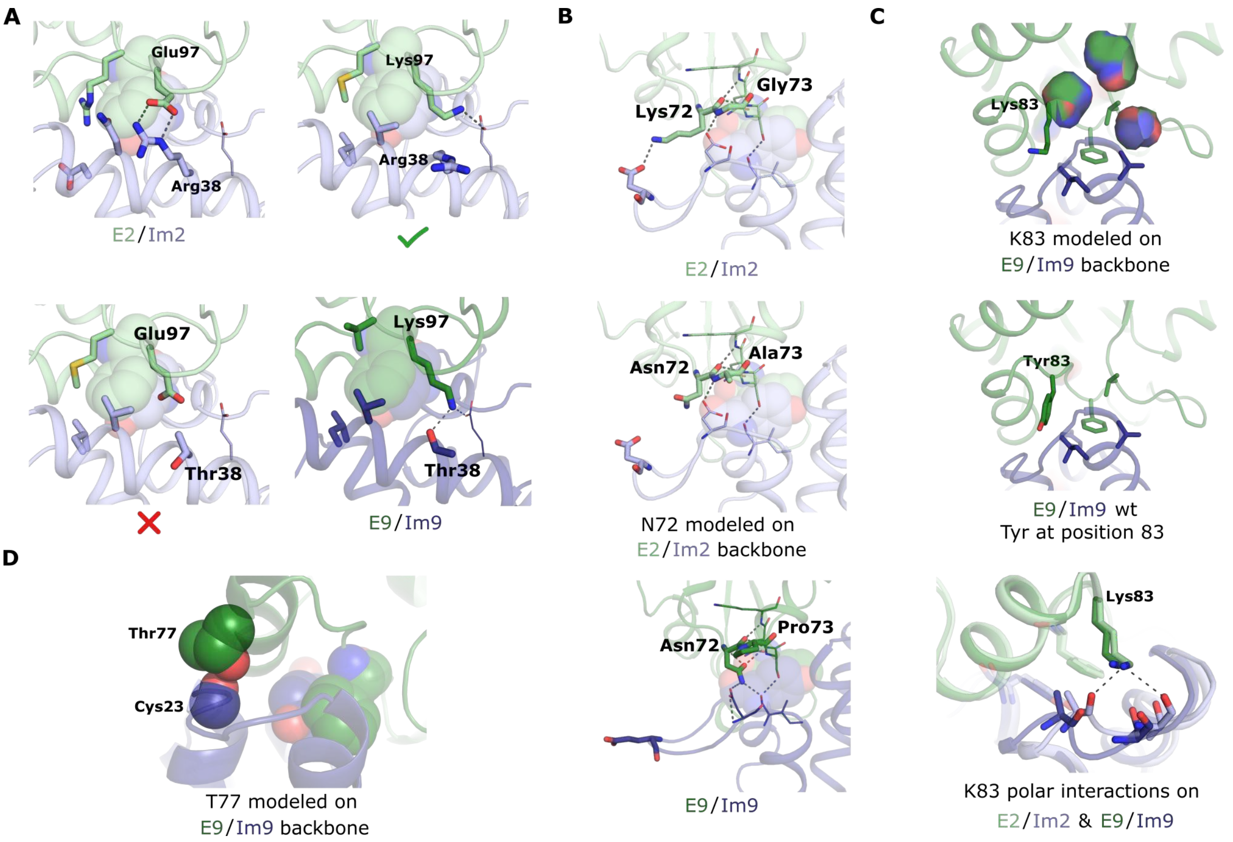

Supplementary Figure 11. Incompatibilities in volume, electrostatics, and backbone conformation separate mutants in the visualization of the fitness landscape (**Fig. 3A).** (A) The interaction between positions E:97 and Im:38. E2/Im2 harbors a salt bridge (top-left); the viable intermediate (marked with a green checkmark) forms an apolar interaction between hydrophobic groups on the side chains of E:Lys97 and Im:Arg38 with both positive charges facing away (top-right); the nonviable intermediate (marked with a red x) exhibits an unsatisfied and therefore strained E:Glu97; finally, in the wild type interface of E9/Im9, E:Lys97 forms stabilizing polar interactions with surrounding amino acids (bottom-right). (B) E2:Lys72 forms a salt bridge across the interface in E2/Im2. While E9:Asn72 modeled on the backbone of E2/Im2 abrogates a hydrogen-bond network that is observed in the E9/Im9 interface. (C) E2:Lys83 is incompatible with the E9/Im9 backbone, exhibiting interface cavities (shown as surfaces). The E9:Tyr83 fills these cavities. By contrast, E:Lys83 is compatible with the E2/Im2 backbone forming interfacial hydrogen bonds with backbone carbonyls. These bonds cannot form in the E9/Im9 backbone due to the increased distance between the two partners in this state. (D) The E2:Thr77 sidechain modeled in spheres on the E9/Im9 backbone. The rigid and bulky side chain overlaps with the immunity backbone. All models were generated and relaxed using the same atomistic Rosetta protocol used to generate the empirical path.

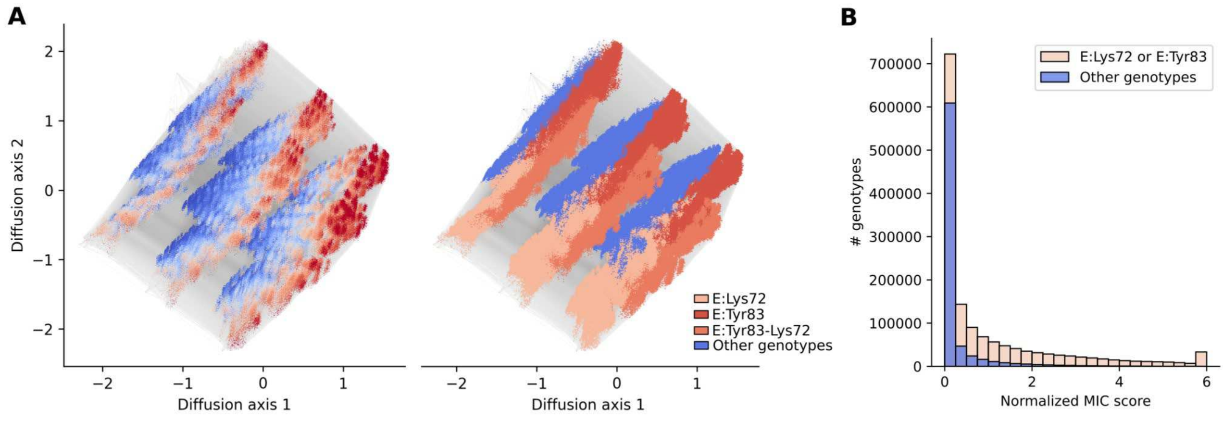

Supplementary Figure 12. Positions E:72 and E:83 define the regions of sequence space containing high fitness sequences. (A) Landscape colored by predicted normalized MIC score (left) and colored by the presence of E:Lys72 and/or E:Tyr83. (B) Histogram of predicted normalized MIC scores for sequences containing E:Lys72 and/or E:Tyr83 shown with histogram for all other sequences. The vast majority of predicted high fitness sequences contain at least one of E:Lys72 or E:Tyr83. Histograms for the two sets of genotypes are overlaid rather than stacked.

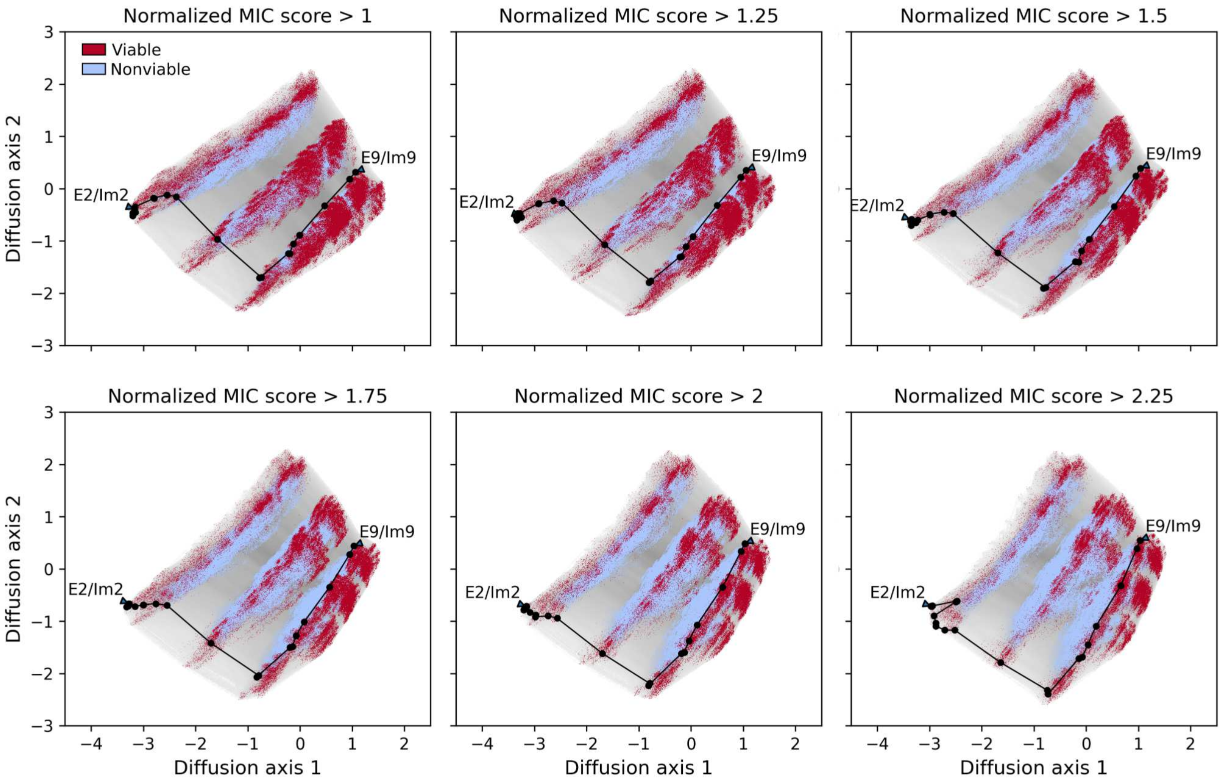

Supplementary Figure 13. Landscape structure is robust to a threshold-based binary fitness function over a wide range of possible cutoffs. Each shows the visualization of a threshold-based binary fitness landscape that arises by considering cell survival in an E toxin concentration given by a range of thresholds (i.e. all cutoffs less than the normalized MIC of the wild-type pairs E2/Im2 and E9/Im9 which have a minimum normalized MIC of approximately 2.5 and greater than the normalized MIC of the non-cognate pairs E9/Im2 and E2/Im9 that have a maximum normalized MIC of approximately 0.9). The experimentally validated path from Fig. 2 is highlighted in black and E2/Im2 and E9/Im9 wild-type positions are indicated with arrows. Parameter c for the visualizations was chosen to have an odds ratio of 9 when comparing the fraction of time that the population spends at viable pairs under purifying selection with that under neutral evolution.

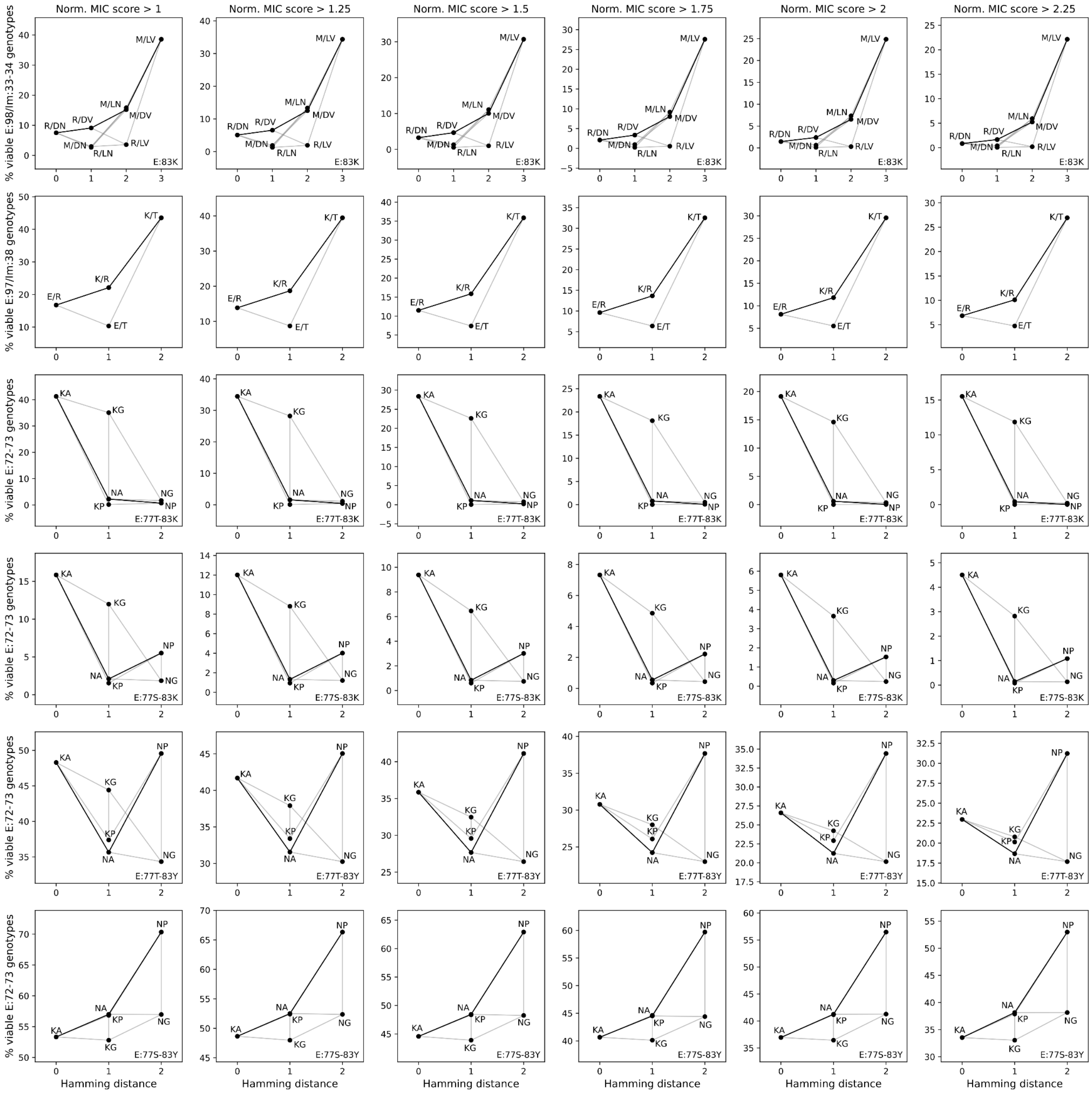

Supplementary Figure 14. Genetic interactions are robust to a threshold-based binary fitness function over a wide range of possible cutoffs. Each panel represents a sub-landscape obtained by averaging out positions not involved in the specific genetic interaction (shown along rows) and in the specified sequence context (shown on the bottom right corner of each panel, if any) as in Figure 3. The columns correspond to different tested thresholds along the range of reasonable viability cutoffs (*i.e.,* all cutoffs less than the normalized MIC of the wild-type pairs E2/Im2 and E9/Im9 which have a minimum normalized MIC of approximately 2.5 and greater than the normalized MIC of the non-cognate pairs E9/Im2 and E2/Im9 that have a maximum normalized MIC of approximately 0.9). Thick black lines represent the mutations that were included in the experimentally validated path.

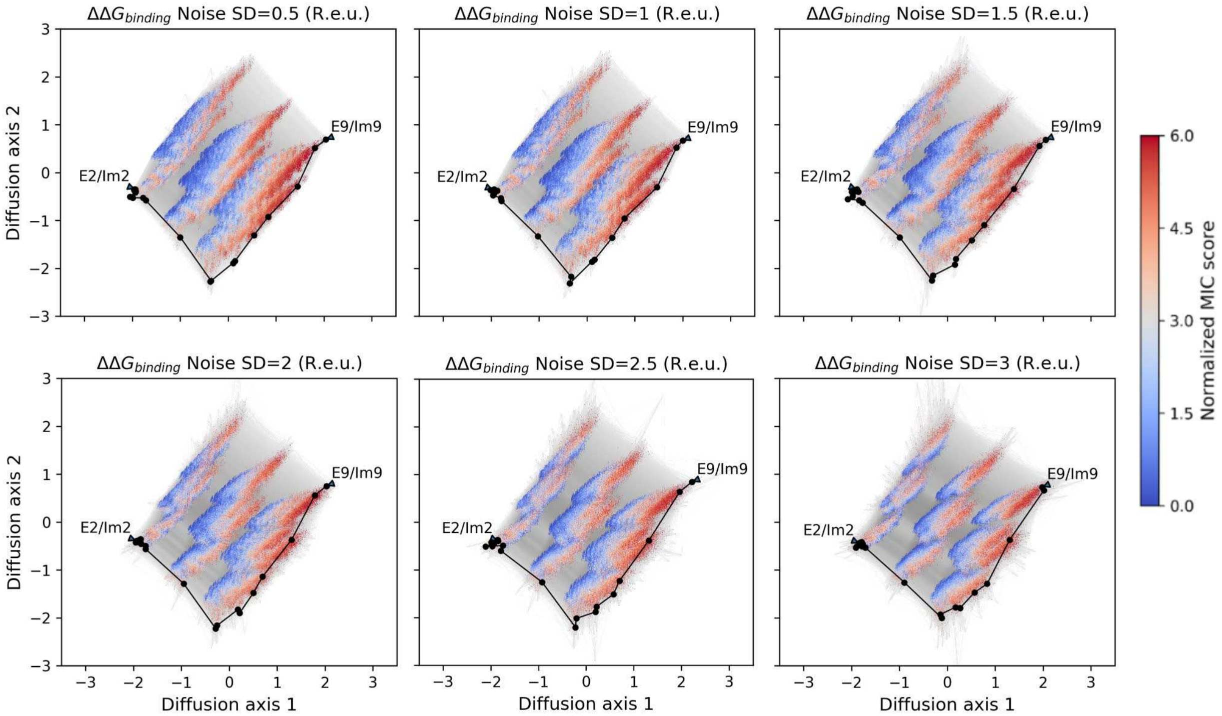

Supplementary Figure 15. Landscape structure is robust to noise in the Rosetta binding energies. Each shows the visualization of a landscape generated by adding a normally distributed error with standard deviations ranging from 0.5 to 3 R.e.u. to the predicted binding energies. The experimentally validated path from Fig. 2 is highlighted in black and E2/Im2 and E9/Im9 wild-type positions are indicated with arrows. To account for changes in the distribution of Normalized MIC scores after introducing noise in the binding energies when computing the coordinates of the visualization, parameter *c* was chosen to have an increase of 3.5 fold when comparing the ratio of mean(Normalized MIC score) / (6 - mean(Normalized MIC score)) in the stationary phase for a population evolving under purifying selection with that under neutral evolution.

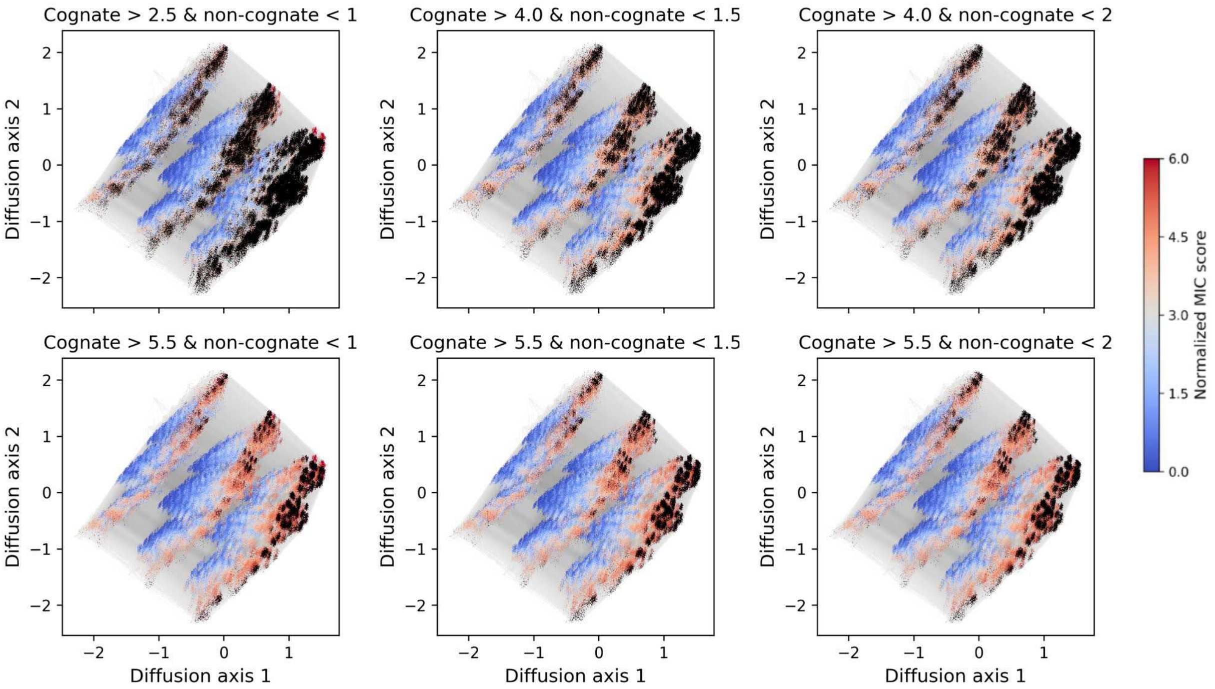

Supplementary Figure 16. Orthogonal pairs to E2/Im2 are robust to different cutoffs for the cognate and non-cognate interactions. Each panel represents the low dimensional visualization of the binding landscapes with genotypes that are orthogonal to E2/Im2 overlaid in black, where orthogonality is defined based on the indicated thresholds on the normalized MIC score for cognate and non-cognate interactions. Note that the number of sequences that are orthogonal to E2/Im2 decrease as the thresholds for viability and non-viability change, but the orthogonal pairs are always concentrated in the same region regardless of the threshold used.

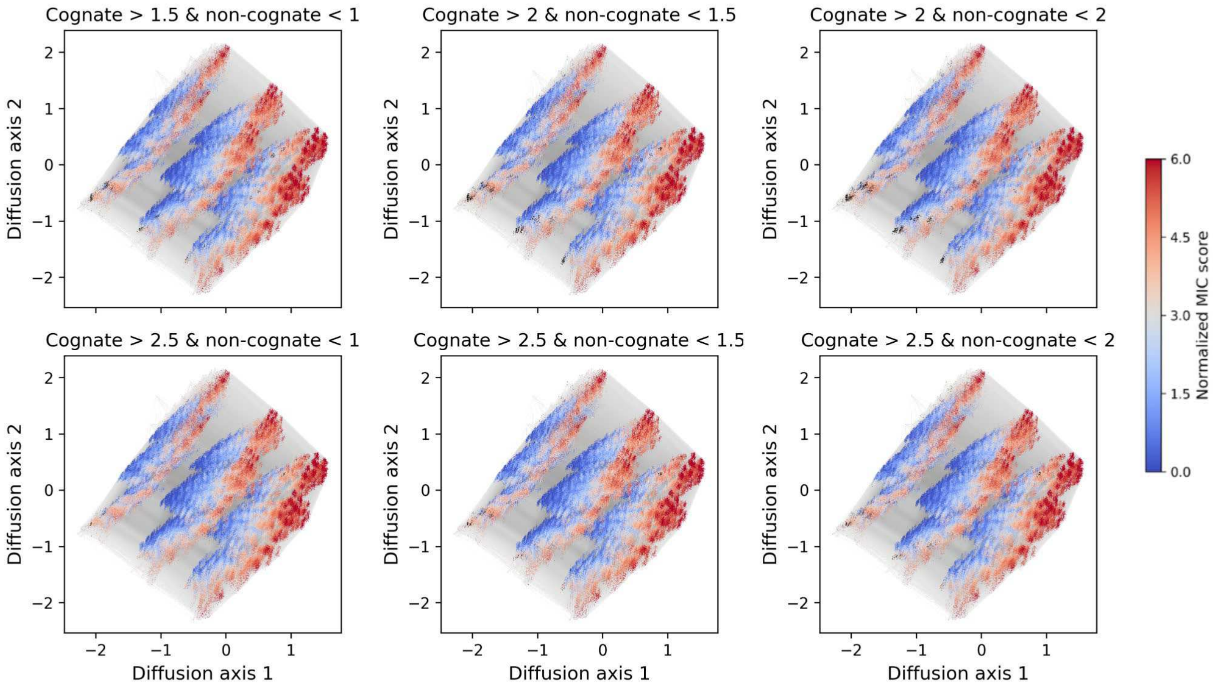

Supplementary Figure 17. Orthogonal pairs to E9/Im9 are robust to different cutoffs for the cognate and non-cognate interactions. Each panel represents the low dimensional visualization of the binding landscapes with genotypes that are orthogonal to E9/Im9 overlaid in black, where orthogonality is defined based on the indicated thresholds on the normalized MIC score for cognate and non-cognate interactions. Note that the number of sequences that are orthogonal to E9/Im9 decrease as the thresholds for viability and non-viability change, but the orthogonal pairs are always concentrated in the same region regardless of the threshold used.

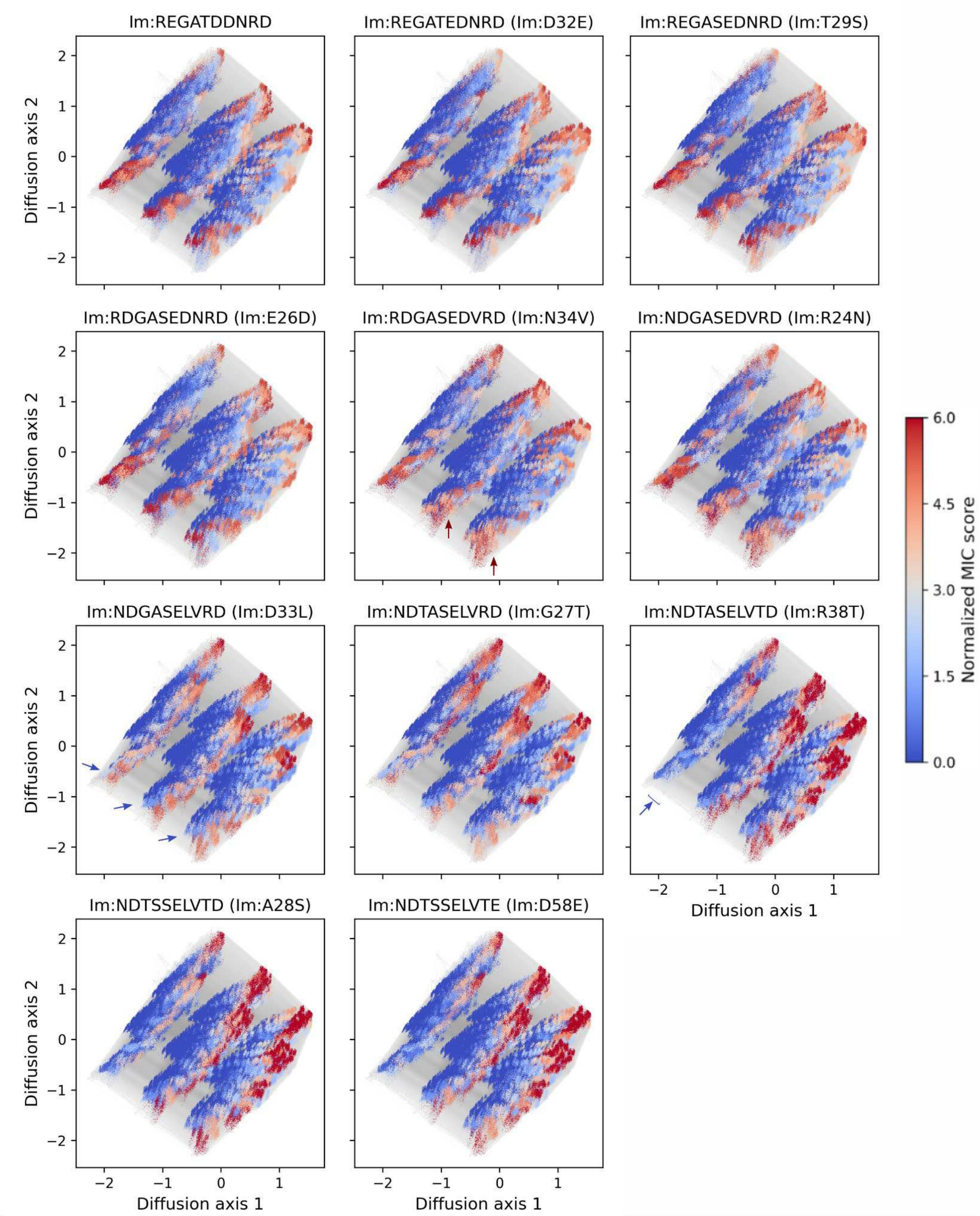

Supplementary Figure 18. Predicted patterns of Im specificity along the experimentally validated viable path between Im2 and Im9. The color scale shows the predicted normalized MIC scores of the Im protein variants along the path when combined with the E subsequence of

every E/Im pair in the landscape. Note that the specificities remain mostly unchanged after each mutation except the key specificity changing mutations: Asn34Val, Asp33Leu and Arg38Thr. Arrows point to regions of sequence space for which the mutation increases (red) or decreases (blue) the normalized MIC score when combined with the E sequence of every pair. To avoid showing interactions with the E proteins of inviable pairs, the score for the interaction with the E protein of any pair with a normalized MIC score below 1 was set to 0.

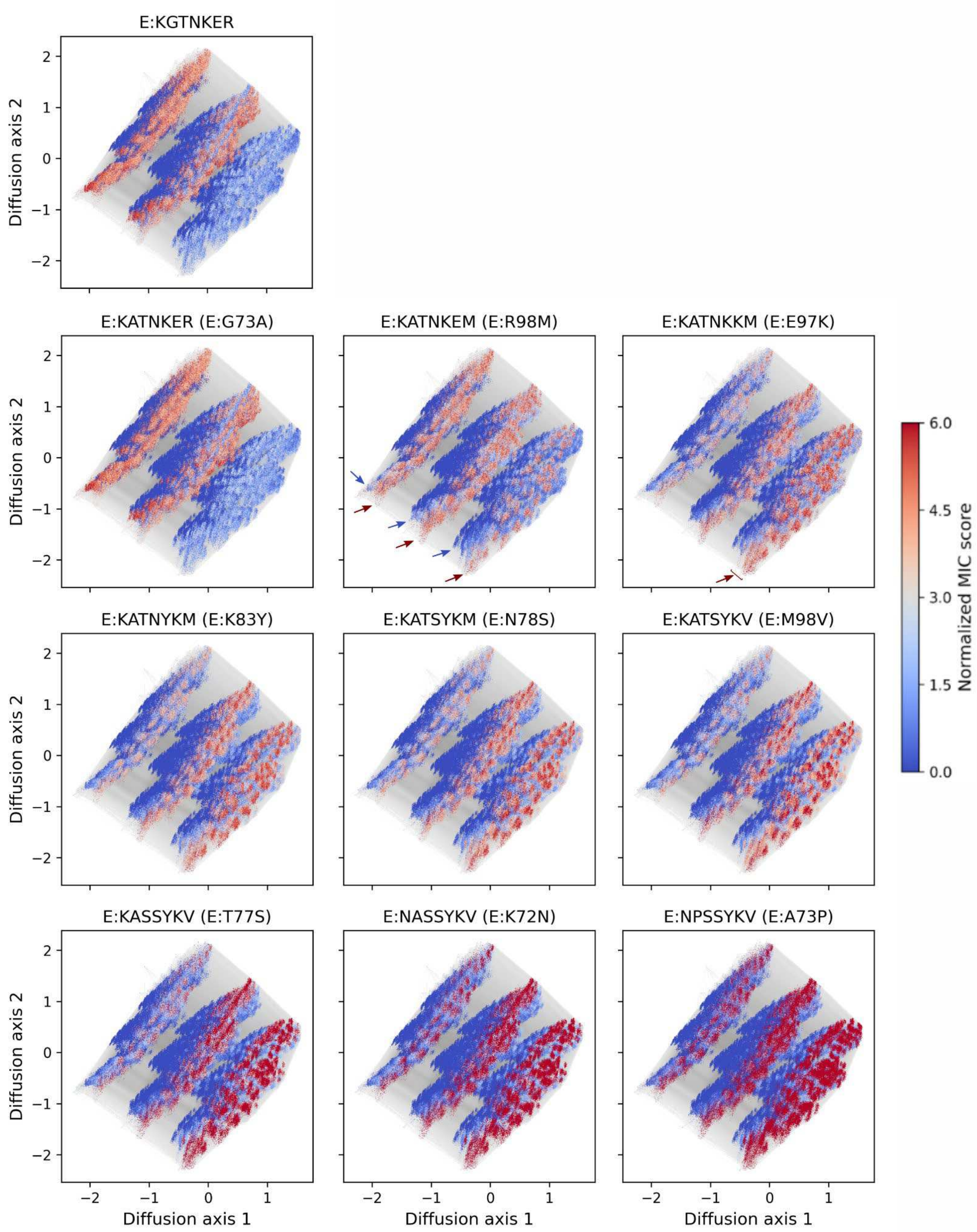

Supplementary Figure 19. Predicted patterns of E specificity along the experimentally validated viable path between E2 and E9. The color scale shows the predicted normalized MIC scores of the E protein variants along the path when combined with the Im subsequence of every

E/Im pair in the landscape. Specificities remain mostly unchanged after each mutation except the key specificity changing mutations: Arg98Met and Glu97Lys. Arrows point to regions of sequence space for which the mutation increases (red) or decreases (blue) the normalized MIC score when combined with the E sequence of every pair. To avoid showing interactions with the Im protein of inviable pairs the score for the interaction with the Im protein of any pair with a normalized MIC score below 1 was set to 0.

Mathematical theory of functional orthogonality in bi-molecular fitness landscapes

In the following section, we aim to formalize the concepts of specificity and orthogonality. Previous work has focused on studying the effects of mutations on cognate and non cognate interactions [51], but here we study these concepts from the more general fitness landscape perspective in order to understand the necessary and sufficient conditions for functional orthogonality to arise in a binding landscape. Moreover, these simple models of orthogonality-encoding landscapes lead to testable predictions about the fitness of unobserved sequences and to a better understanding of the basic underlying molecular mechanisms based on the required patterns of genetic interaction

A pair of molecules A and B can be expressed by the concatenation of the sequences encoding each of the two molecules as a combined genotype A/B, such that the resulting sequence space of all possible pairs is obtained by combining each possible A variant with every B. As every molecule consists of at least 1 site, the sequence of a pair necessarily has length $l \geq 2$. We define the fitness of each pair A_i/B_j as a binary outcome (viable/nonviable) depending on whether the A_i and B_j variants can interact with each other. The specificity of A_i is defined by the set of B_j's such that A_i/B_j is viable and the specificity of B_j is defined as the set of A_i's such that A_i/B_j is viable. Therefore, two variants of A, A_i and A_j, can have different binding specificities depending on the subsets of B molecules with which they interact. In the case that these subsets of B differ, we say that there is a specificity switch along the series of mutations separating A_i from A_j. The existence of such a specificity switch implies that the effect of a mutation in one of the partners A depends on the alleles at the other partner B; that is, epistatic interactions across the binding interface are required for having different specificities in a binding landscape.

Two pairs of molecules A_i/B_j and A_k/B_m, are defined to be orthogonal when both pairs are viable but B_j cannot bind A_k and B_m cannot bind A_i, or in other words, both pairs are viable but their recombinant genotypes (A_i/B_m and A_k/B_j) are nonviable (**Fig. S9A**), a pattern of epistasis that has previously been referred to as a type of "rectangular perturbation"[52]. Importantly, the rectangular perturbation has to involve sites at both sides of the interface. Therefore, regardless of the number of possible combinations of mutations that differ between the two pairs, orthogonality depends only on the fitness of four very specific genotypes. These genotypes are contained within the biallelic landscape defined by all possible allelic combinations present in the orthogonal pairs A_i/B_j and A_k/B_m. That is, we can disregard alleles not present in these two pairs as well as sites that match in the two orthogonal pairs and see how orthogonality arises based only on the number of sites, *a* and *b,* that differ in each of the molecules A and B, respectively, into different *a*:*b* mechanisms or types of orthogonality.

While orthogonality only constrains the fitness values of four possible genotypes, we often study homologous orthogonal pairs. Thus, if we assume that the binding of the two ancestral molecules was required for survival, we expect that the two extant orthogonal genotypes are additionally connected by a path of viable intermediate pairs, imposing additional constraints on the viability

of certain genotypes. Since we are only considering the biallelic sublandscape defining orthogonality, we can characterize the number of plausibly viable shortest paths between orthogonal pairs by counting first the number of nonviable paths, *i.e.*, that go through the nonviable pairs and subtract that number from the total number $(a+b)!$ of possible paths (and $b!$ indicates b factorial). The number of nonviable paths can be easily calculated as the product of the number of paths from each nonviable pair to each of the orthogonal pairs. As the nonviable pairs result from recombination of the orthogonal pairs, we can reach them by mutating all sites in each molecule separately, so they are exactly a and b mutations away from each orthogonal pair. Thus, the total number of shortest paths between the orthogonal pairs that go through nonviable pairs is 2 times $a!b!$, and thus the number of viable paths is $(a+b)!$-2a!b!.

In the following section, we systematically analyze the fitness landscapes under various *a:b* types of orthogonality and the different paths that connect the two orthogonal pairs.

1:1 Orthogonality. In the simplest possible scenario, two orthogonal sequences differ only in one site in each of the molecules. In these settings, the fitness landscape is completely specified by the orthogonality condition directly (0/0 and 1/1 are viable and their recombinants 0/1 and 1/0 are nonviable) and requires reciprocal sign epistasis. However, there is no possible viable path between the two orthogonal pairs (**Fig. S9B**). This matches our expectations of 1+1! possible paths with 2 times 1!1! paths going through the non viable pairs. However, adding an allele or a site to a 1:1 orthogonal landscape can provide a new sequence context that is no longer constrained by the condition for orthogonality and in which a viable path can exist via a mechanism sometimes known as an extradimensional bypass[53]. The addition of a second allele allows the viable path 0/0-0/2-1/2-1/1 through a bridging mutation, while adding a third site provides the viable path 0/00-0/01-1/01-1/11-1/10 with a reversion (**Fig. S9C**). Thus, adding an extra site or allele is the only way for evolving 1:1 orthogonality, but can, in general, provide a way to evolve any *a*:*b* model of orthogonality.

2:1 Orthogonality. In the next simplest case, the two orthogonal genotypes differ by a=2 sites in one of the molecules and b=1 site in the other molecule, resulting in a landscape encoding 2:1 orthogonality. In this case, 00/0 and 11/1 are constrained to be viable while their recombinants 11/0 and 00/1 are necessarily nonviable. Note that, in contrast to 1:1 orthogonality, the recombinants can be at a larger Hamming distance from the orthogonal pairs; *e.g.*, 00/1 is two mutations away from 11/1. Despite these constraints, there are now four unconstrained genotypes that can potentially provide a viable path between the two orthogonal pairs that avoids the nonviable ones *e.g.* 00/0-01/0-01/1-11/1 (**Fig. S9D**). More generally, out of the 3!=6 possible paths, $2 \cdot 2!1! = 4$ go through nonviable genotypes, leaving only two plausible viable paths between the orthogonal pairs. Both of these paths require the mutation in the single-site molecule to happen between the mutations in the two-site molecule, and thus these paths only differ by which of the mutations in the two-site molecule takes place first.

There are two main implications of this mechanism: (1) We show below that this mechanism

requires epistatic interactions between the mutation in the one site molecule and each of the sites in the two-site molecule. Therefore, we expect the two amino acid positions in the two-site molecule to be located close to each other in the three-dimensional structure, since both must interact with the same site in the one-site molecule. (2) Mutations in the viable path take place in a very specific order, since mutating first the single-site molecule will directly take us to one of the nonviable genotypes. Therefore, specificity switching mutations in the two-site molecule need to take place first and last in the path before and after mutation in the single-site molecule.

3:1 Orthogonality. A way to keep expanding our previous model is by adding another site to the two-site molecule, constraining 000/0 and 111/1 to be viable and 000/1 and 111/0 to be nonviable. The number of plausible viable paths between the orthogonal genotypes keeps increasing as there are more unconstrained pairs in the landscape, in the sense that their fitness can be freely assigned without affecting orthogonality. The number of nonviable paths is $2 \cdot 3!1!=12$ in 3:1 orthogonality leaving 12 potentially viable paths. As in 2:1 orthogonality, or more generally, any mechanism of orthogonality with a single site in one of the molecules, the orthogonality constraint implies that the one-site mutation cannot be the first or last mutation to take place in a viable path, so that the mutation in the one-site molecular will always be flanked by mutations in the multi-site molecule both before and after.

2:2 Orthogonality. While the total Hamming distance between the two orthogonal pairs is the same as in the 3:1 orthogonality mechanism, the pairs with constrained fitness values are arranged differently in sequence space (00/00 and 11/11 as viable, and 00/11 and 11/00 as nonviable). As a result, the number of possible viable paths between the orthogonal pairs are increased (**Fig. S9E**) with a total of $(2+2)!-2 \cdot 2!2!=16$ potentially viable paths. However, having for the first time more than one site in each molecule opens the possibility of creating orthogonal landscapes by a simple combination of non-orthogonal landscapes at subsets of sites through a boolean function. In particular, suppose we have two specificity switching (but not orthogonal) biallelic landscapes consisting of 3 viable combinations and only one nonviable intermediate (either 0/1 or 1/0) forming a 1:1 incompatibility. If we define the viable pairs as those requiring both subsets to be viable (AND function) and specifically select 0/1 and 1/0 as non-viable in the two interactions, we obtain a four-site 2:2 orthogonal fitness landscape in a type of orthogonality that we called (1:1)&(1:1). Importantly, 00/11 and 11/00 are non-viable, as each of them contains one of the non-viable intermediates in each subset of sites, or what is equivalent, containing one of the specific alleles in one of the 1:1 incompatibilities. Moreover, the orthogonal pairs (00/00 and 11/11) are connected, not only by a single path, but they occupy the corners of a 3x3 grid of viable pairs providing 6 different viable paths between the orthogonal pairs (**Fig. S9F**).

Here, we aimed to describe and characterize some of the simpler models of orthogonality, but mechanisms involving a larger number of sites are certainly possible. As shown for the 2:2 model, a natural and parsimonious way in which orthogonality involving a larger number of sites appears is by combining smaller intermolecular interactions or incompatibilities that do not necessarily encode orthogonality independently. This phenomenon generates orthogonality through the AND

function, which makes the recombinant pairs that contain only a subset of the required interactions nonviable. Another simple way is by having additional neutral sites differing between the orthogonal pairs. This would increase the number of pairs that are orthogonal to each pair as well as the viable paths between them, as the number of contexts in which specificity switching mutations can happen would increase. Thus, even if two orthogonal pairs differ in a relatively large number of sites in their interface, if there are only a few specificity-switching mutations, these simple mechanisms or combinations of them can realistically explain the evolution of orthogonality and the geometry of the fitness landscape. For example, in the empirical path, the closest orthogonal pairs differ by 4 mutations, but it corresponds to a 2:1 orthogonality because one of the mutations (Im:Arg24Asn) is largely neutral.

Moreover, and as orthogonal landscapes involve more sites, different mechanisms driving orthogonality could coexist, *e.g.*, if instead of combining two intermolecular biallelic interactions as in the (1:1)&(1:1) model (**Fig. S9F**), we replace one of those interactions by a 2:1 orthogonality landscape, the resulting (2:1)&(1:1) landscape shows both 2:1 orthogonality within one set of sites, but could simultaneously show 2:2 orthogonality through a (1:1)&(1:1) mechanism involving only two of the 3 sites required for the 2:1 orthogonality. As a consequence, under selection for orthogonality to a particular pair in a fitness landscape with coexisting modes of orthogonality *e.g.* (2:1)&(1:1), orthogonality could first arise via one mechanism, *e.g.*, 2:1, which requires fewer mutations, but be maintained at some point via another mechanism, *e.g.* 2:2, without ever losing orthogonality. In fact, the predicted fitness landscape (**Fig. 3A**) is largely consistent with a (2:1)&(1:1) orthogonality where the subsets of interacting sites are E:98/Im:33-34 and E:97/Im:38. The empirical path switches specificity through the 2:1 orthogonality mechanism alone, but, after resolving the second specificity-changing interaction E:97/Im:38, the Im:Asp33Leu mutation may be reversed without losing orthogonality to E2/Im2 (though with potential loss in affinity and stability), because orthogonality is predicted to be maintained via a 2:2 mechanism involving E:98/Im:34 and E:97/Im:38. Another simple class of models with coexisting modes of orthogonality can arise from combining more than 2 intermolecular interactions in a more complex way *e.g.* requiring at least 2 viable interactions such as in a (1:1)&((1:1)|(1:1)) model, where "|" indicates logical OR. In this case, we have 3 intermolecular interactions but 2 of them are sufficient for the interaction to happen. Then, we can easily see how orthogonality can arise first via 4 mutations affecting the 2 first interactions, but after mutations involving the 3rd interaction take place, the mutations at the second set of sites can be reverted without losing orthogonality to the original pair.

Epistasis in orthogonal landscapes. In this section we study how the rectangular interaction imposed by the orthogonality constraint and the viable path requirement impact local double and triple mutant epistatic coefficients in the fitness landscape. We use the definition of background-dependent epistatic coefficient with our binary fitness values:

$$\varepsilon_2 = (f_{11} - f_{01}) - (f_{10} - f_{00}).$$

Thus for the 1:1 orthogonality model we have $\varepsilon_2 = 2$. If we allow a viable path through a bypass mutation at a third bi-allelic site, it creates an opportunity for a 3-way interaction, which corresponds to the difference in pairwise epistatic coefficients between parallel faces, giving rise to the local three-way epistatic coefficient:

$$\varepsilon_3 = \Big(\big(f_{11/1} - f_{11/0}\big) - \big(f_{10/1} - f_{10/0}\big)\Big) - \Big(\big(f_{01/1} - f_{01/0}\big) - \big(f_{00/1} - f_{00/0}\big)\Big).$$

Thus, we can ask whether a 3-way interaction is necessary to have a viable path between the orthogonal pairs. Under the 1:1 orthogonality mechanism with a bypass mutation, there is a single unconstrained genotype: 10/1 or 11/0 depending on the viable path we choose; so we can easily calculate the 3-way epistatic coefficient as a function of this gentoype's fitness:

$$\varepsilon_3 = \Big((1 - 1) - \big(f_{10/1} - 1\big)\Big) - \big((1 - 0) - (0 - 1)\big) = -1 - f_{10/1} < 0\,.$$

Since $f_{10/1} \in \{0, 1\}$, the 3-way epistatic coefficient must be negative, and therefore a 3-way interaction is required to allow a viable path between the orthogonal pairs through a 3rd site bypass.

The 2:1 orthogonality model with viable path constrains the fitnesses of 6 genotypes in the landscape spanning two adjacent faces of the cube, and therefore establishes a requirement on the pairwise epistatic coefficients of each of them. Specifically, it implies that the one site molecule establishes a pairwise interaction with each site at the other molecule with pairwise epistatic coefficients of opposite signs.

$$\big(f_{11/1} - f_{11/0}\big) - \big(f_{01/1} - f_{01/0}\big) = 1$$

$$\big(f_{00/1} - f_{00/0}\big) - \big(f_{01/1} - f_{01/0}\big) = -1$$

We next ask if 2:1 orthogonality requires a 3-way interaction. As before, we calculate the 3-way epistatic coefficient, which now depends on the fitnesses of the 2 remaining unconstrained genotypes:

$$\varepsilon_3 = \Big((1 - 0) - \big(f_{10/1} - f_{10/0}\big)\Big) - \big((1 - 1) - (0 - 1)\big) =$$

$$= f_{10/0} - f_{10/1}$$

Thus, we can see that 2:1 orthogonality is compatible with a landscape without 3-way interactions as long as mutating the one-site molecule is neutral when paired with the 10 background in the two site molecule $\big(f_{10/0} = f_{10/1}\big)$. That still leaves open the possibility of an *intra*molecular pairwise interaction, which would happen if $f_{10/0} = f_{10/1} = 1$ but not if $f_{10/0} = f_{10/1} = 0$. The former allows having two viable paths between the orthogonal pairs, while the latter minimizes the number of required epistatic interactions to have 2:1 orthogonality with a single viable path. Thus, the 2:1 orthogonality mechanism requires a pair of inter-molecular pairwise epistatic

interactions but is also compatible with the presence of an intramolecular pairwise epistatic interaction in the two-site molecule or the presence of a three-way epistatic interaction across both molecules.

Supplementary Files

This is a list of supplementary files associated with this preprint. Click to download.

- Supplementaryinformation1223.docx
